# Prioritizing Tiger Conservation through Landscape Genetics and Habitat Linkages

**DOI:** 10.1371/journal.pone.0111207

**Published:** 2014-11-13

**Authors:** Bibek Yumnam, Yadvendradev V. Jhala, Qamar Qureshi, Jesus E. Maldonado, Rajesh Gopal, Swati Saini, Y. Srinivas, Robert C. Fleischer

**Affiliations:** 1 Wildlife Institute of India, Chandrabani, Dehradun 248001, India; 2 Smithsonian Conservation Biology Institute, National Zoological Park, 3001 Connecticut Avenue, Washington, D.C. 20008, United States of America; 3 Department of Vertebrate Zoology, National Museum of Natural History, Smithsonian Institution, Washington, D.C. 20013, United States of America; 4 National Tiger Conservation Authority, Bikaneer House, Shah Jahan Road, New Delhi 110011, India; University of Illinois at Urbana-Champaign, United States of America

## Abstract

Even with global support for tiger (*Panthera tigris*) conservation their survival is threatened by poaching, habitat loss and isolation. Currently about 3,000 wild tigers persist in small fragmented populations within seven percent of their historic range. Identifying and securing habitat linkages that connect source populations for maintaining landscape-level gene flow is an important long-term conservation strategy for endangered carnivores. However, habitat corridors that link regional tiger populations are often lost to development projects due to lack of objective evidence on their importance. Here, we use individual based genetic analysis in combination with landscape permeability models to identify and prioritize movement corridors across seven tiger populations within the Central Indian Landscape. By using a panel of 11 microsatellites we identified 169 individual tigers from 587 scat and 17 tissue samples. We detected four genetic clusters within Central India with limited gene flow among three of them. Bayesian and likelihood analyses identified 17 tigers as having recent immigrant ancestry. Spatially explicit tiger occupancy obtained from extensive landscape-scale surveys across 76,913 km^2^ of forest habitat was found to be only 21,290 km^2^. After accounting for detection bias, the covariates that best explained tiger occupancy were large, remote, dense forest patches; large ungulate abundance, and low human footprint. We used tiger occupancy probability to parameterize habitat permeability for modeling habitat linkages using least-cost and circuit theory pathway analyses. Pairwise genetic differences (*F*_ST_) between populations were better explained by modeled linkage costs (*r*>0.5, *p*<0.05) compared to Euclidean distances, which was in consonance with observed habitat fragmentation. The results of our study highlight that many corridors may still be functional as there is evidence of contemporary migration. Conservation efforts should provide legal status to corridors, use smart green infrastructure to mitigate development impacts, and restore habitats where connectivity has been lost.

## Introduction

By virtue of being at the top of the food chain, large carnivores occur at low densities, have large home ranges and therefore require vast areas to harbor viable populations [1]. Since historical times, large carnivores have been in conflict with humans for food and resources, often resulting in their demise [2]. Habitat destruction, excessive hunting by humans and the use of body parts for traditional medicine have extirpated many populations [3], [4], [5] while severely reducing, fragmenting, and isolating most others to varying degrees [6], [7]. Small and isolated populations are prone to local extirpation [8], [9]. Managing such populations in a metapopulation framework [10], [11] by connecting them through habitat corridors [12], [13] so that individuals have the opportunity to disperse, establish residency and reproduce, reduces the overall risk of extinction [1], [14]. Much of the global conservation policy on endangered species is centered on land allocation schemes for securing source populations [15], promoting and maintaining connectivity between fragmented populations [16], [17]. Land is one of the most prized resources, and a major challenge to this conservation approach is the difficulty in convincing governments and policy makers on its allocation for conservation purposes. This problem is further compounded when objective criteria for delineating corridor habitats or documenting their functionality based on rigorous scientific data are lacking. As a result, conservation relies heavily on expert opinion and models of corridor connectivity that have little empirical validation.

The tiger (*Panthera tigris*) acts as a flagship species for the conservation of forested ecosystems throughout its range in Asia [18]. Conserving the tiger typifies the prospects and challenges inherent in the current paradigm of fragmented small populations and landscape based conservation models in large carnivores [19]. Extant tiger populations are confined to less than seven percent of their historical range in patchily distributed habitats across a range of twelve regional tiger conservation landscapes (TCLs) in southern and north-eastern Asia [20]. Six global priority TCLs of long-term tiger conservation significance are present in the Indian subcontinent. These Indian TCLs are important for global tiger recovery as they harbor over 50% of the estimated global population of ∼3,000 wild tigers [21], [22], and>60% of the global genetic variation in the species [23]. The high genetic variation seen in Indian tigers could be attributed to historically high population sizes, numbering about 50,000 individuals until *c*. 200 years ago, and habitat contiguity that permitted genetic exchange between the various regional tiger populations in the area [23]. Due to change in land ownership and forest use policy in the mid nineteenth century during British rule and again during the early years of India's independence a century later, much of the forest was cleared for timber and agricultural needs [24], [25]. This change in land use combined with organized trophy hunting and bounty driven extermination resulted in severe decline, fragmentation and isolation of tiger populations throughout India [25], [26]. Tiger conservation and subsequent population recovery in India began during the 1970s with the creation of a number of protected areas (Tiger Reserves) under the Project Tiger network in 1973 [18], and aided by comprehensive wildlife legislation (Wildlife Protection Act 1972, [27]). Under Project Tiger, the tiger was used as a flagship and umbrella species for conserving the biodiversity of India's forested ecosystems. However, even though extensive areas have been added to the protected area network, the future of tigers is under severe threat from commercial poaching, and extensive habitat fragmentation within the last two decades [15], [18], [21]. The rise in human-wildlife conflict and issues dealing with land rights of forest-based dwellers, as people are present both inside and outside tiger reserves, further vexes grass-roots conservation especially at a landscape-scale, and negatively impacts tiger dispersal capability and survival within TCLs [28]. These factors have precipitated the systematic decline in tiger and prey populations from both outside and inside reserves, as attested by the recent local extirpations in few areas [5], [29].

Currently in India, the once contiguous tiger population is now fragmented with source populations primarily restricted to tiger reserves. A tiger reserve is legally mandated to designate a critical core area wherein human habitation and resource extraction is not permitted (Wildlife Protection Act 1972, amendment 2005 [30]). This core is surrounded by a buffer zone, which is essentially a multiple use area, wherein conservation objectives are to be given precedence over other land uses. Breeding populations of tigers are mostly located in the core area of tiger reserves, while the buffers usually serve as population sinks [22], [28], [31]. The size of these tiger reserves vary between 344 km^2^ to 3,150 km^2^ (average 1,321 km^2^), with tiger densities ranging from about 0.1 to 20 individuals per 100 km^2^ [22], [31], [32]. For a demographically viable tiger population, a minimum of 20 to 25 breeding units are believed to be essential [15], [32], [33]. As such, many extant tiger populations are by themselves inadequate for long-term persistence [33], [34], either because of habitats harboring a low number of breeding tigers, small size of the protected area and/or ecological isolation from other populations. High spatial genetic structuring and small population size observed in today's Indian tiger populations [35] dictates preserving them in a metapopulation framework wherein individual populations are connected through a permeable habitat matrix and can occasionally exchange individuals [36], [37]. This would result in re-colonization of suitable habitat patches where tigers have become locally extinct and ‘rescue’ declining local populations from extinction by immigrants [37], [38]. Understanding and managing the metapopulation framework of extant tiger populations is an important strategy for ensuring their long-term conservation. This approach entails strict preservation of source populations in protected areas and informed conservation strategies across tiger landscapes.

Due to the relatively high *K* selected life history traits of the tiger in comparison to other large cats, dispersal and immigration play a vital role in long-term viability of tiger populations [32]. Incidentally, it was likely due to the ‘rescue effect’ by immigrants from high-density populations and intact habitat corridors in the vicinity of Chitwan National Park, Nepal, which enabled the tiger population in the park during the 1930s to recover, even after heavy trophy hunting, to pre-decline levels in only three years [32]. In recent times, tigers have successfully recolonized Rajaji National Park, India, in the Shivalik-Gangetic Plain landscape, from connected source populations further east, within a decade of having completely disappeared from the area [39]. Small tiger populations that become isolated are likely to face extinction due to demographic stochasticity, inbreeding depression [40] and deterministic factors such as poaching [32], [33], as witnessed in the small and isolated Indian tiger reserves of Sariska and Panna which recently suffered from local extinction events, although tigers were later re-introduced [5], [29]. Habitat connectivity is integral to sustaining regional populations of tigers, as they require contiguous forest connectivity for dispersal and genetic exchange between populations [41]. Currently, within the six tiger occupied landscapes of India, habitat contiguity varies extensively, with the best being within the Western Ghats and the North East, while fragmentation is highest in the Shivalik-Gangetic Plain and the Central Indian Landscapes [42]. Most of the connecting habitats in these landscapes are not within the legal domain of protected areas and are often lost to burgeoning development demands of a growing economy and attrition by human consumptive uses. In India, the transfer of forest-land to other land uses requires approval from the Federal Government as outlined in the Forest (Conservation) Act 1980 [43]. Since Federal Government approvals are usually sought on a case-by-case basis, and rarely are the cumulative impacts of projects or landscape scale conservation significance of forest patches factored into decision making, such permissions are frequently granted [44]. However, when the Supreme Court of India and Federal Government Committees were presented with concrete scientific evidence on the significance of conserving these forest patches, development projects even of national interests have been stalled [45], [46], [47]. Unfortunately, scientific data rarely exist to substantiate the landscape-level conservation significance of forest patches that constitute habitat corridors, and crucial areas are often lost. Studies on spatial dispersal and gene flow to detect population units and migration between patches can provide a quantitative and formal assessment of corridor function and identify priority populations for conservation action.

Assessing gene flow in species across populations in complex fragmented habitats is critical to understand how landscapes structure genetic variation and maintain metapopulation connectivity. Unfortunately, the traditional validation of habitat connectivity through the direct observation of individual animal movement is logistically difficult as it would entail following the fates of many radio-collared or camera trapped individuals over a regional scale and spanning multiple generations. As a result, alternative genetic assignment methods based on individual clustering approaches [48], [49] have gained popularity [7], [50]. The integration of metapopulation genetic models with spatial analytic tools in a landscape genetics framework provides a quantitative approach for understanding the role of geography, habitat and land-use features either as barriers or facilitators to gene flow among natural populations [51]. Though initially restricted to analyses correlating with linear distances [51], [52], the developing field of landscape genetics has now advanced to include more complicated connectivity modeling incorporating ways in which habitats are actually traversed in nature. The use of landscape heterogeneity patterns and habitat permeability obtained from Geographical Information Systems (GIS) layers to model habitat connectivity by least-cost pathway (LCP) analysis [53] and circuit theory based isolation-by-resistance (IBR) model [54], [55] that permit gene-flow between populations provide an objective criteria for delineating and prioritizing habitat corridors. There is a small but rapidly growing body of literature investigating the relationship between genetic and corridor connectivity, with both LCP and IBR models finding promise in gene flow studies on taxa with lower dispersal capabilities and that readily form visible metapopulations such as amphibians (tiger salamanders, *Ambystoma sp.* [56], [57]), to wide-ranging carnivore species (cougar, *Puma concolor* [58]; bobcat, *Lynx rufus* [59]; wolverine, *Gulo gulo* [60]; black bear, *Ursus americanus* [61]). Where available, researchers have incorporated information from animal habitat use and movement behavior in the cost parameterization schemes to approximate realistic paths of least resistance, as in Reding *et al.* [59]. However, such data are not readily obtainable, and hence the vast majority of studies rely on expert opinion and *a priori* assumptions on animal presence to assign cost schemes and parameterize landscape resistance to gene flow. Although informative, the parameterization schemes used in landscape resistance surfaces to model movement paths and the assignment of cost schemes to grids in GIS rasters could easily introduce biases which may be more reflective of habitats as perceived by humans rather than by animals [62], [63]. Incorporating information obtained from fine-scale species and landscape-specific ground data on suitable habitat, cover, prey availability, disturbance and threats in considering the attribute of surrounding cells, is one way which could help reduce subjectivity involved when assessing resistance or cost of a cell and the likelihood of path usage [63].

In this study, we investigate patterns of landscape heterogeneity and spatial genetic structuring to identify barriers and minimal habitat corridors for gene flow between populations within the fragmented tiger habitats in Central India. The Central Indian landscape is a globally recognized area for tiger conservation, with significant potential for long-term persistence of the species [15], [31]. The area supports one of the largest global concentrations of tiger populations (∼20% of an estimated 1,700 adult Indian tigers, [31]) in patchily connected habitats. Although the populations were historically connected, rapid infrastructural development and urbanization in recent years threaten to form permanent barriers to dispersing tigers by isolating tenuously connected small populations, thereby effectively reducing long-term metapopulation persistence. Recent population and spatial genetic studies have observed low genetic structure among populations indicative of gene flow [64], [65] and long-range dispersal which are affected by increasing urbanization in the area [66]. Although tigers can move huge distances in undisturbed habitats [67], the complex fragmented habitat mosaic in the area, interspersed with high density human settlements and increasingly urbanized centers, have generally been thought to limit long-range dispersal [41]. Dispersal in tigers, like in lions (*Panthera leo* [19]) is male biased, as female offspring tend to reside and breed close to their maternal ranges, while male offspring disperse long distances and establish home ranges far from their natal areas [41]. This study explores a strategy that utilizes genetic assignment methods to detect population genetic structuring and determine which populations are in migratory contact, extensive occupancy modeling and GIS analysis to delineate structural connectivity between populations, and a correlation process between landscape connectivity versus population pairwise genetic distances to determine which of the movement cost schemes and modeled corridors best explain the observed genetic structuring in the area.

We extensively and intensively collected scat and a few tissue samples across seven tiger reserves in the Central Indian Landscape and first identify tiger individuals by genotyping the DNA extracts using eleven autosomal microsatellite loci. Next we assessed spatial genetic structuring and gene flow in the identified individuals through individual clustering methods. We use likelihood based [49] and Bayesian [48], [68] assignment methods to detect first and second generation migrants between the identified genetic population clusters. Since resident tigers do not occur outside of forested habitat, we surveyed all of the forested area (76,913 km^2^) within 185,100 km^2^ of Central India. Based on our understanding of tiger ecology, we predicted *a priori* that tigers should occur in vast, undisturbed, productive forest patches, with high density of large wild ungulate prey, which would be negatively impacted by human disturbances [20], [31], [41], [67]. We tested these *a priori* hypotheses by spatially explicit modeling of tiger occupancy that accounted for imperfect detections, using covariates obtained by remote sensing and ground surveys covering all forest patches within our study area. We then used this spatially explicit information of tiger occupancy as a resource selection probability function [69], [70] to model habitat corridors joining tiger populations using LCP [71] and circuit theory [72] analyses in a GIS setting. With genetic data we tested if the observed population structure and dispersal between populations is in concordance with ground reality of tiger occupancy and existing habitat connectivity. Our comprehensive study highlights the importance of particular tiger source populations and intervening forest corridors for maintaining metapopulation structure within Central India. It provides a basis to formulate conservation policy and assist informed decision making for land-use planning at the landscape scale.

## Materials and Methods

### Ethics Statement

The majority of field sampling was conducted non-invasively from tiger scat, without animal capture and handling. Permits for collection of tiger scat samples were obtained from the National Tiger Conservation Authority and the State Forest Departments. Capture and radio collaring of tigers required the approval of the Ministry of Environment and Forests, Government of India and the Chief Wildlife Warden, Madhya Pradesh State, under the Wildlife (Protection) Act 1972. The permissions define the conditions required for capture of tigers, which include an approved protocol and participation by a Park Official and supervision by a qualified veterinarian in the capture and collaring exercise. Both these permissions were obtained and strictly adhered to. Capture operations were conducted by trained veterinarians and wildlife biologists as per the protocols of the Wildlife Institute of India and the National Tiger Conservation Authority. A tiger tissue sample was obtained from Satpura Tiger Reserve where the tiger died due to natural causes (was killed by another tiger in a territorial strife). This research project was conceived and radio collaring reported in this paper was done prior to the formation of an animal ethics committee at the Wildlife Institute of India.

### Study Area

**Figure 1:**
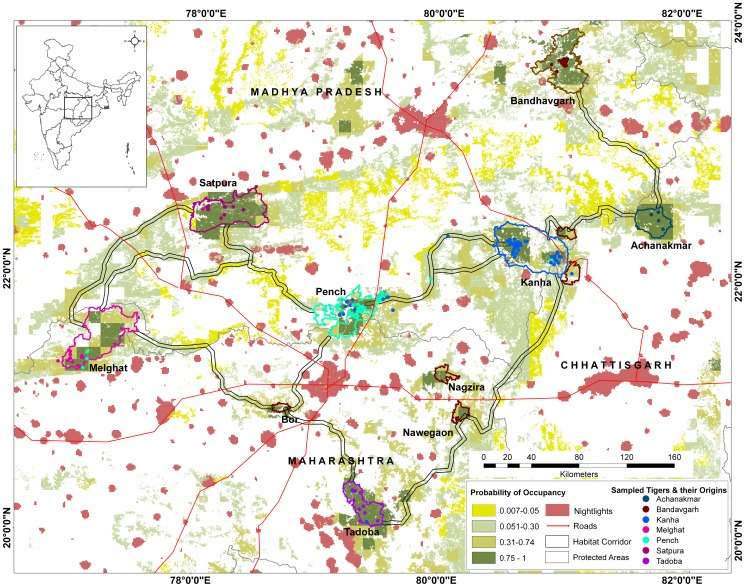
Study area map showing sampled sites, genotype locations and habitat connectivity. The study area of Central India spanning the states of Madhya Pradesh, Maharashtra and Chhattisgarh, showing tiger habitat (forest cover) coded with tiger occupancy probability, protected areas, human habitation (night lights), major roads and least-cost habitat corridors connecting tiger reserves. Individually genotyped tigers (n = 165) are shown as color coded dots at their sampled locations with their colors matching their genetically assigned population.

The present study was carried out in the global priority tiger conservation landscape of Central India within the states of Madhya Pradesh, Maharashtra, and Chhattisgarh (Figure 1). A forested area of 76,913 km^2^ (20.1–23.5°N and 76.5–81.5°E) covering the seven tiger reserves of Melghat, Satpura, Pench, Kanha, Tadoba, Achanakmar, and Bandhavgarh along with their buffer zones, corridor habitats and adjoining forested habitats were sampled. Sampled sites covered different types of tiger habitats found in Central India ranging from the tropical moist-deciduous Sal (*Shorea robusta*) forests in Kanha and Bandhavgarh to tropical dry-deciduous teak (*Tectona grandis*) dominated forests in Pench, Tadoba and Melghat. The topography varied from about 200 meters above sea level (m a.s.l.) in the low-lying hills to the *dadar* plateaus and meadows in Kanha (500 m a.s.l.) and the rugged Satpura ranges (highest elevation 1,352 m a.s.l.). The rainfall, primarily restricted to the monsoon season (late June to September end) ranged between 1,000 to 2,200 mm per year. The large mammal fauna found in the region included tigers (*Panthera tigris*), leopards (*P. pardus*), sloth bears (*Melursus ursinus*), dholes (*Cuon alpinus*), gaurs (*Bos gaurus*), wild pigs (*Sus scrofa*), sambar deer (*Rusa unicolor*), chital deer (*Axis axis*), barking deer (*Muntiacus muntjak*), swamp deer (*Rucervus duvaucelii*) and nilgai antelopes (*Boselaphus tragocamelus*).

### Genetic Sampling and Laboratory Work

Blood from 16 radio-collared tigers, one tissue sample from a dead individual and 587 putative tiger scat (faeces) samples were collected between 2006 and 2011 from the seven Tiger Reserves, and at a few intervening forest corridors in the area (Figure 1 and Table 1). The number of samples obtained was largely proportional to the population size of tigers in that region. However, due to logistical constraints the Tadoba population was under sampled. Scats were stored, either dry with silica or in 75% ethanol, and kept at ambient temperature, prior to laboratory analysis. For each scat, a Global Positioning System (GPS) reading was taken and transferred into a GIS. Scat DNA extractions were performed in a room dedicated to low-copy DNA extraction, using the guanidine isothiocyanate - silica extraction protocol [73]. For every extraction, negative controls composed of reagent only without the scat sample were included to monitor contamination. Extractions from blood and tissue samples were carried out using the DNeasy blood and tissue kit (QIAGEN, Germany).

**Table 1:** Area-wise estimates showing population extents, occupied habitats, sampling effort and number of tiger individuals identified.

Population	Naïve Occupancy (km^2^)	Critical Core Area (km^2^)	Tiger Reserve Size (km^2^)	total scats/cyt *b* PCR amplified/tiger scats/STR genotypes	other samples -blood (b), tissue (t)	all samples/all tiger STR genotypes/individuals	estimated population size (SE range) *	% population sampled	identified tiger individuals		
									male	female	unknown sex
Melghat (M)	2,376	1,501	2,769	66/47/44/35	0	66/35/15	35 (30–39)	43	6	8	1
Satpura (S)	1,554	1,339	2,133	46/21/17/17	1 (t)	47/18/11	43 (42–46)	26	3	6	2
Pench (P)	2,174	669	1,921	137/94/86/82	3 (b)	140/85/51	65 (53–78)	78	25	24	2
Kanha Pench corridor (KPC)	1,013	*NA*	*NA*	26/24/5/5	0	26/5/5	*NA*	*NA*	2	2	1
Kanha (K)	1,850	917	2,052	159/67/65/56	12 (b)	171/68/50	60 (45–75)	83	22	27	1
Achanakmar (A)	904	626	914	23/16/5/5	0	23/5/4	12 (11–13)	33	2	2	0
Tadoba (T)	3,519	626	1728	32/20/17/17	0	32/17/11	69 (66–74)	16	5	3	3
Bandhavgarh (B)	1,700	717	1,537	98/41/36/33	1 (b)	99/34/22	59 (47–71)	37	9	13	0
**Total**	**15,090**	**6395**	**13,054**	**587/330/275/250**	**17**	**604/267/169**	**343**	**49**	**74**	**85**	**10**

* in Jhala *et al.* [31], *NA* - not available.

Tiger scats were identified from among the field collected scats through PCR and *Bam*HI restriction enzyme digestion of the mitochondrial DNA cytochrome *b* gene (Figure S1; see Methods S1 and Tables S10 and S11 for details on protocols and reference species used for PCR). Unambiguously assigned tiger scats and blood and tissue samples were individually identified using a panel of eleven microsatellite loci, derived from domestic cat [74] and tiger [75], [76]. The loci consisted of three dinucleotide (Fca304, Fca954, 6Hdz700), three trinucleotide (Pati01, Pati09, Pati15) and five tetranucleotide repeat markers (Fca441, F85, F53, F124,Pati18), variously labeled at the 5′ end of each forward primer with 6–FAM, PET, VIC and NED dyes. Gender identification in individual tigers was carried out by amplifying the Y chromosome linked SRY gene and an X chromosome microsatellite locus Fca651. PCR amplifications were carried out in 10 µl reactions with a multiple panel of 3 to 4 loci using the Multiplex PCR kit (QIAGEN) according to the manufacturer's instructions. Amplified products were resolved on the ABI 3130 Genetic Analyzer and GENEMAPPER 3.7 (Applied Biosystems, USA) was used to score allele sizes. To limit genotyping error due to allelic dropout (nonamplification of one allele in a heterozygote), multiple PCR replicates were conducted as in Navidi *et al.* [77]. Heterozygotes were confirmed with at least two independent replicates and homozygotes with five replicates. The genotype data were checked on MICROCHECKER [78] for identifying and correcting genotyping errors such as those that arose from stuttering patterns, null alleles and small-allele dominance.

### Identification of Individuals and Descriptive Statistics

In order to investigate the power of the eleven microsatellite loci to distinguish among closely related individuals in the same population, the conservative sibling probability of identity (*PI sib*) statistic [79], [80] was computed in GIMLET 1.1 [81]. Unique multilocus genotypes were identified using the *Identity* analysis option in CERVUS 3.0 [82], [83]. Samples that showed mismatches at up to two loci were re-examined for possible genotyping errors and allelic drop-out, and again amplified thrice in order to confirm the multilocus genotypes before assigning them as unique individuals. Multiple replicates of the same individual from the same locality were discarded and only unique multilocus genotypes were used for all further analyses. We used CERVUS 3.0, GENEPOP 4.1 [84], [85], GENALEX 6.3 [86] and FSTAT 2.9.3 [87] to calculate the following descriptive statistics: (i) number of alleles per locus, (ii) observed and expected heterozygosity, (iii) tests for deviation from Hardy-Weinberg Equilibrium (HWE), (iv) significance values for linkage disequilibrium (LD) among all pairs of loci, and (v) estimates of population pair-wise *F*_ST_ [88] and *R*_ST_ [89] values. Loci with null alleles were flagged by MICROCHECKER, and we tested for deviation from HWE using both null allele adjusted and unadjusted (observed allele frequencies) genotypes. We also tested for associations between genetic variation and estimated population size through Spearman's rank correlation using the pspearman package [90] in R (http://cran.r-project.org).

### Population Genetic Structure

We used two types of individual-based analyses to assess genetic differences among individuals and assignment patterns of tigers to populations. First, a Principal Coordinates analysis (PCoA) based on pair-wise *Phi*_PT_ genetic distances [91] was carried out in GENALEX and the scatter of population-wise individual assignments was plotted on the first three PCo axes using NCSS (www.ncss.com) to understand broad spatial patterns of populations structure in the landscape. Next, we used the Bayesian individual clustering approach in STRUCTURE 2.3.3 [48] to detect population structure among sampled localities in the area by assigning sampled individuals into a number of clusters (*K*) based on the multilocus genotype data alone. The clustering process ensures that for identified population clusters, deviations from Hardy Weinberg and linkage equilibrium are minimized. We analyzed our data in STRUCTURE by using the admixed model and correlated allele frequencies option to carry out thirty independent simulations at each *K* (*K* = 1 to 10), with a burn-in length of one million Monte Carlo Markov Chain (MCMC) steps and data collection phase of five million MCMC iterations. These run times were sufficient to ensure convergence of the Markov chains, and all runs were carried out both with (locprior  = 1) and without (locprior  = 0) using prior sampling locality information. The true *K* or most likely number of population clusters in the dataset was inferred from (i) the *ad hoc* parameter of log-likelihood change in probability of individual assignments to *K* clusters (*Ln P(K)*, [48]), and (ii) the second order rate of change in the likelihood of *K* values (*delta K*, [92]). Both these values were computed from the STRUCTURE output using the program STRUCTURE HARVESTER v0.6.91 [93]. We also carried out an Analysis of Molecular Variance (AMOVA, [91]) in GENALEX to compare the population clusters identified by the STRUCTURE analysis. Genetic variances were partitioned at two levels, viz. among all the STRUCTURE identified population groups (*K* clusters) and among subpopulations within each group.

### Detection of Migrants

We used three Bayesian approaches implemented in STRUCTURE 2.3.3 [48], GENECLASS 2.0 [49] and BAYESASS 1.3 [68], for identification of migrant and admixed individuals. STRUCTURE was used to calculate the posterior probability of whether individuals are residents of their sampled population or migrants from other areas by incorporating the previously identified population cluster information with *a priori* designation of the migration rate (MIGPRIOR  = 0.05). All other parameters and run times followed previous population clustering runs (as detailed above in the preceding methods section on analyzing population genetic structure). We detected no biases in *a priori* assignment of the migration rate as the selection of particular MIGPRIOR values (0.001 to 0.1) did not substantially influence the STRUCTURE output, therefore only results for MIGPRIOR  = 0.05 are presented here.

Next, we used the likelihood-based estimator *L_h_/L_max_* in combination with the resampling algorithm of Paetkau *et al.* [94], implemented in the ‘detect migrants' function in GENECLASS to exclusively identify first generation migrants, i.e. individuals assigned to a different population other than the sampled population. The Paetkau *et al.* [94] routine was selected on basis of its superior simulation scheme which closely mimics natural processes and results in accurate type I error rates. *L_h_/L_max_*, is the ratio of *L_h_*, the likelihood of a given individual being assigned to its sampled population to *L_max_*, the greatest likelihood among all sampled populations [94]. We employed the Bayesian criterion of Rannala and Mountain [95] in combination with the re-sampling algorithm of Paetkau *et al.* [94] using a simulated set of 10,000 area-specific genotypes, to determine migrant thresholds (type I error *α* levels of 0.01 and 0.05) of *L_h_/L_max_*. We considered a minimum log likelihood *L_h_/L_max_* ratio of 2.0, which corresponds to a 100 times probability of being cross-assigned, as the threshold level for determining putative migrant status of an individual [7].

Third, the non-equilibrium Bayesian assignment test of BAYESASS was used to trace each individual's immigrant ancestry within the last two to three generations. Unlike STRUCTURE and GENECLASS analyses which require loci to follow Hardy-Weinberg and linkage equilibrium, BAYESASS is robust to violations from Hardy-Weinberg equilibrium as it measures contemporary gene flow within the last few generations based only on multilocus allele sharing among individuals [68]. To ensure convergence, a total run length of eight million MCMC iterations was performed, of which the first two million runs were the burn-in phase and the remaining six million runs comprised the data collection phase with the MCMC chain being sampled every 2,000 steps. Individual assignments and immigrant ancestries were calculated at a migration rate prior of 0.05, and keeping all other settings at default. Varying the prior rate (0.01 to 0.15) did not affect the results.

Lastly, to corroborate the results of the above assignment based migrant decisions, a likelihood based parentage analysis was carried out in CERVUS 3.0 [82], [96] to identify likely parent-offspring relationships between putative migrants and an individual in the cross-assigned source population based on log of the odds (LOD) scores. The LOD score that is the natural logarithm of the overall likelihood ratio for each candidate parent is calculated by multiplying together the likelihood ratios at each locus. A positive LOD score means that the candidate parent is likely to be the true parent, whereas a negative value means that the candidate parent is less likely to be the true parent. We estimated LOD scores for strict (95%) and relaxed (80%) confidence limits as 7.0 and 4.9 respectively, which were calculated from a simulated set of 10,000 offspring and 300 candidate parent genotypes, assuming 25% of candidate parents were sampled, 93% of loci were typed with typing error of 0.01 to 0.10.

Designation of migrant status to an individual was contingent upon - (i) significant assignment of first generation migrant status in GENECLASS (*P*<0.01, *Lh/Lmax ≥*2.0); (ii) observance of>50% migrant or cross-assignment probability in STRUCTURE and BAYESASS; (iii) high assignment probability to first generation immigrant ancestry state (gen1>50%) in both STRUCTURE and BAYESASS; and (iv) high membership (*Q*>0.8) to a single non-home cluster in the STRUCTURE analysis without prior population information. Further, in most cases of putative migrants, the successful parentage assignment corroborated the migrant status of the individual tiger. We considered a conservative approach by identifying individuals as putative migrants only if all three programs suggested evidence of immigrant ancestry.

### Estimation of Contemporary and Historical Migration Rates

In order to study gene flow across different timescales, we used the programs BAYESASS 1.3 [68] and MIGRATE 3.3.2 [97], [98] to compare migration rates over contemporary and historical timescales, respectively. Although the two programs use different approaches to derive estimates of gene flow, both programs generate parameters from which a comparative estimate of the proportion of genetic migrants in the population per generation (*m*) can be inferred. In BAYESASS, a Bayesian approach incorporating an MCMC sampling scheme is used to estimate migration rates between pairs of populations over the last few (approximately <5) generations back. With an estimated generation time of four to five years in tigers [99], this period corresponds to a timescale of nearly 20–25 years ago. MIGRATE on the other hand, uses the coalescent to estimate the relative mutation-scaled effective population size, theta, *θ_Ne_* (4*N_e_µ*; where *N_e_* is the effective population size and *µ* is the mutation rate) and asymmetric mutation-scaled immigration rate *M* (*m/µ*). The mutation-scaled immigration rate *M*, which is the immigration rate *m* divided by the mutation rate *µ*, is a measure of the importance of immigration events over mutation in contributing to variation in the population [97]. The relative effective population size, theta, is the number of individuals representing an idealized (Wright-Fisher) population that will result in the same amount of genetic drift as in the actual population. The number of migrants per generation, 4*N_e_m*, is the product of theta and *M*. MIGRATE assumes mutation-migration-drift equilibrium with values of *M* and theta constant over time and parameter estimates in MIGRATE date back nearly 4*Ne* generations into the past (approximately thousands of years ago). Hence, these migration rates provide estimates of gene flow that post and pre-date the estimated time (approximately 600 years ago) when humans began to significantly alter the habitats in which these tigers currently live.

For both the BAYESASS and MIGRATE runs, we used the STRUCTURE defined population clusters to estimate pairwise migration rates. BAYESASS runs were performed as described in the preceding methods section on detecting migrants. A total of 8×10^6^ MCMC iterations were carried out, by discarding the first 2×10^6^ steps as burn-in and sampling at every 2,000 iteration intervals of the remaining 6×10^6^ MCMC chain. Runs were carried out with a migration rate prior of 0.05 while other parameters were kept at default settings. The average result from three independent BAYESASS runs is presented. Estimates of historical gene flow and effective population size were carried out in MIGRATE by using the Bayesian approach and the Brownian motion model as an approximation for the step-wise microsatellite mutation model. Following initial trial runs, the Bayesian search criteria for the MCMC sampler was set at 10 replicates of one long chain of 50,000 steps with every 100 steps of the chain being recorded, producing a total of 5×10^7^ visited parameter values. The initial 10^7^ steps of the MCMC run were discarded as burn-in, and the remaining 4×10^7^ runs were sampled. To increase efficiency of the sampler, we used four chain-heating temperatures of 1, 1.5, 3 and 10,000, which allows a more efficient exploration of the genealogy space. We used parameter estimates from the initial run to calculate starting values of theta, for use as new parameters during subsequent runs. Parameter estimates from the final run were similar to the results of the initial runs. All MIGRATE runs were carried out on the Bioportal cluster computing facility at the University of Oslo, Norway (https://www.bioportal.uio.no/; accessed 12 May 2013).

### Detection of Genetic Bottleneck

To detect past occurrences of genetic bottleneck in the sampled populations, we evaluated three summary statistics - (i) Wilcoxon's sign rank test and (ii) mode-shift test, implemented in the program BOTTLENECK 1.2.02 [100], and (iii) *M* ratio test [101] implemented in ARLEQUIN 3.1 [102]. We were not interested in quantifying population expansion/decline or dating the time of and therefore avoided using other considerably lengthy Bayesian procedures [103], [104]. Wilcoxon's test detects bottlenecks based on the probability of heterozygosity excess over that expected at mutation-drift equilibrium in a population. It is most effective at detecting historic bottlenecks occurring over approximately 2–4 *N_e_* generations in the past. The mode-shift test is more suited for detecting bottlenecks within the last few dozen generations [105], [106]. This test is based on the premise that a stable population at mutation-drift equilibrium will have a large proportion of alleles at low frequency and a smaller proportion at intermediate frequencies and few at high frequencies. The resulting allele proportions yield an L-shaped distribution. In bottlenecked populations the mode is shifted because of the rapid loss of alleles present at low frequency. We ran 10,000 simulations using the program BOTTLENECK in 5 populations (excluding KPC, ATR and TATR) under both the stepwise mutation model (SMM) and the two-phase mutation model (TPM) with 95% single step mutations and 5% multi-step mutations and a variance of 12 as recommended by Piry *et al.* [100]. *P*-values from the Wilcoxon's test were used to examine bottlenecks at each timescale and were assessed at the alpha 0.05 level. The *M* ratio (*M* = *k*/*r*+1) was calculated from the mean ratio of the number of alleles (*k*) and the allele size range (*r*). Assuming loci follow a generalized stepwise mutation model, the loss of rare alleles would diminish the value of *k* at a faster rate than *r* thereby a drop in the *M* ratio below a threshold of 0.68 would be suggestive of populations that experienced a recent bottleneck [101].

### Field Data Collection for Occupancy Analysis

#### (A) Tiger Sign Surveys

The entire study area was divided into 10×10 km grids. Each grid that contained potential tiger habitat (all types of forest cover) was surveyed by replicate search paths for tiger sign. The number of surveys per grid ranged from 3 to 35, and was proportional to the amount of tiger habitat within each grid. Areas under agriculture, industry, and human habitation that were known to be non-habitat for tigers were not surveyed in an occupancy framework. Each survey consisted of a 5 km search for tiger signs with approximately one survey for every five km^2^ of habitat. Surveys were not random, but instead conducted along features that were likely to have tiger sign (e.g. dirt roads, dry water courses, and animal trails) so as to maximize detections [22]. Surveys were conducted by the local guard and a local assistant who had intimate knowledge of the forest and were trained to observe and record tiger sign in pre-designed datasheets. All encounters of tiger pugmark track sets and scats were recorded. These were distinguished from those of other carnivores based on criteria described by Jhala *et al.* [107] and Karanth and Nichols [108]. A total of 79,000 km of search effort was invested in 15,800 replicate surveys between December 2009-February 2010 (cold and dry season) across the entire study landscape to adhere with the assumption of occupancy closure [70] and have minimal influence of weather (rainfall) on sign detections and distribution. A total of 1,851 grids were sampled.

#### (B) Prey Assessment

Within each forest beat, one or two permanent line transects of two to four km in length were delineated. Each transect was walked on two or three subsequent mornings (06:30 to 08:30 hrs) by two observers to record encounter rates of wild ungulates and domestic livestock. Data on number of each species seen and the length of transect were recorded to compute encounter rates of each species. In disturbed forests (outside of protected areas) wild ungulate densities were low, animals were shy, and difficult to record using line transects. Therefore, at every 400 m along the line transect we also sampled a plot of 20×2 m to record ungulate dung. Dung was visually distinguished to species [107] and dung density for each species, wild ungulates as a group, and domestic livestock was computed separately. Encounter rates of ungulates and dung density were used as indices of ungulate abundance. The number of transects within each 100 km^2^ grid ranged from 1 to 24, and were proportional to the quantum of tiger habitat within that grid. The total effort invested in transect surveys was 71,468 km of walking on 26,688 occasions.

#### (C) Human Disturbance

At every 400 m along transects established for ungulate assessment a plot of 15 m radius was sampled to assess indices of human impact. Presence of (a) human/livestock trails within the plot, and (b) sighting of humans and livestock from the plot were recorded [107]. The number of plots within a 100 km^2^ grid ranged from 5 to 120. The total number of plots sampled across the landscape was 51,073.

### Remotely Sensed Variables

Remotely sensed data that depict landscape characteristics and human impacts were obtained from various sources and extracted at the 10×10 km grid resolution. Forest cover was obtained from the Forest Survey of India [109] that is based on IRS 1D LISS III satellite with 4 multispectral band data at 23.5 m resolution. Normalized Differential Vegetation Index (NDVI) information were derived from 1 km^2^ Advanced Very High Resolution Radiometer (AVHRR) data, acquired from the National Aeronautics and Space Administration's (NASA) Television Infrared Observation Satellite (TIROS) (http://science.nasa.gov/missions/tiros/; accessed 23 Dec 2010). Road and drainage information were obtained from Digital Chart of the World (http://statisk.umb.no/ikf/gis/dcw/; accessed 20 Dec 2010). Protected area shape files were obtained from the wildlife database at the Wildlife Institute of India, National Tiger Conservation Authority and State Forest Departments of India. The Shuttle Radar Topography Mission has produced the most complete, high-resolution digital elevation model of the earth [110]. Within each 1 km^2^ grid, this information was used for computing average elevation and the coefficient of variation of elevation used as a measure of terrain ruggedness. Night light data was obtained from the United States Air Force Defense Meteorological Satellite Program (DMSP) and National Oceanic and Atmospheric Administration's (NOAA) Operational Linescan System (OLS) (http://www.ngdc.noaa.gov/dmsp/sensors/ols.html; accessed 18 Dec 2010). Density of roads (length of paved road per km^2^), and Euclidean distances to roads, protected areas and night lights were computed in ArcGIS 9.3 (www.esri.com) software.

### Occupancy Modeling

Though sampling was done at the level of the forest beat (an administrative unit of about 15 km^2^) so as to ensure an even distribution of sampling effort across the landscape, analysis was done at the scale of 1,851 grids, each of size 10×10 km. This grid size was chosen since it was larger than the average home range size of a tiger [111], [112] and the size was relevant for subsequent administrative and managerial inputs. Sign surveys of 5 km independent spatial replicates within each grid [113] were modeled to address the issue of imperfect detections of tiger sign using the program PRESENCE v6.3 [114]. Detection of tiger signs was likely to depend on the abundance of tigers within a grid [115]. We first modeled the detection process by (i) keeping detection (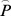) constant across surveys, (ii) 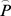 varying across surveys and (iii) 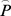 as a function of tiger abundance in that grid, wherein we used average encounter rate of tiger sign as an index that surrogated tiger abundance [31], [115]. The model that best explained the detection process based on Akaike Information Criteria (AIC) was then used in all subsequent models of tiger occupancy [70].

Tiger site occupancy was *a priori* expected to be positively influenced by (a) prey abundance, and (b) amount and quality of tiger habitat, and negatively influenced by (c) human disturbance [22], [115], [116]. We tested these hypotheses by modeling variables representing these factors as covariates using the logit link function in PRESENCE [70], [114]. We initially generated data on 23 site covariates that represented landscape and habitat features (forest area, core forest area, forest patch size, NDVI, elevation, ruggedness, drainage density, rainfall, distance to protected area), prey availability (chital, sambar, wild pig and gaur encounter rates on line transect walks, and wild ungulate dung density), human disturbance (distance to night lights, distance to roads, humans and livestock encountered on transect walks, human/livestock trails within sampled plots, and livestock dung density) that could potentially explain tiger occupancy. These covariates were examined with exploratory data analyses for their interrelationships and relationship to tiger presence (by scatter plots, box plots, and correlation analysis) and based on this a subset of 16 variables was selected for inclusion as site covariates for occupancy modeling (see Table S1 for univariate coefficients). Since many of the covariates had high correlation coefficients between them, their contribution to explaining tiger occupancy would be redundant. To account for this collinearity and to reduce the dimensionality of the covariate matrix we extracted Principal Components (PCs) from 16 variables [117]. The varimax rotated PCs were further used as independent variables in a logit link function to model tiger occupancy in the program PRESENCE ([70], available for download from http://www.proteus.co.nz/). Model selection was done using AIC, and model fit was assessed by comparing the actual detection histories with simulations generated from 50,000 parametric bootstrap runs of the target model in PRESENCE. The over dispersion parameter Ĉ close to one suggests that the model provides an adequate description of the data, values of Ĉ greater than one suggests more variation in the data than expected by the model, and values less than one suggest less variation in the data. The standard errors of model estimates were adjusted by the square root of Ĉ as recommended by MacKenzie *et al.* [70]. Models were built using PCs that represented prey abundance, human disturbance and habitat quality; these were evaluated against the null model and the full model by their delta AIC values. A total of six models were evaluated for modeling tiger occupancy and coefficient estimates for all models with delta AIC <4 were averaged based on model weights [70] to arrive at occupancy probability (*Ψ*) in each grid.

### Tiger Population Extents and Occupied Habitats

We used two approaches to estimate population extents and area of occupied habitats; (i) a more conservative approach wherein we considered only those grids that detected tiger sign as being occupied (the naïve estimate) and (ii) model inferred occupancy that corrected for detection bias and covariates in PRESENCE. Herein, landscape scale occupancy was computed by sum of cell occupancy probability values and divided by the total number of cells. Tiger habitat (forested area) in each grid was weighted by the tiger occupancy probability of that grid and summed across all grids to arrive at occupied tiger habitat for the landscape [115]. All adjacent tiger sign detected cells were joined and were considered to be occupied by a single tiger population.

### Habitat Corridor Modeling

Grid based tiger occupancy probability (*Ψ*) obtained from PRESENCE was used as a measure of habitat suitability for tigers [69], [70]. A cost surface for tiger habitat suitability across grids was generated as 1-*Ψ*. For the non-tiger habitat (human land uses) where tiger occupancy values were not available, we considered them permeable to tigers at higher costs than forested habitats, although human habitations (townships) were considered impermeable to tigers (see Methods S2 for details). These costs were used as a resistance layer for modeling habitat connectivity using LCP [63] and circuit theory [118] analyses. Based on these cost surfaces the resulting models would optimize connectivity by selecting high quality tiger habitat and minimize gaps formed by non-tiger habitat. Least-cost pathways (LCP) were modeled using PATHMATRIX [71], and resistance pathways were modeled using CIRCUITSCAPE [72]. Core areas of tiger reserves were considered as “sources” or areas of high potential from which tiger movement across paths of least resistance were modeled across the landscape. PATHMATRIX models several potential routes in a radiating manner from the “source” to connect to another adjacent “source”. It then selects a single “least cost” pathway as the best alternative. CIRCUITSCAPE models connectivity through habitat swaths considering resistance to movement based on pixel cost and corridor width [118]. It provides one to several potential alternatives for joining sources and helps in identifying bottlenecks within the corridors. Since the Central Indian landscape is highly fragmented and human dominated with clearly defined and limited forested habitat, we could overlay LCP on high resolution Google Earth images and align them to match geographic features within occupancy grids, to delineate realistic corridors. These least cost corridors buffered by 1.5 km (LCC) were considered the minimal essential corridors joining two tiger reserves.

### Genetic Structure, Migrants and Corridor Cost

The genetic structure between populations is a consequence of the amount of genetic drift to which each local population has been subjected, due to its local effective size, and/or due to its degree of demographic, geographic and ecological isolation [119]. Since all the Central Indian tigers likely belonged to a large, mostly contiguous population till a few hundred years ago [23], pairwise genetic distances between populations should reflect levels of differentiation and barriers to gene flow, i.e., the cost of movement between these populations. The proportion of migrants between population pairs would reflect gene flow in current or recent times while *F*_ST_ values would indicate genetic differences over historical, more long-term time scales. We therefore expected to have more migrants detected between geographically closer populations that had lower movement costs between them. This exploratory analysis would corroborate the short-term mechanisms (migration events) that result in long-term (*F*_ST_) genetic differences between populations due to tiger movement across the landscape.

Pairwise *F*_ST_, *R*_ST_ and *Phi*_PT_ genetic distance estimates, obtained from AMOVA analysis in GENALEX, were linearized using the formula *F*_ST_/(1 - *F*_ST_), as given by Rousset [120]. In order to determine which spatial model best explained genetic structuring, matrices of linearized pairwise genetic distances were correlated against matrices representing geographic distances (GGD), log-transformed geographic distances (log_10_ GGD), least-cost pathway distances (LCPD) least-cost corridor distances (LCCD), and resistance distances (RD). In addition, the effectiveness of the modeled corridors and spatial distance matrices was compared using partial correlations that allowed one model to be tested, while controlling for other competing models [55]. The biologically realistic model not only exhibited the highest significant, positive correlation, but also displayed significant positive partial correlations after controlling for each of the competing models. Mantel [121] and partial Mantel [122] tests were carried out with 10,000 randomizations in the program zt [123] to evaluate the significance of the correlations.

As patterns of isolation-by-distance (IBD) are known to bias tests of hierarchical structuring and vice-versa, we used the population clusters as a covariate in a partial Mantel test, to model the partial correlations between pairwise genetic distances and spatial distances, while controlling the effect of population clusters (following Meirmans [124]). Partial correlations between matrices representing pairwise genetic distances and spatial distance matrices were calculated with a third matrix describing whether population comparisons were made between (1) or within (0) the STRUCTURE identified clusters. A non-significant or negative partial correlation of genetic with geographic distance, after controlling for population clusters, would rule out patterns of underlying IBD in the observed genetic structure.

## Results

### Identified Individuals and Descriptive Statistics

Out of 587 scat samples, 330 scats were successfully PCR amplified using the felid specific mitochondrial DNA cytochrome *b* (mtDNA cyt *b*) primers, of which 275 were identified as tiger scat based on *Bam*HI restriction enzyme digestion profile of the PCR products (Table 1 and Figure S1). From the 275 tiger scats, only 250 samples yielded microsatellite genotype data at a minimum of seven loci to be considered for *Identity* analysis in CERVUS. We identified 169 individuals with 81 recaptures from a total of 267 microsatellite loci genotypes (250 scats, 16 blood and 1 tissue). The total number of individuals identified here constitutes roughly 49% of the estimated total tiger population in the entire sampled area (Table 1). Sex identification yielded 74 males and 85 females, with the sex ratio being nearly symmetric in most localities, except in the lower sampled areas. On average, individual multilocus genotypes were 93.2% complete. We included 97% (164 out of 169) of samples, those that had complete or near complete genotypes with a maximum of two missing loci, for further analyses. Two Tadoba individuals with three missing loci (73% complete) and three individuals from the Kanha Pench corridor with four missing loci (64% complete) were also retained since sample size from these areas was small and the genotypes represented unique tigers. The panel of eleven microsatellite loci that was used for individual discrimination had very low cumulative sibling probability of identity (*PI*-*sib*) of 1.5×10^−4^ (Table S1), indicating very high power to discriminate individuals. Even in the samples with the least amount of genotype information (four missing loci), the cumulative *PI-sib* value (1.6×10^−3^) of the samples was sufficiently low to determine unique individuals.

No significant evidence of linkage disequilibrium (LD) among all pair-wise loci combinations was observed, when all sampling locations were pooled (*p*>0.05 at 1,000 permutations). Except for deviation at three loci, Fca441, Pati09 and Pati18 (*p*<0.001), all other loci were in Hardy-Weinberg equilibrium (HWE) in the pooled population after adjusting the critical *P*-value using the Bonferroni correction procedure (Table S1). Loci not in HWE or with null allele frequencies>5% appear to be random with respect to population (Table S2). Pati09 showed significant deviation from HWE across three populations, while Pati01 and Fca441 showed significant deviation from HWE in two populations. 6Hdz700, F53 and Fca304 each deviated from HWE in one population. Such departures from HWE could indicate the possibility of genetic structuring among populations and likely presence of related individuals in the data. The deviation from HWE in few populations could also be explained by the presence of null alleles in the data. While MICROCHECKER tests did not show any evidence of scoring errors due to stuttering or small allele dominance in the dataset, five loci (Pati01, Pati15, Pati18, Fca304 and F53) with high null allele frequencies>9% were detected (Tables S1 and S2). Three loci deviating from HWE were likely due to the presence of null alleles in the data - Fca304 in Bandhavgarh, and Pati01 and F53 in the Melghat-Satpura-Tadoba cluster, as they showed significant deviation from HWE only with observed allele frequencies and not with null allele corrected frequencies (not shown). Although Pati15 and Pati18 also contained null alleles at high frequencies (>9%) in Kanha, they did not deviate from HWE in any population. Since these patterns were random with respect to population (e.g., no departures from HWE in Kanha, and null allele frequency <5% in Pench), it was more likely that these deviations could be due to the presence of population structuring in the data. Hence we retained all eleven loci in subsequent analyses.

Most tigers genotyped in this study showed high heterozygosity (Figure S2). The minimum number of heterozygotes observed across loci for an individual tiger's multilocus genotype was two and three heterozygous loci, for one and two tigers respectively. The most polymorphic individuals (n = 5) were heterozygous at all eleven loci. Individuals that were completely homozygous at all eleven loci, were not observed. Nearly 90% of the tigers (i.e. 152 out of 169 individuals) had heterozygous genotypes at five to nine loci, and 12 tigers were heterozygous at ten to eleven loci. Genetic diversity estimates showed a mean number of alleles per locus to be 9.1±2.2 with heterozygosity *He* to be 75.4%±3.9 and *Ho* to be 70.1%±5.9 in the pooled population (Table 2). A summary of population-wise genetic variation, revealed significantly higher allelic diversity and heterozygosity levels in the larger populations of Pench and Kanha compared to all other localities (Table 2 and Table S2). The number of population diagnostic private alleles was high in Pench (n = 8) and Kanha (n = 6), while individual frequency for a private allele was highest in Bandhavgarh compared to all other localities (Table S3). Estimated population size was positively and linearly correlated to genetic diversity statistics (Number of alleles, *rho*  = 0.857, *p* = 0.011; polymorphism information content, *rho*  = 0.738, *p* = 0.0458; Shannon's index of diversity, *r* = 0.714, *p* = 0.058). Partial correlations between estimated population size and the allele diversity indices, after controlling for the effect of sample size, were positive and highly significant (Number of alleles, *r* = 0.82, *p* = 0.012; polymorphism information content, *r* = 0.659, *p* = 0.054; Shannon's index of diversity, *r* = 0.714, *p* = 0.036). Heterozygosity values were not correlated with population size (*Ho*: *rho*  = −262, *p* = 0.428; *He*: *rho*  = 0.357, *p* = 0.389). Correlation of estimated population size with actual sampled size was highly significant (*r* = 0.964, *p* = 0.001), meaning that our samples were in proportion to the size of the population.

**Table 2:** Summary statistics of mean genetic variation and bottleneck tests across sampled populations.

Population	Observed heterozygosity (St. Dev.)	Expected heterozygosity (St. Dev.)	Number of alleles (St. Dev.)	Allelic size range (St. Dev.)	*M* ratio (St. Dev.)	Wilcoxon's heterozygosity excess test	Mode-shift test
M	0.654 (0.234)	0.674 (0.110)	4.7 (0.6)	17.5 (4.3)	0.75 (0.21)	ns	L-shaped
S	0.673 (0.230)	0.639 (0.146)	4.2 (0.9)	16.7 (4.6)	0.70 (0.23)	ns	L-shaped
T	0.705 (0.195)	0.704 (0.094)	4.9 (1.5)	16.5 (5.9)	0.80 (0.15)	ns	L-shaped
M-S-T	0.691 (0.141)	0.774 (0.036)	7.6 (1.6)	24.9 (7.6)	0.86 (0.16)	ns	L-shaped
P	0.750 (0.070)	0.734 (0.056)	7.6 (1.5)	26.8 (11.5)	0.83 (0.18)	ns	L-shaped
K	0.685 (0.061)	0.674 (0.106)	7.2 (1.9)	22.5 (5.2)	0.86 (0.13)	ns	L-shaped
K-A	0.683 (0.085)	0.713 (0.051)	7.9 (1.6)	26.5 (10.6)	0.86 (0.20)	ns	L-shaped
B	0.641 (0.216)	0.607 (0.148)	4.5 (1.0)	18.6 (6.2)	0.66 (0.20)	ns	L-shaped
All	0.701 (0.059)	0.754 (0.039)	9.1 (2.2)	28.8 (10.5)	0.90 (0.14)	ns	L-shaped

### Population Genetic Structure

**Figure 2:**
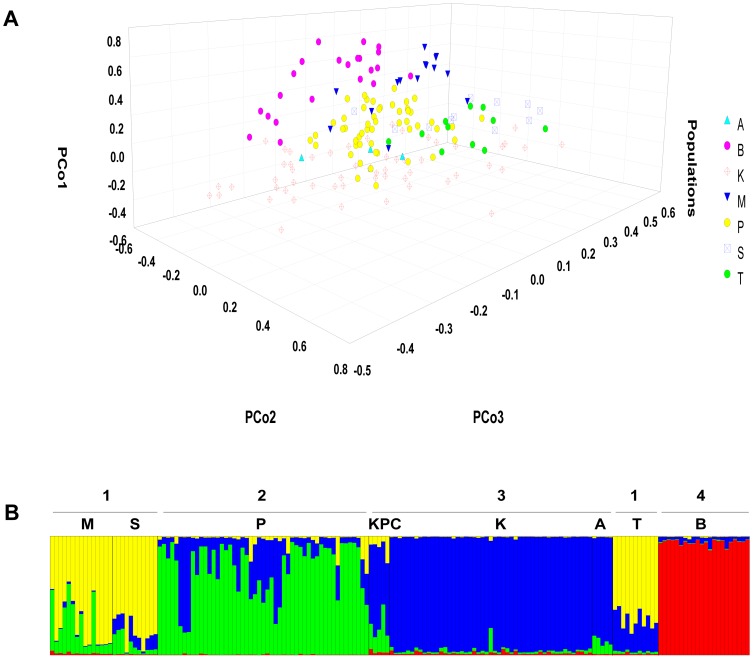
Results of individual clustering analyses. (**A**) Three dimensional plot showing partitioning between different populations as obtained from PCo analysis based on *Phi*_PT_ co-dominant genetic distance among individuals. (**B**) Summary barplot of STRUCTURE run at *K* = 4 showing population assignments for each individual. Four distinct population clusters are observed. Sampled populations are Melghat (M), Satpura (S), Pench (P), Kanha-Pench corridor (KPC), Kanha (K), Achanakmar (A), Tadoba (T) and Bandhavgarh (B).

According to the results of the Principal Coordinates Analysis (PCoA) based on *Phi*_PT_ genetic distance estimator, individuals in the area were clustered into roughly four groups with varying degrees of population partitioning (Figure 2A). The three coordinate axes accounted for 60% of the variation in the dataset. Tigers were observed to cluster in four major groups. Tigers from Kanha, Pench and Melghat formed three distinct clusters that partially overlapped each other, while Bandhavgarh tigers formed a discrete cluster with minimal overlap. Tigers from Satpura, Tadoba, and Achanakmar were scattered within the clusters formed by Kanha-Pench-Melghat.

Calculation of delta *K* from the output of the STRUCTURE runs using prior population information (locprior  = 1), produced the largest modal value of the statistic at *K* = 4, suggesting pronounced population subdivision at *K* = 4 (Figure S3). On the other hand, the log-likelihood *L(K)* value reached an inflection point at *K* = 4 before gradually plateauing at *K* = 6 to 7 and finally leveling off at *K* = 8 (Figure S3). The variance in *L(K)* increased at higher values of *K*, as reported previously with other studies [92], [125]. The disparity in population structuring patterns between delta *K* and log-likelihood values occasionally occurs in cases where the *F*_ST_ values are significant [126]. Examination of individual *Q* summary barplots (Figure S3) yielded identical clustering patterns at all runs between assumed *K* = 2 to 4 (carried out both with and without prior sampling location information), and distinct population saturation, indicative of population subdivision was evident at *K* = 4 in conformance with the delta *K* approach. Based on the above, the four cluster solution (*K* = 4) best describes the levels of genetic subdivision in our sample of the Central Indian tiger population (Figure 2B).

The four population clusters identified by STRUCTURE were similar to the clusters observed using PCoA plots, and in consonance with geographic configuration as well (Figure 1). Melghat, Satpura and Tadoba represented populations with patchy connectivity and formed a unique cluster in the western and southern limits of the landscape, with few individuals being cross-assigned to the Pench and Kanha clusters. To the east of Melghat and Satpura, Pench formed a unique cluster. A few individuals in Pench had cross-assignments with Kanha suggesting gene flow between these two population clusters substantiated by a functional habitat corridor between Pench and Kanha. The next cluster in the eastern part of the landscape was represented by tiger populations in Kanha and Achanakmar. Individuals from the forested corridor between Pench and Kanha were also assigned to this cluster, but most had mixed assignments to both populations indicating that this was not a distinct but rather an admixed population. The last cluster was represented by Bandhavgarh, in the north-eastern part of the landscape, which formed a distinct isolated population where all individuals were assigned to the sampled locality.

Partitioning of genetic variation in the AMOVA test indicated low but significant differentiation (*p*<0.01) between the STRUCTURE identified clusters (Table S4). The major portion of genetic variance was found within populations (88%) with 7% among the population clusters and 5% among populations within clusters. Exact tests showed significant genetic variance on all three levels (*p*<0.01). Both *F*_ST_ and *Phi*_PT_ values showed highly significant structuring (*p*≤0.001), and had relatively similar trends in magnitude with low sampling variance. In contrast, *R*_ST_ estimates showed no variation between groups, and had unreliably high sampling variances and mean square error estimates. The results of pair-wise *F*_ST_ and *R*_ST_ calculations indicated significant (*p*<0.05) and varied (*F*_ST_ 0.049 to 0.241; *R*_ST_ 0.000 to 0.330) genetic structuring between all sampled populations in Central India (Table S5). Within cluster *F*_ST_ estimates were mostly lower, ranging from moderately low (0.048 to 0.062) to high (0.079 to 0.102), compared to pairwise estimates between different clusters (0.127 to 0.217).

### Migrants

#### (A) STRUCTURE results

Eight putative migrants were identified in STRUCTURE (Table 3). Of these, four individuals (D954, D955, D958, D1399) were identified with>80% migrant and cross-assignment probability to a single non-home cluster in the STRUCTURE analysis, carried out without prior population information. The remaining four samples represented individuals (D1843, D1892, D1297, D2058) that had weaker migrant probability (*P*>0.5 to <0.7) and showed variable *Q* (0.289 to 0.758), and with the majority of samples having cluster memberships to more than one non-home locality.

**Table 3:** Summary of migrant assignments based on STRUCTURE, GENECLASS (migrants based on **α_0.01_ and *α_0.05_ type I error levels) and BAYESASS analyses.

tiger ID	sex	sampled locality	GENECLASS assigned population	GENECLASS *Lh/Lmax*	STRUCTURE *Q* assignment clusters (MST/P/KA/B; no prior population information, K = 4)	STRUCTURE migrant probability (gen1, gen2)	BAYESASS assigned population	BAYESASS migrant probability (gen1, gen2)	CERVUS assigned parent/population/pair LOD score	Final migrant status
*D955*	♂	*PTR*	*KTR*	3.234**	*0.035/0.009/0.889/0.067*	*0.996 (0.978, 0.018)*	*KTR*	*0.999 (0.990, 0.009)*	*D1182/KTR/-0.360*	Migrant
*D958*	♀	*PTR*	*KTR*	2.959**	*0.033/0.016/0.936/0.015*	*0.982 (0.964, 0.018)*	*KTR*	*0.997 (0.987, 0.010)*	*D1205/KTR/5.320*	Migrant
*D954*	♂	*PTR*	*KTR*	3.883**	*0.009/0.019/0.963/0.009*	*0.863 (0.774, 0.089)*	*KTR*	*0.962 (0.825, 0.137)*	*D402/KTR/0.760*	Migrant
*D1399*	♂	*PTR*	*KTR*	3.222**	*0.087/0.022/0.885/0.007*	*0.868 (0.552, 0.316)*	*KTR*	*0.998 (0.702, 0.296)*	*NE*	Migrant
*D1843*	♂	*MTR*	*PTR*	2.343**	*0.275/0.527/0.186/0.013*	*0.590 (0.502, 0.088)*	*PTR*	*0.986 (0.842, 0.144)*	*D1381/PTR/4.210*	Migrant
*D1297*	♀	*KPC*	*PTR*	6.444**	*0.075/0.683/0.215/0.026*	*0.610 (0.130, 0.480)*	*PTR*	*0.559 (0.124, 0.436)*	*D1043/PTR/2.490*	Admixed
D1987†	♂	STR	PTR	2.407**	0.481/0.325/0.180/0.014	0.311 (0.005, 0.306)	PTR	0.550 (0.006, 0.544)	D2057/MTR/0.300	Admixed
D525	♂	TATR	KTR	2.562**	0.664/0.023/0.300/0.013	0.170 (0.031, 0.139)	KTR	0.617 (0.086, 0.531)	NE	Admixed
D2154	♀	TATR	PTR	<2.0*	0.460/0.158/0.358/0.024	0.007 (0.001, 0.006)	TATR	0.188 (0.056, 0.132)	NE	Admixed
D1892	♂	MTR	PTR	<2.0*	0.658/0.289/0.042/0.011	0.584 (0.113, 0.471)	PTR	0.996 (0.011, 0.985)	D578/PTR/0.070	Admixed
D2058	♀	MTR	MTR	0	0.147/0.758/0.019/0.076	0.508 (0.173, 0.335)	PTR	0.895 (0.036, 0.859)	NE	Admixed
D1140	♀	KTR	PTR	<2.0*	0.070/0.556/0.344/0.030	0.124 (0.016, 0.108)	PTR	0.241 (0.035, 0.206)	D1043/PTR/3.540	Admixed
D1075	♂	PTR	KTR	<2.0*	0.065/0.237/0.663/0.034	0.096 (0.012, 0.084)	KTR	0.509 (0.161, 0.348)	D1926/STR/2.473	Admixed
D1381	♂	PTR	KTR	<2.0*	0.213/0.466/0.316/0.004	0.190 (0.000, 0.190)	KTR	0.832 (0.012, 0.820)	D1185/KTR/0.351	Admixed
D1383	♂	PTR	PTR	0	0.068/0.510/0.420/0.003	0.159 (0.001, 0.158)	KTR	0.926 (0.009, 0.917)	D1114/KTR/4.471	Admixed
D1393	♂	PTR	KTR	<2.0*	0.089/0.083/0.807/0.021	0.188 (0.013, 0.175)	KTR	0.872 (0.264, 0.608)	D578/PTR/1.066	Admixed
D1400	♂	PTR	PTR	0	0.146/0.110/0.738/0.006	0.229 (0.015, 0.214)	KTR	0.829 (0.201, 0.628)	D1168/KTR/-2.155	Admixed

*Q* assignments depict individual membership to each of the four STRUCTURE identified population clusters of Melghat-Satpura-Tadoba (MST), Pench (P), Kanha-Achanakmar (KA), and Bandhavgarh (B). Migrant probability refers to the *P* value of each individual to its cross- assigned population. The assignment likelihood of each migrant to a likely candidate parent and its source population is shown by CERVUS log (LOD) scores. Italicized individuals depict potential migrants identified unanimously by all three programs. Localities depicted include Melghat (MTR), Satpura (STR), Pench (PTR), Kanha-Pench corridor (KPC), Kanha (KTR) and Tadoba (TATR).

† Tigers reported dead during the study period, M-Male, F-Female, NE- not established.

#### (B) GENECLASS results

GENECLASS also identified eight individuals (Table 3) as putative migrants (*P*<0.01), with high log likelihood of cross-assignment (*L_h_/L_max_*>2.0). Lowering the likelihood threshold (*L_h_/L_max_*<2.0) yielded further six putative migrants (*P*<0.05).

#### (C) BAYESASS results

A total of fifteen individuals with likely immigrant or admixed ancestry were detected in the BAYESASS analysis (Table 3). Of the fifteen total migrants, eleven individuals had high migrant cross-assignment probabilities (*P*>0.8), and four individuals had intermediate migrant assignments (*P* = 0.509 to 0.617). In general, the posterior probabilities of migrant assignment were higher in BAYESASS compared to the STRUCTURE analysis. Additionally, BAYESASS identified six other individuals (D1075, D1381, D1383, D1393, D1400, D1987) as potential migrants (*P* = 0.509 to 0.926) that were not assigned as migrants by either STRUCTURE or GENECLASS.

#### (D) CERVUS results

Identification of offspring-candidate parent pairs in CERVUS yielded parentage relationships in thirteen out of seventeen putative migrants (Table 3). No evidence of likely parentage (or sibling) relationships in the offspring population was observed. The cross-assigned population in ten of the thirteen individuals matched the parentage assignment in CERVUS, which serves to further corroborate the results of the migrant assignments. Only three mismatches (D1075, D1393 and D1987) were observed between the parent populations identified by CERVUS and the population assignments depicted by the migrant analysis, but this could be due to low information in the data as opposed to incorrect migrant assignment. Except for negative LOD value in two pairs (D955–D1182, LOD = −0.36; D1400–D1168, LOD = −2.15), LOD scores were positive in the remaining eleven putative parent-offspring pairs. The relationship between a potential migrant sampled in Pench (D958) with a candidate parent from Kanha (D1205) was identified with>80% confidence in assignment (LOD = 5.32). LOD scores in remaining offspring-parent pairs were below the 80% confidence limit (<4.9).

**Figure 3:**
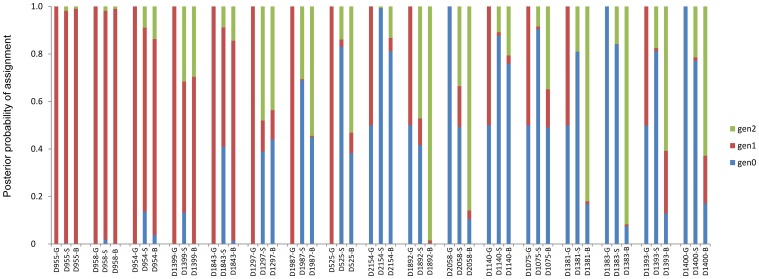
Individual ancestry states of putative migrants. Posterior distributions of individual assignment to nonimmigrant (gen0), and first (gen1) and second generation immigrant (gen2) ancestry states. Suffixes after indvidual names indicate assignment probabilties as obtained in GENECLASS (G), STRUCTURE (S) and BAYESASS (B).

The detection of migrants by the above methods yielded a total of seventeen individuals with putative immigrant ancestry (Table 3 and Figure 3). Identical migrant assignment across all three programs was observed in seven individuals (D954, D955, D958, D1297, D1399, D1843, D1892), while there was equivocal assignment in the remaining ten individuals. Sex identification revealed 12 out of 75 males (16%) and 5 out of 84 females (6%) as individuals with immigrant ancestry in the entire area. Figure 3 shows the posterior distributions of individuals assigned to nonimmigrant (gen0), first (gen1) or second generation immigrant (gen2) ancestry states in GENECLASS, STRUCTURE and BAYESASS. All GENECLASS migrants with *L_h_/L_max_*>2.0 were classified as 100% first generation migrants. Two individuals (D955, D958) with>90% gen1 assignment and three individuals (D954, D1399, D1843) with relatively high gen1 assignment probability (0.5 to 0.7) were considered as migrants. Five individuals (D525, D1892, D2058, D1297 and D1987) showed moderate levels of migrant assignment and immigrant ancestry patterns are indicative of admixed status. The assignment status of seven more individuals (D1075, D1381, D1383, D1393, D1400, D1140 and D2154) was equivocal. While STRUCTURE could not assign them as migrants, they were identified as potential second generation migrants or admixed individuals in BAYESASS.

### Contemporary and Historical Migration Rates

The mean posterior distributions of pairwise immigration rates depicting contemporary gene flow estimates in BAYESASS are shown in Tables 4 and 5. Most populations have low migrant proportions with the exception of migration from Pench to Melghat (*m* = 0.09) and Kanha to Pench (*m* = 0.07) where the rates were more than 5% (Table 4). Gene flow between Melghat and Pench was likely asymmetric and there appears to be a source-sink relationship because the expected proportion of migrants into the Pench population from Melghat is much smaller (*m* = 0.015). Asymmetric migration was also visible between the Pench and Kanha population clusters, as the proportion of migrants from Pench to Kanha was negligible (*m* = 0.006). The Bandhavgarh population was devoid of migrants as suggested by the lack of gene flow between other populations (*m*<0.01). As in the locality-wise analysis, similar asymmetry and rates of migration were obtained between the various STRUCTURE defined population clusters (Table 5).

**Table 4:** Locality-wise contemporary migration rates, *m*, estimated using BAYESASS, showing means (± standard deviation) of the posterior distributions along with the 95% confidence intervals in parentheses.

	M	S	P	K	T	B
**M**	**0.856±0.042 (0.773, 0.937)**	0.014±0.015 (0.000, 0.054)	0.092±0.038 (0.026, 0.173)	0.015±0.016 (0.000, 0.057)	0.011±0.013 (0.000, 0.046)	0.012±0.014 (0.000, 0.051)
**S**	0.014±0.017 (0.000, 0.059)	**0.899±0.045 (0.805, 0.975)**	0.037±0.031 (0.000, 0.115)	0.026±0.026 (0.000, 0.099)	0.012±0.015 (0.000, 0.049)	0.012±0.015 (0.000, 0.054)
**P**	0.004±0.006 (0.000, 0.020)	0.025±0.014 (0.003, 0.059)	**0.897±0.029 (0.838, 0.952)**	**0.065±0.025 (0.022, 0.120)**	0.005±0.006 (0.000, 0.024)	0.003±0.004 (0.000, 0.016)
**K**	0.008±0.007 (0.000, 0.026)	0.003±0.004 (0.000, 0.014)	0.005±0.007 (0.000, 0.024)	**0.976±0.015 (0.940, 0.996)**	0.007±0.008 (0.000, 0.030)	0.002±0.004 (0.000, 0.014)
**T**	0.025±0.032 (0.000, 0.114)	0.012±0.015 (0.000, 0.054)	0.015±0.021 (0.000, 0.068)	0.021±0.029 (0.000, 0.102)	**0.917±0.068 (0.756, 0.998)**	0.010±0.014 (0.000, 0.048)
**B**	0.003±0.006 (0.000, 0.017)	0.003±0.006 (0.000, 0.020)	0.003±0.006 (0.000, 0.020)	0.003±0.006 (0.000, 0.019)	0.003±0.006 (0.000, 0.019)	**0.986±0.014 (0.952, 0.999)**

The populations into which individuals are migrating are listed in the rows, while the sources of the migrants are listed in the columns. Values along the diagonal are proportions of individuals derived from the source populations each generation. Migration rates ≥0.05 are in bold. Individuals from Achanakmar (*n* = 4) and the Kanha-Pench corridor (*n* = 5) were excluded due to low sample sizes, and only included in the cluster based test (provided in Table 5).

**Table 5:** Cluster-wise contemporary migration rates, *m*, showing means (± standard deviation) of the posterior distributions along with the 95% confidence intervals in parentheses.

	MST	P	K	B
**MST**	**0.915±0.027 (0.860, 0.962)**	**0.064±0.025 (0.020, 0.117)**	0.015±0.016 (0.000, 0.058)	0.006±0.007 (0.000, 0.026)
**P**	0.015±0.016 (0.000, 0.054)	**0.905±0.029 (0.846, 0.958)**	**0.076±0.026 (0.029, 0.129)**	0.004±0.005 (0.000, 0.019)
**K**	0.009±0.009 (0.000, 0.033)	0.006±0.008 (0.000, 0.029)	**0.981±0.014 (0.950, 0.999)**	0.003±0.004 (0.000, 0.017)
**B**	0.005±0.007 (0.000, 0.028)	0.005±0.007 (0.000, 0.027)	0.005±0.008 (0.000, 0.028)	**0.986±0.014 (0.950, 0.999)**

The population clusters into which individuals are migrating are listed in the rows, while the sources of the migrants are listed in the columns. Values along the diagonal are proportions of individuals derived from the source populations each generation. Migration rates ≥0.05 are in bold.

Results from the MIGRATE analysis showed low estimates of relative effective population size (theta, *θ*) and historical mutation scaled immigration rate (*M*) suggesting very low overall migrant proportions in the area over the long-term (Table 6). Theta estimates were low to moderate and ranged from 0.57 (Bandhavgarh), 0.77 (Kanha-Achanakmar, and Melghat-Satpura-Tadoba) to 1.5 (Pench). Estimates of *M* ranged from a high of 6.17 (Pench to Bandhavgarh) to a low of 0.9 (Bandhavgarh to Melghat-Satpura-Tadoba), revealing limited to no migration among populations in the landscape (Table 6). Of the twelve pairwise population comparisons, ten pairs showed asymmetric migration patterns, with the higher value of *M* representing immigration from the population with the larger theta value (Kanha and Pench clusters), to the population with the smaller theta value (Melghat-Satpura-Tadoba and Bandhavgarh clusters). The number of migrants per generation ranged from almost zero (Bandhavgarh to Melghat-Satpura-Tadoba cluster) to nine (Pench to Bandhavgarh). The Pench and Kanha clusters represented the largest source populations for immigrants in the area. Though marginally higher migration from Pench to Kanha was visible compared to migration in the other direction, both population clusters had overall symmetric gene flow.

**Table 6:** Means of posterior distributions of mutation scaled immigration rate, *M*, along with the 95% confidence limits (before comma) and mean number of migrants per generation (after comma) estimated from Bayesian runs in MIGRATE.

	MST	P	KA	B
**MST**	0.77 [0–1.93]	4.43 [0–7.2],	1.83 [0–3.87],	0.9 [0–2.67],
		6.6	1.4	0.5
**P**	1.43 [0–3.53],	1.5 [0.533–3.0]	1.83 [0–4.4],	2.83 [0–5.4],
	1.1		1.4	1.6
**KA**	2.97 [0–6.13],	2.17 [0–5.4],	0.77 [0–2.0]	2.43 [0–4.8],
	2.3	3.3		1.4
**B**	3.9 [0–19.4],	6.17 [0–25],	4.03 [0–21.8],	0.57 [0–1.53]
	3	9.3	3.1	

Population clusters listed in rows depict the populations into which individuals migrate, while the source populations of individuals are shown in the columns. The values along the diagonal are estimates of the relative effective population size, *θ*.

### Genetic Bottleneck

Except for Bandhvagarh, which had a low *M* ratio of 0.66, all other populations showed *M* values above the critical threshold of 0.68 (Table 2). Wilcoxon's sign rank test of heterozygosity excess were not significant (*p*>>0.05) regardless of the mutation models used and all localities tested showed normal L-shaped allele distributions in the mode-shift test, indicative of stable non-bottlenecked populations (Table 2). Only, the Pench and Kanha populations showed significant (*p*<0.05) evidence of heterozygosity deficiency (Table S6), which could suggest recent events of expansion in these two populations.

### Tiger Occupancy, Habitat, Prey and Human Disturbance

Ten Principal Components (PCs) had Eigen values greater than one and together explained 86.6% of the variability of the original variables (Table 7). The factor loadings of the components permitted unambiguous ecological interpretation of PCs (Table 7). The detection probability of tiger sign was 21.98% (SE 0.04). Detection of tigers was best explained by index of tiger abundance. The closest competing model for detection differed by a ΔAIC value of 1207 (Table 8). Tiger occupancy was best explained by a model that included PC1 to PC9 and all the covariates conformed to the *a priori* predictions of their influence on tiger occupancy. The best model differed from the full model by ΔAIC of 0.45 and from the null model by ΔAIC of 1682. Tiger occupancy in a grid was best explained by (a) PC1 that represented availability of large undisturbed good canopy forests, (b) PC2 and PC4 that represented grids that had low human and livestock disturbances, (c) PC3, PC6 and PC9 that represented high ungulate prey especially in the form of chital, sambar, wild pig and gaur, (d) PC5 representing lower elevation, (e) PC7 representing grids within or in the proximity of legally protected areas and (f) PC8 that gave greater loadings for grids with higher rainfall (Tables 7 and 8). The sign of the coefficients of the best model for each PC was the same when models were run using original variables in a univariate model (Table S7). The goodness-of-fit test for observed data against 50,000 model based bootstrap samples failed to show lack of model fit (χ^2^ *P* value  = 0.11), Ĉ statistic was estimated at 1.4 and standard errors shown in Table 9 are corrected for over-dispersion by a factor of 1.18. Out of the 185,100 km^2^ area of the sampled grids, 76,913 km^2^ was forested or potential tiger habitat. The naïve estimate of grid occupancy was 17.5% while the model-averaged estimate of occupancy was 20.87% giving a 3.4% increment over the naïve estimate. The naïve estimate of tiger occupied habitat was 19,845 km^2^ while the model inferred occupancy of forests was 21,290 km^2^ giving an increment of 1.8% in occupancy estimate. The habitat variables of forest area, forest core area, rainfall, NDVI, and terrain ruggedness had significantly higher values in tiger occupied grids compared to unoccupied cells. All wild tiger prey indices were higher, while livestock abundance indices were lower in tiger occupied grids. Relevant human impact indices had significantly lower values in tiger occupied grids (Table 9 and Figure S4).

**Table 7:** Principal component (PC) loadings of covariates relevant for modeling tiger occupancy, Eigen values of the components, the percent variation of the original data explained by the PC, their ecological interpretation and *a priori* effect on tiger occupancy.

Covariates	PC1 (Forest)	PC2 (Human-Livestock)	PC3 (Chital, Sambar, Dung)	PC4 (Livestock Dung)	PC5 (Elevation)	PC6 (Gaur-Sambar)	PC7 (Legal Protection)	PC8 (Rainfall)	PC9 (Wild Pig)	PC10 (Distance to Urbanization)
**Elevation (m)**	−0.109	0.035	0.019	−0.032	0.962	−0.021	0.004	−0.059	0.042	0.116
**Rainfall (mm)**	−0.276	−0.033	0.009	0.005	−0.079	−0.048	−0.027	0.871	0.098	0.180
**Mean NDVI**	−0.795	0.017	0.037	−0.010	0.035	0.102	−0.002	0.195	0.037	−0.017
**Forest Area of Grid**	−0.796	0.041	−0.116	0.063	0.113	−0.067	−0.095	0.131	0.060	0.288
**Core Forest Area in a Grid**	−0.820	0.027	−0.059	0.053	0.008	−0.191	−0.120	−0.034	−0.015	0.069
**Chital Encounters per km**	0.032	0.031	−0.645	0.126	0.106	0.081	−0.037	0.167	−0.506	−0.075
**Gaur Encounters per km**	−0.100	0.058	−0.144	0.023	0.019	−0.950	−0.065	0.039	−0.033	0.023
**Sambar Encounters per km**	−0.072	0.062	−0.776	0.087	0.065	−0.268	−0.125	0.117	−0.086	−0.105
**Wild Pig Encounters per km**	0.053	−0.007	−0.153	−0.052	−0.068	−0.055	−0.050	−0.127	−0.929	−0.009
**Wild Ungulate Dung Density**	−0.084	−0.008	−0.782	−0.230	−0.201	−0.001	−0.014	−0.333	−0.019	0.118
**Livestock Dung Density**	0.075	−0.078	−0.009	−0.976	0.032	0.026	0.003	−0.011	−0.022	−0.051
**Livestock seen on transect walk**	0.040	−0.960	0.033	−0.044	−0.030	0.013	0.047	0.017	−0.009	−0.012
**Human and Livestock Trails**	0.023	−0.921	0.014	−0.028	0.012	0.042	−0.004	−0.013	0.018	−0.035
**People seen on transect walk**	0.014	−0.963	0.022	−0.026	−0.030	0.018	0.038	0.021	−0.005	−0.017
**Distance of Grid to a Protected Area**	0.165	−0.068	0.119	−0.004	0.005	0.071	0.968	−0.024	0.058	−0.055
**Distance of Grid to Night Lights**	−0.237	0.056	0.053	0.054	0.126	−0.021	−0.056	0.160	0.023	0.913
**Eigen Value**	2.143	2.723	1.712	1.045	1.026	1.047	0.991	1.016	1.149	1.006
**% Variance Explained**	13.39	17.02	10.7	6.53	6.41	6.55	6.19	6.35	7.18	6.29
**Cumulative Variance Explained**	13.39	30.41	41.11	47.64	54.06	60.6	66.8	73.15	80.33	86.62
**Ecological Attribute**	Large Undisturbed Canopied Forests	People and Cattle	Wild Ungulate Prey	Livestock	Elevation	Large Wild Ungulates	Distance to Protected Area	Rainfall	Wild Pig	Distance to Night Light
***A Priori*** ** influence of PC on Tiger Occupancy**	Negative	Positive	Negative	Positive	Negative	Negative	Negative	Positive	Negative	Positive

**Table 8:** Model selection results for estimating tiger occupancy within the Central Indian Landscape incorporating imperfect detections and covariates of landscape characteristics, prey abundance, and human disturbance represented by 10 Principal Components.

Models for detection	AIC	delta AIC	AIC weight	Model likelihood	no. par.	−2 log likelihood
psi(.),p(TigSign)	7261.85	0	1	1	3	7255.85
psi(.),p(Survey Specific)	8469.56	1207.71	0	0	36	8397.56
psi(.), p(.)	8514.35	1252.50	0	0	2	8510.35
**Models for Occupancy using best model for Detection**						
psi(PC1+PC2+PC3+PC4+PC5+PC6+PC7+PC8+PC9),p(TigSign)	6831.99	0	0.556	1	12	6807.99
psi(PC1+PC2+PC3+PC4+PC5+PC6+PC7+PC8+PC9+PC10),p(TigSign)	6832.44	0.45	0.444	0.7985	13	6806.44
psi(PC1+PC2+PC3+PC4+PC5+PC6+PC7+PC8),p(TigSign)	6852.66	20.67	0	0	11	6830.66
psi(PC1+PC2+PC3+PC4+PC5+PC7+PC8),p(Tig. Sign)	6863.40	31.41	0	0	10	6843.40
psi(PC1+PC2+PC3+PC5+PC7+PC8),p(Tig. Sign)	6985.28	153.29	0	0	9	6967.28
psi(PC1+PC2+PC3),p(Tig. Sign)	7034.80	202.81	0	0	6	70228.00

Details of the 10 Principal Components are provided in Table 6. Goodness of fit χ^2^ *P* value  = 0.11, Ĉ = 1.4, for best model.

TigSign – Tiger sign (pugmark, scat); Survey Specific – Survey specific detection.

**Table 9:** Coefficient estimates for the best model selected for estimating tiger occupancy in the Central Indian Landscape.

Occupancy Covariates	Coefficient β Estimate	SE corrected for Ĉ	Exponent (β)	Sign of variable loading on PC
Constant a1	−1.932	0.134	0.145	NA
PC1 (Forested Habitat)	−0.838	0.1	0.433	Negative
PC2 (Anthropogenic Disturbance)	0.452	0.151	1.571	Negative
PC3 (Chital, Sambar Encounters & Wild Ungulate Dung)	−0.909	0.142	0.403	Negative
PC4 (Cattle Dung)	0.258	0.137	1.294	Negative
PC5 (Elevation)	−0.436	0.107	0.647	Positive
PC7 (Distance to Protected Area)	−1.109	0.111	0.330	Positive
PC8 (Rainfall)	0.343	0.131	1.410	Positive
PC6 (Gaur Encounters)	−0.325	0.105	0.723	Negative
PC9 (Wild Pig Encounters)	−0.391	0.109	0.676	Negative
**Detection Covariates**				
Constant b1	−1.072	0.075	0.342	NA
Average Encounter Rate of Tiger Sign	0.840	0.058	2.317	NA

### Tiger Population Extents and Occupied Habitats

**Figure 4:**
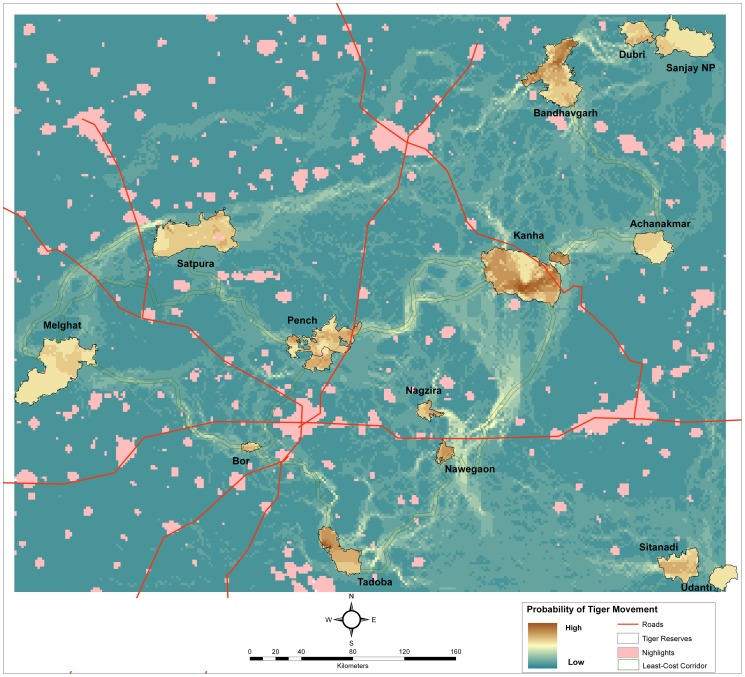
CIRCUITSCAPE model of cumulative current flow used to estimate landscape permeability to tiger movement. Tiger movement modeled as current flow within the Central Indian Landscape using tiger occupancy probability and drainage systems as conductance layers and human settlements as high resistance barriers in CIRCUITSCAPE. Light colors indicate potential habitat corridors. Note the prominent bottlenecks observed in the Kanha-Pench, Kanha-Tadoba, and Tadoba-Melghat habitat corridors.

Tiger populations within the landscape were primarily located in and around tiger reserves (Figure 1). The Pench-Kanha-Achanakmar tiger population was located in the largest patch of contiguous forest comprising 16,063 km^2^ with intermittent tiger presence recorded throughout this patch even outside the legal reserve boundaries. The Satpura-Melghat forest patch was 12,720 km^2^, while the forest patches that contained Tadoba and Bandhavgarh Tiger reserves were smaller 2,088 km^2^ and 1,902 km^2^ respectively and connected to larger adjacent patches by fragmented forests (Figures 1 and 4). Melghat Tiger Reserve had the largest critical core area that is legally mandated to be made free of human habitation (Table 1). The total protected area in the landscape under the tiger reserve system was 13,054 km^2^ with 6,395 km^2^ as core area. Tiger population extent was largest for the Tadoba population at 3,519 km^2^, while the smallest area occupied (904 km^2^) was recorded in Achanakmar (Table 1).

### Corridor models – maps and corridor cost values between source pairs

The least-cost corridor plot joining tiger reserves (Figure 1) shows that the longest corridor was between Bandhavgarh and Melghat; and the shortest corridor was between Kanha and Achanakmar (Table S8). The maximum number of barriers in the form of crossings of national highways was five for the corridor connecting Bandhavgarh to Melghat. CIRCUITSCAPE results detected bottlenecks in connectivity in corridors connecting Kanha with Pench, Tadoba with Kanha and Tadoba with Melghat (Figure 4). Alternative habitat connectivity besides the least cost corridor was detected by CIRCUITSCAPE to exist between Bandhavgarh and Melghat along the sparse ridge forests on the southern banks of the Narmada river as well as between Tadoba and Melghat with patchy connectivity observed through Bor wildlife sanctuary. Habitat connectivity between Achanakmar and Bandhavgarh was diffused with no clearly defined flow pathways being observed (Figure 4).

### Genetic structure, migrants and corridor costs

**Figure 5:**
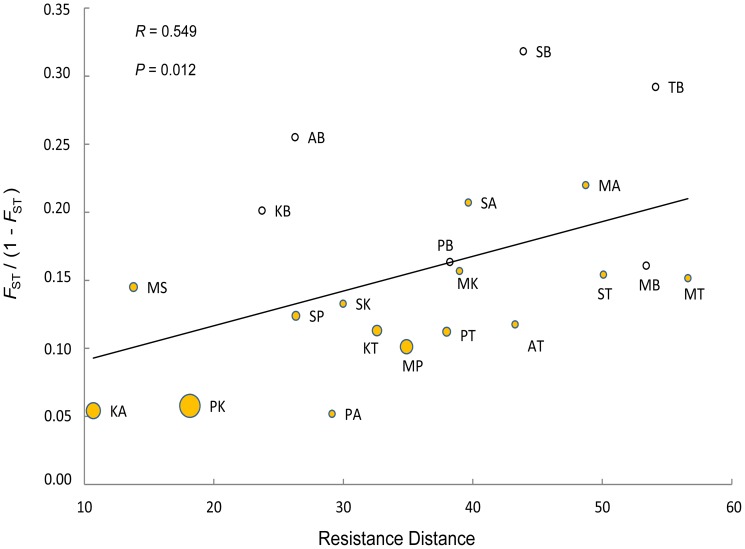
Regression of population pair-wise linearized *F*_ST_ values with corridor cost. The size of each circle is representative of the proportion of migrants shared between each population pair. Depicted corridors are Kanha-Bandhavgarh (KB), Kanha-Achanakmar (KA), Pench-Kanha (PK), Melghat-Kanha (MK), Satpura-Kanha (SK), Satpura-Pench (SP), Melghat-Pench (MP), Melghat-Satpura (MS), Kanha-Tadoba (KT), Pench-Achanakmar (PA), Melghat-Achanakmar (MA), Achanakmar-Bandhavgarh (AB), Pench-Bandhavgarh (PB), Satpura-Bandhavgarh (SB) and Melghat-Bandhavgarh (MB).

Mantel's *r* correlations between pairwise genetic distances and spatial distance metrics were positive, and showed a similar trend across all three genetic distance estimators (Table S9). The highest correlations were observed for IBR and LCC distances with genetic distance while geographical distances showed the lowest correlations with genetic distance. Linearized *R*_ST_ had weak non-significant correlations with spatial distances, compared to *F*_ST_ and *Phi*_PT_ estimates. A significant linear relationship was observed between population pairwise *F*_ST_/(1−*F*_ST_) genetic distances vs. resistance (*r* = 0.549, *p* = 0.019) and least-cost corridor distances (*r* = 0.533, *p* = 0.009). In comparison to Euclidean geographic distance correlations, this corresponded to a 29.5% and 25.7% increase in model fit for the circuit theory based IBR and the LCC models respectively. The proportion of migrants was higher between population pairs that had lower corridor costs (Figure 5). Linearized *Phi*_PT_ estimates also produced significant positive correlation with both least-cost corridor (*r* = 0.416, *p* = 0.035) and resistance distances (*r* = 0.462, *p* = 0.023). This meant a 46.2% and 31.6% increase over log-transformed Euclidean distance correlations for the circuit theory and corridor models respectively.

Correlations in partial Mantel tests showed a significant, positive relationship between genetic and resistance distances, after controlling for all competing spatial distance metrics (linearized *F*_ST_: *r*>0.48, *p*<0.02; linearized *Phi*_PT_: *r*>0.39, *p*<0.05). In contrast, after controlling the effect of resistance distance, significant positive correlation was only observed between linearized *F*_ST_ vs. geographic and least-cost corridor distances, while partial correlations between all other spatial and genetic distance metrics were either non-significant or negative. Although significant positive correlation was observed between *F*_ST_ and geographic distances in the standard Mantel tests, genetic and geographic distances were uncorrelated in the partial Mantel tests, after controlling the effect of population clusters, thus affirming that isolation-by-distance (IBD) pattern was absent in the data. Only resistance (IBR) and LCC distances retained a significant, positive partial correlation with genetic distances, when controlled for the effect of population clusters (linearized *F*_ST_: *r*>0.4, *p*<0.03; linearized *Phi*_PT_: *r*>0.4, *p*<0.04).

## Discussion

### Genetic diversity and population structure

The present study is illustrative of the general strengths and challenges in using non-invasive genetic samples to assess spatial distribution patterns in an endangered and cryptic species living in fragmented habitats. Although maternally inherited mitochondrial DNA could elucidate patterns of female gene flow, the sequencing of mitochondrial genes was not attempted as the primary objective of the study was to assess the functionality of corridors through the detection of first and second generation migrants. Hence, highly polymorphic, bi-parentally inherited, co-dominant and selectively neutral nuclear loci such as microsatellites were most suited for this purpose. Microsatellite marker amplification success was low from scats, reflecting the often poor quality of samples, typical of tropical environments [127]. Even though standardized methods of sample preservation and DNA extraction were used for the study, finding fresh scats (which may have improved results) was rare despite repeated surveys, due to the low tiger density of many sampled areas, the remoteness of the terrain, and further compounded by the heat and humidity of the region which enhanced degradation of scats. Despite these constraints a relatively sizeable sampling was achieved with the total number of identified individuals (*n* = 169) representing about 49% of the estimated population [31] in the entire landscape (Table 1). The gender proportions observed in our data are similar to the estimates obtained from camera-trap studies in the area [31]. The relatively large sample size in relation to the estimated population enhances the significance of our results.

Most individuals genotyped in this study, possessed heterozygous genotypes at>50% of typed loci, and there was no discernible differences in heterozygosity between migrant and resident individuals (Figure S2). However, unlike resident individuals, we found no migrants with fewer than five heterozygous loci, or completely heterozygous across all eleven loci. Eleven of the seventeen identified migrant individuals were slightly skewed towards a higher heterozygosity distribution, being heterozygous at eight to ten loci, while the rest of the migrants were polymorphic at five to seven loci. This could be due to the contribution by second generation migrant individuals which constituted the bulk of migrant pool. These individuals are more likely to possess a higher number of heterozygous loci than resident tigers, since they are admixed with parental genotypes from different allelic distributions.

The allelic diversity (9.1±2.2) and heterozygosity (*Ho* = 0.701±0.059, *He* = 0.754±0.039) observed in our study is typical of genetic diversity prevalent among tigers in the Indian subcontinent [23], [128], [129]. Related studies, using different microsatellite markers than the ones used here, have observed levels of average expected heterozygosity (*He*) and mean number of alleles (*A*) to be lower in other subspecies, viz. *P. t. altaica* (*He* = 0.26±0.11, *k* = 2.6±0.84 [130]), *P. t. sumatrae* (*He* = 0.493±0.039, *A* = 3.60±1.48 [129]), *P. t. jacksoni* (*He* = 0.571±0.027, *A* = 3.90±1.18 [129]) and *P. t. corbetti* (*He* = 0.670±0.027, *A* = 6.03±1.81 [129]). Interestingly, allele diversity and heterozygosity in the Indian tiger was comparable to other felid species [129], such as the jaguar (*P. onca*, *He* = 0.792±0.0137, *A* = 8.67±1.72 [131]), Asian leopard (*P. pardus*, *He* = 0.790±0.0174, *A* = 10.71±2.31 [129]), African lion (*P. leo*, *He* = 0.610±0.0348, *A* = 5.0±1.75 [129]), South American puma (*Puma concolor*, *He* = 0.774±0.0247, *A* = 7.0±1.76 [129]), and cheetah (*Acinonyx jubatus*, *He* = 0.708±0.130, *A* = 6.1±1.8 [132]). Although not directly comparable with the present study because of the different markers used, a recent study in the Satpura-Maikal landscape in Central India [64] based on seven microsatellite loci, detected similar levels of heterozygosity (*Ho* = 0.65±0.09, *He* = 0.80±0.05), and allelic diversity (*A* = 7.76±1.96), and very low genetic subdivision (mean *F*_ST_ = 0.013±0.006). Another study in the southern parts of the Central Indian landscape, focused primarily in peripheral habitats just outside the scope of this study area [66] also observed high mean heterozygosity (*Ho* = 0.54, *He* = 0.81), and allelic diversity (*A* = 11.71) levels at fourteen microsatellite loci. A range-wide study [23] conducted with five microsatellite loci showed that Indian tigers have higher heterozygosity (*Ho* = 0.70±0.16) and allelic diversity (*A* = 12.4±3.6) compared to all other tiger subspecies (*Ho* = 0.53±0.07, *A* = 7.2±1.2). The study also reported low and non-significant genetic structuring of the Central Indian tigers with the Northern (*F*_ST_ = 0.027, *p* = 0.063) and Southern Indian populations (*F*_ST_ = 0.019, *p* = 0.054). This could be attributed to a historically large effective population size and inter-population connectivity in the region of Central and Peninsular India, explaining why, despite centuries of immense trophy hunting and continued habitat fragmentation, extant tiger populations in the region currently retain close to 60% of the global genetic variation in the species [23].

By using a combination of classical population differentiation and individual clustering approaches, we were able to detect patterns of population sub-structuring in the region. Results from both the PCoA and STRUCTURE analyses suggest the presence of four genetic clusters in the area. The clustered localities occur in close proximity to each other and the overall pattern of genetic structuring observed in the landscape is concordant with existing habitat connectivity and indicative of the role that habitat fragmentation plays in shaping the distribution of allele frequencies across populations. Significant genetic structuring was also detected in the population pairwise *F*_ST_ and *R*_ST_ statistics, which may have biased parameter estimations in the STRUCTURE runs carried out without prior population information (locprior  = 0) resulting in higher hierarchical levels of population subdivision at *K* = 8. In contrast, the runs carried out with population information (locprior  = 1, *K* = 4) did not detect unnecessary genetic structuring, and ignored the prior sampling location information if the ancestry of individuals was uncorrelated [126]. The cluster solution of *K* = 4 appeared optimal for the following reasons. In cases where STRUCTURE detects multiple clustering options with similar probabilities, typically the lowest *K* value which captures much of the biological complexity in the sample is considered to be the most conservative [126]. Additionally, the presence of related individuals in our sample and the model of correlated allele frequencies used for analysis can lead to overestimation of the true *K* value [126]. Variation across the four STRUCTURE-defined population clusters was weak, but significant, in the AMOVA test, as the major portion of genetic variance was attributed to within population variation. Though the AMOVA results and *F*_ST_ statistics indicated significant pairwise structuring across all populations in the area, the pattern shared certain similarities to the population clusters identified by STRUCTURE and the ordination results in PCoA. *F*_ST_ values were generally lower for localities within the same cluster compared to pairwise estimates between different clusters. We therefore treated the genetic distance estimators including *F*_ST_ and its analogues as relative measures of population differentiation. The estimation of these parameters requires prior identification of populations and unless the population units are clearly known, such *a priori* designation may not reflect realistic biological patterns as they would only be representing *ad hoc* division of populations [133]. Importantly, the assumptions of demographic and genetic equilibrium along with long time scales under which *F*_ST_ and its analogues are based may not be suited for estimating genetic differences between populations that have undergone fragmentation or demographic fluctuation events only recently. On the other hand, the results of the model based STRUCTURE clustering approach, which partitioned individuals into relatively distinct clusters based on iterative assignments, made much more sense of the biological realities of the area as the structuring observed here is likely an artifact of recent population fragmentation.

Additionally, the differences observed between *F*_ST_ and *R*_ST_ values can provide valuable insights into the balance between genetic drift and mutation events in the studied populations [134]. In this study, pairwise genetic differences between tiger populations in the landscape showed higher *F*_ST_ values compared to *R*_ST_ values. The *F*_ST_ statistic is based on allelic identity and accounts for gene flow between different populations as the basic premise under which it estimates pairwise genetic differences [134]. In contrast, *R*_ST_ relies on allele size and single stepwise mutations are the primary contributors of genetic variation for this statistic [134]. *R*_ST_ estimations produced non-significant results across many pairwise comparisons, and had higher sampling variances compared to *F*_ST_. This suggests that populations in the area were not isolated long enough for mutations to have caused the genetic differences between populations and the primary cause of genetic structuring in the area is due to recent genetic drift. Some measure of this drift is evident from the number of private alleles found in each population, especially Bandhavgarh, which has the highest individual allele frequency compared to all other localities (Table S3).

Although the STRUCTURE and PCoA results suggest subdivision at four clusters for the Central Indian tiger population, the significant *F*_ST_ structuring observed between all populations could also be indicative of ongoing fine-scale genetic differentiation in the area. Existing patterns of population structuring are a result of past fragmentation effects and fragmentation is not a static process. Gene flow in the area is currently meager and likely to become even lower, due to continued habitat loss and burgeoning anthropogenic activity in the area. Many localities still retain marginal inter-population connectivity, as evidenced by the presence of individuals having immigrant ancestry and further substantiated by camera trapping and radio-telemetry. However, genetic isolation of almost all populations in the foreseeable future is likely if current patterns of habitat fragmentation persist. In the case of Bandhavgarh the extent of fragmentation appears to be so great that the population may have already become genetically isolated for a longer period than the other reserves. Efforts should be made to revitalize the least cost corridor connecting Bandhavgarh with Achanakmar and subsequently to the gene pool of the main Central Indian complex by a combination of restorative ecology and legal instruments (See conservation implications below).

### Migration in the Central Indian tiger population

The Central Indian landscape is a mosaic of habitats where tiger population densities [31] varying from high (Bandhavgarh 14 tigers/100 km^2^) to low (Achanakmar 0.1 tigers/100 km^2^) are juxtaposed in a matrix encompassing a range of land-use regimes from undisturbed natural forest to high density human settlements (Figure 1). Despite being one of the most fragmented TCL in India [31], some potential for gene flow across populations within the landscape exist because tigers, like most large carnivores, have the ability to disperse across great distances where habitat connectivity is present (e.g., ∼150 km linear distance between two reserves in Nepal [67]). However, in the fragmented human-dominated Terai Arc landscape of Nepal, tigers were not found to disperse across expanses of agricultural land (10 to 20 km wide) though they did traverse through stretches of degraded forest [41]. So far, the only published dispersal estimates from the Central Indian landscape are from recent genetic studies that observed first generation migration and long-range dispersal across protected areas located roughly 200 km [65] and 650 km [66] apart, suggesting that tigers may be more resilient in traversing fragmented habitats than previously reported. Extensive annual camera-trapping since 2006 has recorded tigers dispersing between Pench and Kanha, a geographic distance of more than 150 km (Jhala and Qureshi unpublished data).

Identification of migrants by the methods used in our study requires that the populations being investigated have sufficient genetic separation and low levels of migration. We found in our study that the power to detect migrant individuals varied across localities in the region. There was no power to detect migration between localities in the same cluster (such as Kanha-Achanakmar). In these situations it was difficult to tease apart actual migration events from similar allelic patterns that arose from shared population histories and low genetic separation. On the other hand, migrant identification between localities in different population clusters produced robust assignments because of distinct genetic differences. Migrant detection was highest between localities that had discernible genetic separation and had relatively intact habitat corridors with confirmed tiger presence, such as between Kanha and Pench populations where four migrants and seven likely admixed individuals were detected. Other studies by Joshi *et al.* [66] and Sharma *et al.* [65], respectively, detected one and two first generation migrants between Pench and Kanha. The intervening forest patch between Kanha and Pench not only served as a movement corridor but also had some resident tigers as evidenced from camera trap data [135]. A noninvasive genetics study by Sharma *et al.* [64] detected seventeen individuals from this corridor. At the extreme end in Bandhavgarh, no migrants were detected even though distinct genetic structuring of Bandhavgarh from other population clusters provided sufficient power to distinguish migrants. Between the Melghat and Pench populations where three individuals with migrant ancestry were obtained, there was limited but adequate power to detect migrants as genetic separation was distinct but at a lower level than Bandhavgarh. One individual sampled in Satpura was cross-assigned to Pench. The corridor between Pench and Satpura has fragmented forest connectivity and tigers were reported from this area within the last ten years [42]. Two individuals with admixed ancestry were also detected between Kanha and Tadoba, though one of these had mixed assignments with Pench as well suggesting gene flow in the last few generations. Two individuals with admixed ancestry were also detected between Kanha and Tadoba. Areas of degraded forest and low density tiger occupancy such as Nagzira and Nawegaon Wildlife Sanctuaries located strategically between Tadoba and Kanha, and Bor located between Tadoba, Pench and Melghat may serve to provide stepping stone [136] type corridor connectivity (Figure 1). These areas have resident tigers, and it seems likely that the populations are sustained by sporadic dispersing individuals from larger source populations. Joshi *et al.* [66] observed that Tadoba, Nagzira and Melghat form a genetic cluster, and based on asymmetric migration patterns in their study it appears that the tiger population in Nagzira acts as a sink population for the high density source population in Tadoba.

Based on our strict criteria of defining migrants, we designated four individuals, three males (D954, D955 and D1399) and one female (D958) that had high cross-assignments (*Q*>0.8) to a single non-home cluster, as first generation migrants. Further, a fourth male individual (D1853) that had high cross-assignments, albeit with marginally lower *Q* value (0.527) compared to the above four migrants, was also designated as a first generation migrant. The immigrant ancestry status in the remaining ten putative migrant individuals was classified as admixed based on - (i) intermediate levels of migrant probability in STRUCTURE; (ii) membership to more than one cluster in the individual *Q* assignments; and (iii) high probability of assignment to second and not to first generation immigrant ancestry state in STRUCTURE and BAYESASS.

Though one of the first generation migrants was a female, most individuals with immigrant ancestry were males (80%: 4 males out of 5 total migrants), confirming that dispersal is male-biased in tigers [41]. The low number of strict first generation migrants suggests that contemporary migration events are minor in proportion to the number of individuals with likely admixed ancestry. The identification of samples with admixed ancestry suggests that most migration events in this landscape have occurred within a few generations and is evidence that migrant tigers are able to reproduce in the new locality. This genetic evidence is further supported by field observations where a sub-adult male tiger photo-captured in Pench tiger reserve in 2006 was observed to be a territorial breeding male in Kanha tiger reserve in 2010 (Jhala and Qureshi unpublished data). The data and analyses show that tigers likely disperse between Kanha-Pench, Pench-Satpura-Melghat, Kanha-Tadoba, Melghat-Tadoba and Tadoba-Pench.

### Gene flow rates and past demography

Both the contemporary and historical analyses detected low estimates of migration rate between populations in the landscape. While this result is superficially similar to the findings of Joshi *et al.* [66], it is in sharp contrast to Sharma *et al.* [65] where they found both high historical and contemporary gene flow, although historical rates were much higher than contemporary rates. As observed in our study and by Joshi *et al.* [66], low levels of contemporary gene flow are expected given that the area is highly fragmented and extant populations occupy habitats that are completely surrounded by heavily modified landscapes, altered by agriculture and high density human settlements, thus making it difficult for tigers to disperse between populations. The highest estimate of contemporary migration was seen from Pench to Melghat, but there appears to be an asymmetric source-sink relationship between the two populations with very low migration from Melghat to Pench. Such a pattern is expected given that Pench has high tiger density (4 tigers/100 km^2^) likely serving as a source population, while the population density in Melghat is lower (2 tigers/100 km^2^). Asymmetric contemporary rates of gene flow were also apparent between Pench and Kanha, as migration from Kanha (6 tigers/100 km^2^) to Pench was much higher compared to movement in the opposite direction from Pench to Kanha. Joshi *et al.* [66] similarly detected high gene flow (>5%) between Kanha and Pench, with Kanha acting as the biggest contemporary source for immigrants. In contrast, Sharma *et al.* [65] state that only Pench is acting as a contemporary source population and contemporary gene flow from Pench to Kanha and Satpura is very high, and has remained relatively stable since historic pre-disturbance times. A comparative evaluation at historical and contemporary time scales in their study [65] showed that gene flow between the Pench–Satpura and Melghat–Satpura pairs remains similar, whereas there has been a 47–70% reduction in gene flow between Kanha–Satpura, Pench–Melghat and Kanha–Melghat. Our results showed that historical patterns of migration between the two major population clusters of Kanha and Pench were of equal, albeit low proportion in both directions, and that both Kanha and Pench acted as source populations in contemporary times as well. Although Sharma *et al.* [65] reported higher historic and contemporary gene flow rates compared to our study, both studies similarly observed that Kanha and Pench were important historic source populations and were the main drivers of gene flow in the area.

Contemporary density estimation studies using photographic capture-recapture techniques in the region [31] help explain some of the patterns observed in the gene flow analysis. During 2006, the Kanha population experienced a phase of relative expansion compared to a decline observed later in 2010. The situation was reversed in Pench where the population was relatively low during 2006–2009 compared to a later phase of expansion in 2010 [31], [42]. From the genetic sampling done in Pench during both 2007 and 2010, which pre and post-dated this period of population expansion in the locality, a range of individuals representing likely migrant or admixed ancestry to Kanha was obtained. All first generation migrants from Kanha to Pench were detected during 2007 only, when the population in Kanha was in expansion phase. In 2010, seven individuals showing admixed ancestry from Kanha were obtained in Pench. Dispersal and subsequent breeding by immigrants from Kanha to Pench during periods of population expansion in the former appears to explain the patterns of immigrant ancestry detected in the Pench population.

As parameter estimates of migration rate and theta in MIGRATE average across at least 4*Ne* past generations, [137] or several thousands of years ago in absolute time, the low long-term gene flow estimates observed in this study could suggest that populations may have been subject to historical fragmentation and genetic drift. Although ancient population fragmentation is perceived to have occurred in response to the forest clearing activity of agro-pastoralist Neolithic people during the mid-Holocene about 5,000–3,000 years ago [138], the effect is difficult to evaluate as early farmers likely did not clear forests at a scale comparable to recent centuries [139]. However, it is conceivable that the shift from hunter-gatherer to agro-pastoralist lifestyle and successful colonization of new areas would have negatively impacted large mammal populations including tigers and prey species through hunting (and reduction of prey base), as seen during human colonization events in other parts of the world [140], [141]. Evidence from an exhaustive range-wide study of tigers has detected a massive decline of about 98% in the number of tigers over the last 200 years in peninsular India [23]. The wanton slaughter of tigers and other wildlife can be glimpsed from historical hunting records of the area, where upwards of 1,000 tigers and 2,000 leopards from this region were killed within just a few decades [25]. The population decline continues apace today, as omnipresent fragmentation and rampant poaching of tigers, other carnivores and prey species have reduced tiger populations such as at Achanakmar to only a few individuals [31], and resulted in their local extirpation in adjacent reserves of Sariska [5] and Panna [29].

Alternatively, the low estimates of historic migration rate raise concerns whether these results are artifacts of sampling or population related. MIGRATE parameter estimations assume coalescent-based models of constant population sizes and mutation-migration-drift equilibrium. Departures from the equilibrium model such as recent and sudden declines in population sizes can negatively bias the posterior parameter distributions of theta and hence migration rate estimations [137]. However, this study and a recent work by Sharma *et al.* [64] did not find significant evidence of past demographic contraction. In our study, only the population of Bandhavgarh had below par *M* ratios (<0.68) suggestive of bottleneck, but the evidence was equivocal since no significant heterozygosity excess or a mode-shift in allele frequencies was detected. Though bottlenecks were not observed in this study, the analyses may be undermined in a few populations because of low sample sizes (<20 individuals), as tests such as the mode-shift in allele frequencies is known to be affected by sample size variances [106]. Also, a demographic decline may not necessarily result in a bottleneck as several factors such as duration of decline, pre-bottleneck diversity, and gene flow between populations can affect the probability of detecting a bottleneck [101], [106], [142], [143]. Furthermore, the significant heterozygosity deficit (symptomatic of recent population expansion) observed in the Pench and Kanha populations could mask signatures of population bottleneck as the addition of new individuals could increase the number of rare alleles which can bias allelic and heterozygosity distributions [106]. Demographic expansion in Pench and Kanha is likely, given the recent history of tiger population recovery in the area in the 1970s–1980s [28], [144], or about six to eight generations ago (considering a five year generation time in tigers [99]). This period is well within the window of detection for genetic bottleneck tests of heterozygosity deficiency/excess which assess short term demographic changes only, maximum up to ten generations before present [143]. We could not account for other genetic effects or demographic events which could affect the heterozygosity distributions in these two populations, although our analysis did detect some amount of higher hierarchical STRUCTURE clustering patterns which could be indicative of lineage or extended family structure, since the data definitely contained related individuals. However, even if kin-based segregation did produce a signature of heterozygosity deficit, the specific nature of the effect is difficult to evaluate without in-depth parentage and relatedness analyses, and backed by field investigations, which was beyond the scope of this study. Based on both the theta estimates and absence of bottlenecks, our results suggest that the bigger source populations in the study area have had a relatively stable population history, compared to the smaller populations, as also observed by Sharma *et al.* [65].

Our study and the recent study by Joshi *et al.* [66] had higher power to resolve hierarchical genetic structuring in the area compared to Sharma *et al.* [65] where indistinct structuring was observed. This was most likely due to the higher number of loci used in both studies compared to Sharma *et al.* [65]. Except for Tadoba where sampling was low due to logistic constraints, the small sample sizes from some other sites were due to small tiger populations. Simulation studies by Paetkau *et al.* [94] caution regarding the use of MCMC resampling methods implemented in Rannala and Mountain [95], as they tend to over-estimate migrants from a limited data set. In our case, this translates to the possibility that there may be less migration than we report in some of the smaller populations in the study system. Due to low sample sizes, individuals from Achanakmar (n = 4) and the Kanha-Pench corridor (n = 5) were not analyzed separately, as doing so resulted in overestimation of migration rates. Instead these localities were merged with the Kanha cluster, following the results of STRUCTURE assignments. Low sampling was also a problem in the study of Joshi *et al.* [66] as sites such as Kanha, Pench and Melghat were clearly under-sampled.

### Tiger Occupancy, Habitat, Prey and Human Disturbance

Occupancy has often been used as a straightforward and economical state variable in place of abundance to monitor populations [145]. Occupancy of tigers has been estimated in the Mysore-Wayanad Landscape [115] and the Corbett-Rajaji Landscape [116] and across three major tiger landscapes of India [22]. Correcting for detection bias by replicate surveys within sampling units is especially important when detection probabilities are low, sample sizes and survey lengths are small resulting in naïve occupancy estimates that are substantially negatively biased [115]. The recent availability of powerful analytical tools and software [70], [114] that permit accounting for bias caused by detections being <1 has promoted the use of correcting for such bias post data collection [115], [146] compared to more rigorous designed field methods that minimize such bias in the first place. In this study we use independent spatial replicates, each of 5 km in length, with a minimum effort of one survey for every 5 km^2^ of tiger habitat. Thus a 10×10 km^2^ grid had anywhere between 3 to 35 replicate surveys depending on the amount of tiger habitat present within that grid. The detection probability of tiger sign was high in comparison to other studies [115] due to longer spatially independent surveys as well as higher density of surveys. Therefore, the difference between naïve and bias corrected estimates of occupancy were comparatively small (3.4%). Several assumptions that are difficult to meet underlie the use of occupancy as a state variable to monitor populations [147]. Further, tiger populations are at risk of poaching and they can be severely reduced or even extirpated from prime habitats [5], [29]. Thus, model inferred occupancy from covariates of habitat, prey, and human disturbance can provide misleading inference regarding true occupancy in the case of tigers and other species that can be severely depleted by poaching, and should therefore be interpreted with caution. However, occupancy models serve to provide a good assessment of habitat suitability at a scale relevant for conservation management [70], [146]. It is in this context that we primarily use the occupancy probability in this paper so as to model habitat corridors joining tiger populations.

Detection of tiger sign was better modeled by including encounter rate of sign per km walk. Intensity of sign was found to be a reliable index of tiger abundance [22]. Detection of sign was likely to be higher with greater abundance of tigers and this was reflected in our model. Principal Components that represented distance from protected areas, prey abundance and remote canopied forests had the highest coefficients in the logit-link function that modeled tiger occupancy. All the model coefficients were in concordance with the *a priori* predictions based on our understanding of tiger ecology. Since PCs are orthogonal to each other [117] coefficients can be interpreted in terms of their sign and magnitude as the models are free from the effects of multicollinearity. The model coefficients of all site variables when used independently to model tiger occupancy as univariate models also conformed to the *a priori* predicted effects and to the coefficients obtained by the multivariate best model obtained from their PCs by PRESENCE. These results indicate that tigers are a conservation-dependent species, requiring areas having effective legal protection, good forest cover with least human impacts and high prey availability.

### Corridor models, genetic structure, migrants and dispersal costs

Although both LCP and circuit theory used tiger occupancy probability as a base layer to parameterize landscape resistance surfaces, LCP defines the optimal minimal route that is required to connect two tiger reserves [53], while the resistance layers obtained from circuit theory analysis provides an understanding of all habitat connectivity currently available between tiger reserves [118]. Circuit theory is especially important to identify bottlenecks in corridors where current is constrained to flow through a narrow channel due to high resistance of the neighboring matrix. Such bottlenecks are highlighted in Figure 4 and exist in almost all corridors connecting tiger reserves. A corridor is as good as its weakest link and if the high resistance habitat matrix surrounding these bottlenecks increase in their extent they could choke the corridor, thus forming a barrier to tiger dispersal.

In our study, the ecological distances that were generated using information from habitat heterogeneity and landscape resistance surfaces (LCCD and RD), correlated significantly with pairwise genetic distances, which validated the effectiveness of the modeled linkages in representing realistic biological scenarios. In particular, IBR models depicted well the ecological costs of movement as they accounted for multiple pathways, irregular patch effects, landscape heterogeneity and wider habitat swaths connecting populations [54]. Although the raw LCP model accounted for habitat heterogeneity while selecting a single pathway as the optimal minimal corridor, it did not correlate significantly with pairwise genetic distances (except *F*_ST_). Instead, the LCCs which were delineated by matching and buffering the pathway results from the raw LCP model with existing ground forest cover maps of the area provided a more realistic relationship of the ecological costs with observed genetic differences. In contrast, Euclidean distances (GGD and log_10_GGD) did not correlate significantly with genetic distances, due to the null model assumptions of spatial homogeneity where habitats are arrayed in an infinite lattice [124], and hence do not conform to the ground realties of fragmentation in the Central Indian landscape. The apparent IBD pattern observed due to the significant correlation of *F*_ST_ (but not *Phi*_PT_ or *R*_ST_) with geographic distance is an artifact of metapopulation structure and discrete population clusters present in the area. This observation was affirmed by non-significant partial correlations between genetic vs. geographic distances (but not LCCD and RD) using the STRUCTURE identified population clusters as a control. Apart from fragmentation induced spatial heterogeneity, genetic structure is also a result of population history.

The results of our study imply that population subdivision and genetic structure across most localities in the area was strongly associated with habitat features that offer resistance to dispersal at different intensities such as agricultural land, roads, railway lines, high density human settlements and urban infrastructure and not only by geographic distance between populations. Mantel tests showed significant positive correlation of genetic differentiation with least-cost corridors and landscape resistance surfaces associated in traversing corridors, further confirming this observation. The tiger habitats in the region are patchy with some populations still having connectivity, while being reduced or conspicuously absent in others. The best patches of contiguous forested habitat are present in the corridor between Pench and Kanha, which extends eastward to Achanakmar. Likewise, the Satpura and Melghat populations are also connected through swathes of degraded forests, which are interspersed with agricultural land and medium density human settlements. Connectivity between Pench and Satpura is fragile as parts of the linkage are disrupted by mining activities, and broken up in places by agriculture, habitations, major highways and railway lines. The population in Tadoba is linked with Kanha in a stepping stone connectivity through patchily distributed forests. Even though the intervening matrix between Tadoba, Pench and Melghat is heavily human dominated, the populations are tenuously linked by degraded forest patches and tiger occupied habitats such as Bor Wildlife Sanctuary. Bandhavgarh has linkages with forest habitat and tiger reserves further east [31], but seems isolated from tiger populations in the study area by human settlements and agricultural land. Corridors identified herein need to be given legal status, and mitigated with appropriate green infrastructure [148] for development projects within corridor habitats so as to ensure continued gene flow between populations.

### Conservation implications

It is indeed surprising that in spite of being highly fragmented [31], the tiger habitats in Central India still exist as a metapopulation with gene flow occurring between most population clusters in contemporary times. Similar conclusions were also reached in other studies [65], [66], which observed indistinct genetic structuring and low migration amongst populations, although the genetic differences were not substantial to permit unambiguous identification of migrants. These findings suggest that tigers are able to disperse through suboptimal habitat fragments, than was earlier believed [41], [149]. The results of our study underscore the importance of conserving and maintaining corridor connectivity for the continued persistence of tiger populations in the landscape. We found seventeen individuals or about 10% of the sampled tigers with migrant ancestry. As revealed from this study, the sizes of genetic population clusters are clearly beyond the boundaries of protected areas, and have to be managed in a metapopulation framework stressing the need for a landscape conservation policy in place of the current conservation policy focused on protected areas. The functionality of corridors as shown in this study has important implications for the persistence of small populations such as Achanakmar, Satpura and Melghat by the rescue effect of emigrants from the large source populations in the landscape. Apart from poaching, the ecological isolation from other source populations, due to the absence of corridors was one of the prominent causes that led to the local extinction of tigers from the nearby protected areas of Panna and Sariska [5], [29]. Now both of these areas have been repopulated through translocation of tigers from nearby reserves. A range of anthropogenic pressures are fragmenting important habitat corridors such as the ones linking Satpura with Pench, Pench with Kanha, Kanha with Tadoba, and Tadoba with Melghat. Dispersing tigers often have to negotiate passage through suboptimal prey-poor habitats and spend extended periods of time in proximity to human habitations, which greatly reduces survival and successful gene flow. The limited migration occurring in the landscape is probably due to tigers avoiding areas of human activity and high mortality due to commercial poaching or retributive killing in response to predation on livestock and humans. The eventual loss of genetic connectivity between populations in the near future, as observed currently in Bandhavgarh, is likely for most populations in the area if present trends of fragmentation continue unabated. In such situations, last resort alternative strategies such as translocation may be considered, as it could easily enrich genetic diversity by moving tigers across each of the four distinct genetic population clusters, to mimic natural patterns of gene flow. However, maintaining and restoring habitat corridors is the preferred conservation strategy to maintain genetic exchange between tiger populations, since corridors would serve the same function for other biota as well, exemplifying the role of the tiger as a flagship species.

Conservation efforts in such a fragmented and dynamic human-dominated landscape presents enormous challenges, but should be attempted in all areas where substantial habitat is still present. Our study gives renewed hope to tiger conservation efforts within the Central Indian Landscape and similar habitats with small fragmented tiger populations. We show that minimal habitat connectivity permits gene flow between populations, which is essential to maintain metapopulation connectivity. Our findings suggest that tigers could negotiate passage through stepping stone dispersal [136] as observed between Kanha-Tadoba, and Pench-Melghat. The most functional corridor was observed between Kanha and Pench, which has evidence of prey and offers the possibility of resident tigers in some of its larger forest patches [31], [135]. However, infrastructural development in the form of adding lanes to national highways and widening of railway lines, threaten to form permanent barriers even within this corridor unless proper safeguards and mitigation measures [44] are built into these development projects. Tiger range countries like India are heavily investing in infrastructure development and mining to meet the needs of a growing economy [148]. Identification of minimal habitat corridors is vital for conservation efforts of tigers and other wide-ranging fauna like elephants (*Elephas maximus*), gaurs (*Bos gaurus*), leopards (*Panthera pardus*), and dholes (*Cuon alpinus*). They need to be offered legal protection with smart green infrastructure [148] being the norm of development policies within these corridor habitats. The extinction (nearly two decades ago) of gaur in Bandhavgarh, preceding their recent reintroduction with individuals from Kanha [150], is symptomatic of habitat fragmentation events that have impaired movement with other source populations in the landscape. We demonstrate here an integrated approach to generate reliable information to document metapopulation structure of tigers and the required habitat connectivity [17] to maintain it in Central India. Legal mechanisms to safeguard these minimal corridors could potentially be the eco-sensitive category under the Environment Protection Act (1986) legislation. Currently tigers and other mega-fauna exist as a metapopulation and do exchange genetic material through functional corridors [65], [66], [128], [151]. Such opportunities are likely to be lost rapidly in the wake of new wave of development unless legal sanctity, active restoration and mitigation of development projects become the norm. Conservation policy needs to shift the focus from protected area centered preservation to landscape scale conservation where development policies incorporate a conservation ethic.

## Supporting Information

Figure S1 PCR-RFLP identification of tiger scats. (**A**) MtDNA cyt *b* alignment with GenBank and reference sequences showing polymorphism at the particular *Bam*HI restriction enzyme between tiger and leopard. (**B**) Enzyme digested bands of the 187 bp PCR product, targeting this region, showing different profiles in tiger and leopard for species identification.
(TIF)

Figure S2 Frequency of heterozygous genotypes observed at each individual multilocus genotype in all tiger individuals (n = 169) in this study.
(TIF)

Figure S3 Results of STRUCTURE analysis. (**A**). Difference in delta *K* and mean LnP(K) for an estimated number of *K* populations, in models run with (locprior  = 1) and without (locprior  = 0) prior sampling location information. (**B**). Summary barplots depicting prior and non-prior STRUCTURE runs (assumed *K* = 2 to 8), of sampled populations in central India showing cluster affiliations according to individual *Q* values. Cluster saturation at *K* = 4, indicative of four population clusters, is observed in runs carried out both with and without *a priori* location information. At *K*>4, increased sub-structuring is detected, but there is no concordance in clustering between the prior and non-prior runs.
(TIF)

Figure S4 Differences in tiger present (n = 311) and tiger absent (n = 1540), 10×10 km grids shown as violin plots in the Central Indian Landscape. All variables are normalized by z transformation to make the scales comparable.
(PDF)

Table S1 Information on the 11 microsatellite loci used in this study. Allele diversity statistics, observed (*Ho*) and expected (*He*) heterozygosity, Hardy-Weinberg equilibrium (HWE) tests, null allele frequencies and sibling probability of identity (*PI-sib*) values obtained across 169 tiger individuals. Null allele frequencies>+0.05 are italicized.
(DOCX)

Table S2 Summary of population cluster-wise genetic diversity statistics at each locus. Depicted are number of alleles (*k*), number of individuals typed (*N*), observed (*Ho*) and expected (*He*) heterozygosity, Hardy-Weinberg equilibrium (HWE) test significance and null allele frequencies (*Null*). The Achanakmar (n = 4) and Kanha-Pench corridor (n = 5) individuals are included in the Kanha cluster.
(DOCX)

Table S3 List of diagnostic alleles present in the sampled populations.
(DOCX)

Table S4 AMOVA results.
(DOCX)

Table S5 Population pair-wise *F*_ST_ (below diagonal) and *R*_ST_ (above diagonal) estimates.
(DOCX)

Table S6 Bottleneck test results for loci under different mutation models. *P* values <0.05 are depicted in italics.
(DOCX)

Table S7 Variable attributes used as site covariates in modeling tiger occupancy.
(DOCX)

Table S8 Habitat corridors, major roads, corridor cost between tiger reserves.
(DOCX)

Table S9 Results of standard and partial Mantel tests for correlation between pairwise genetic and spatial distance metrics. The correlation coefficient (*r*) and probability (*p*) are shown using three different genetic distance estimators. Significant values (*p*<0.05) are indicated by an asterisk (*).
(DOCX)

Table S10 Details of cyt *b* PCR amplification in reference samples using felid-specific (187 bp) and universal primers (309 bp). + indicates all samples which amplified. NA – not amplified.
(DOCX)

Table S11 Information on the pilot test carried out on scats (*n* = 65) for species identification of tiger samples by PCR and *Bam*HI restriction enzyme digestion.
(DOCX)

Method S1 Species identification from scat samples.
(DOCX)

Method S2 Generating the cost surface for PATHMATRIX and CIRCUITSCAPE.
(DOCX)
