# Supplementary figures and images for: Prioritizing Tiger Conservation through Landscape Genetics and Habitat Linkages
Source: PLoS One. 2014 Nov 13;9(11):e111207. doi: 10.1371/journal.pone.0111207 (PMC4230928; doi:10.1371/journal.pone.0111207)

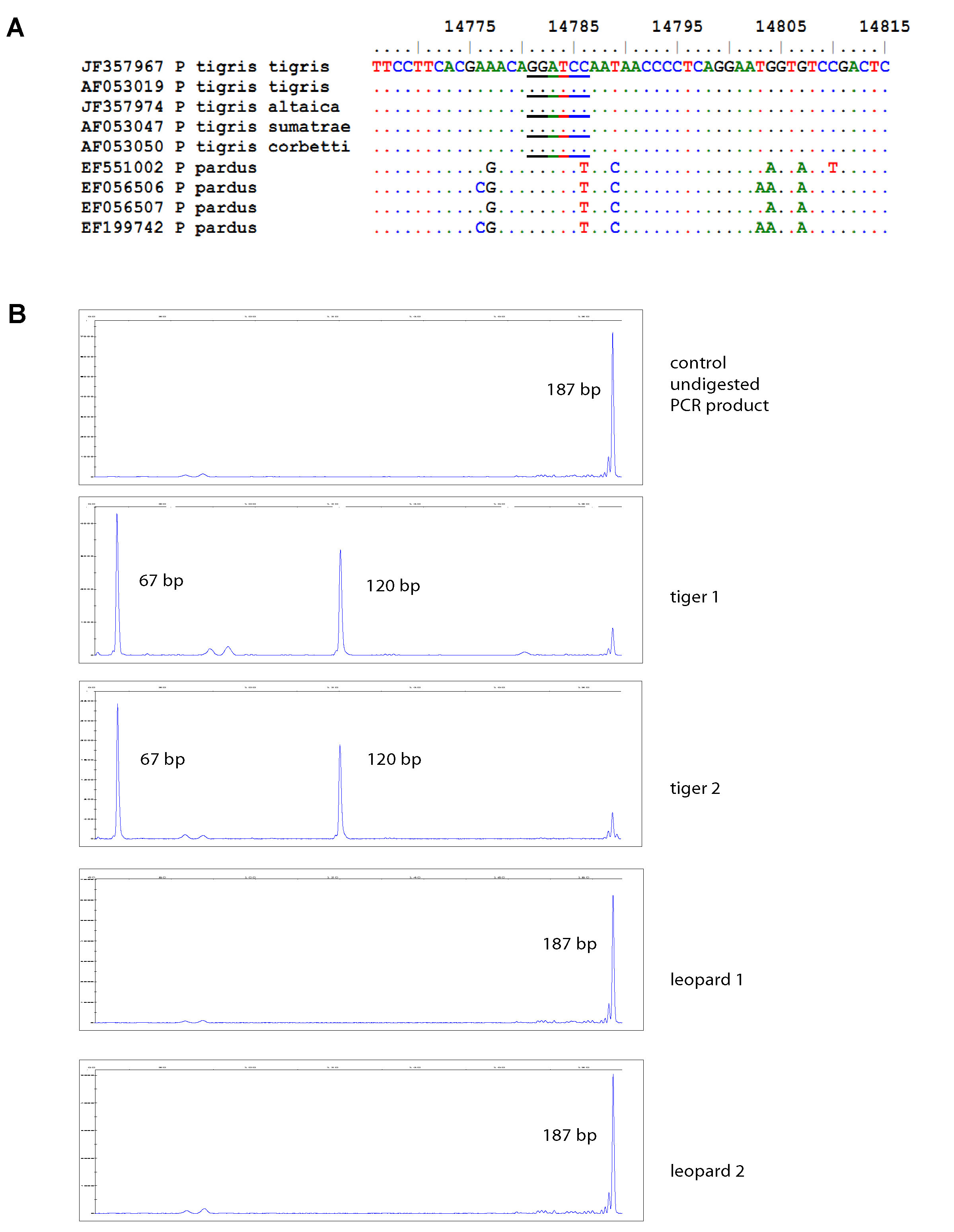

Supplement: Figure S1 — PCR-RFLP identification of tiger scats. (A) MtDNA cyt b alignment with GenBank and reference sequences showing polymorphism at the particular BamHI restriction enzyme between tiger and leopard. (B) Enzyme digested bands of the 187 bp PCR product, targeting this region, showing different profiles in tiger and leopard for species identification. (TIF) [file pone.0111207.s001.tif]

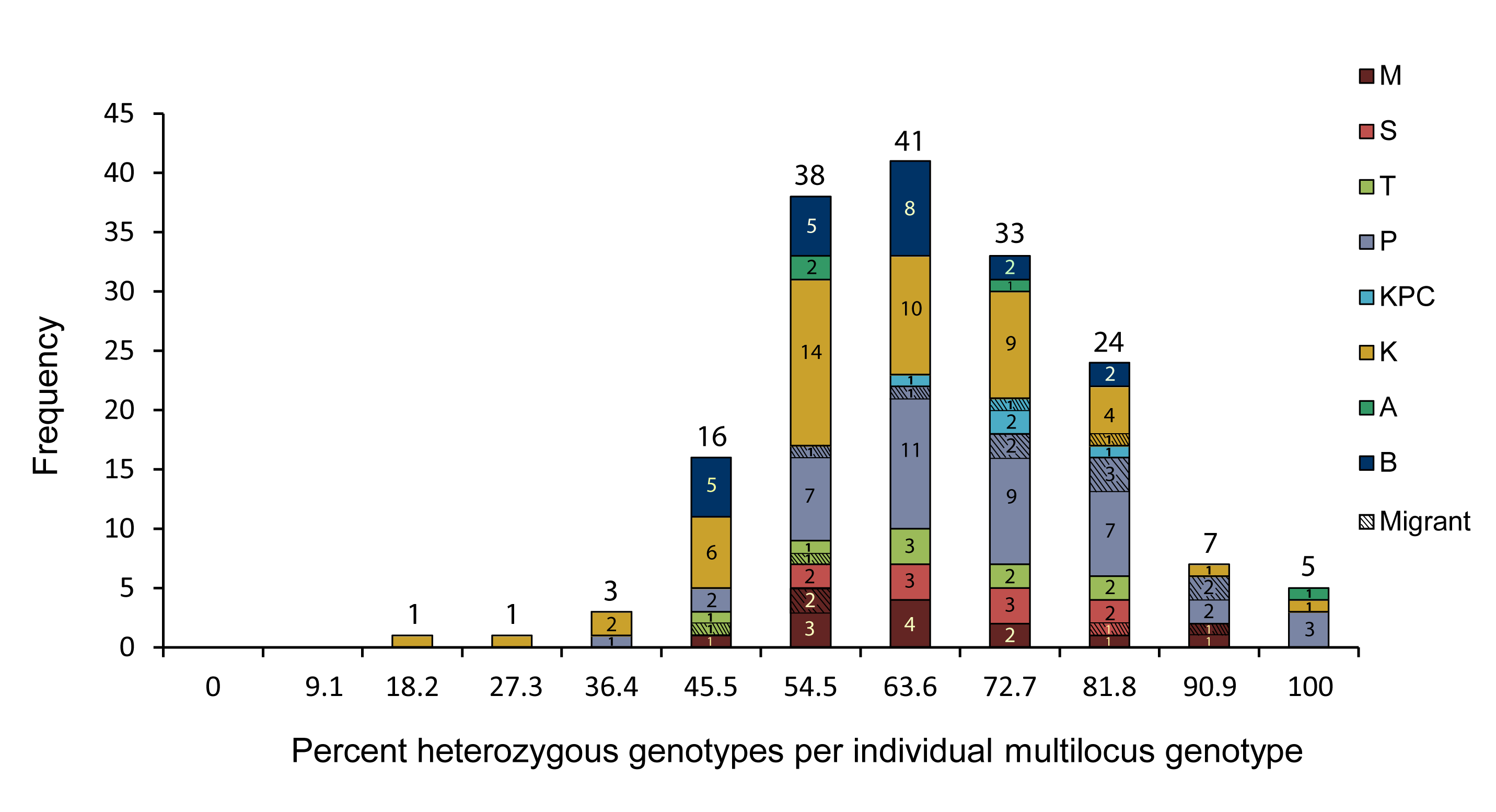

Supplement: Figure S2 — Frequency of heterozygous genotypes observed at each individual multilocus genotype in all tiger individuals (n = 169) in this study. (TIF) [file pone.0111207.s002.tif]

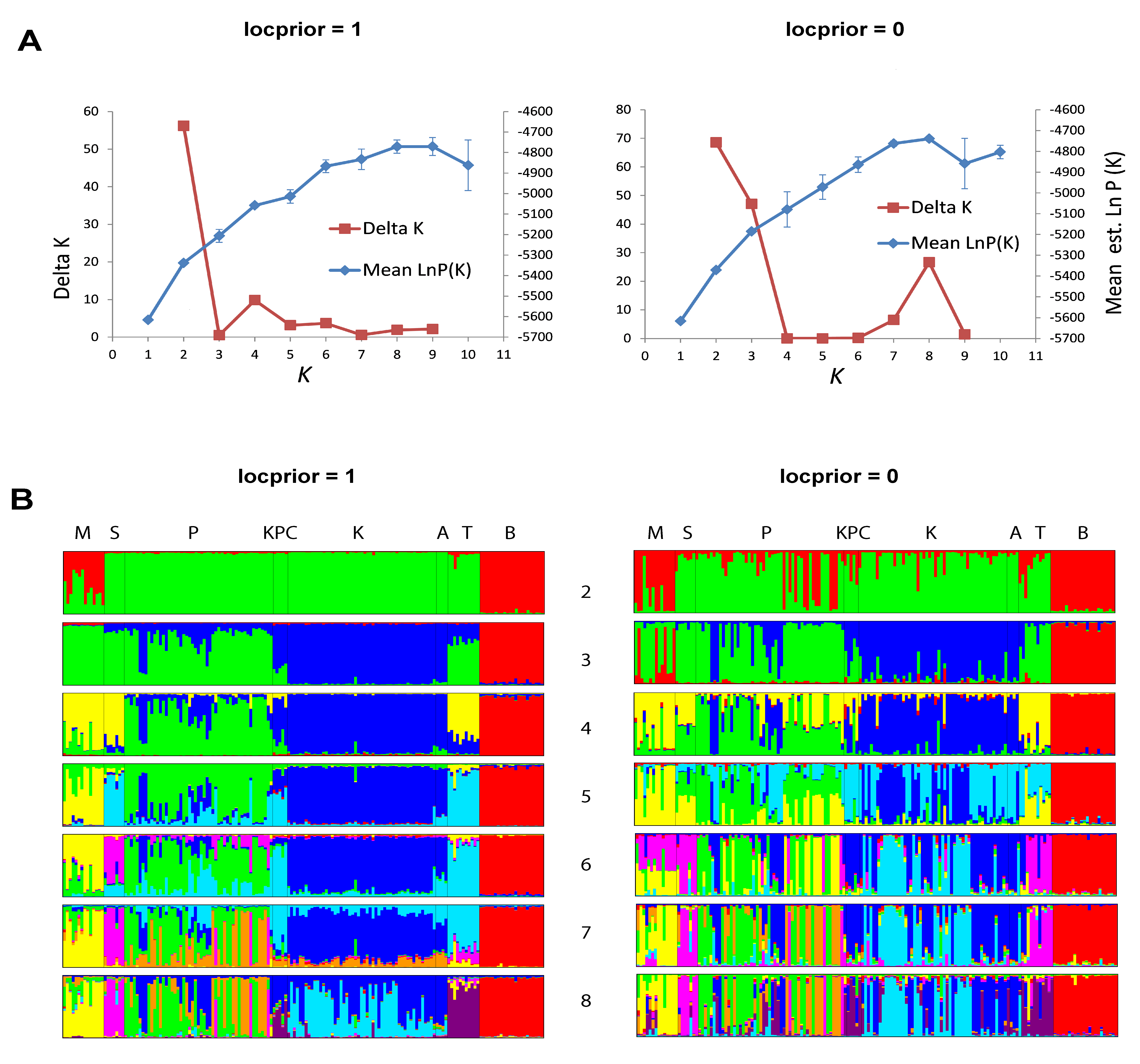

Supplement: Figure S3 — Results of STRUCTURE analysis. (A). Difference in delta K and mean LnP(K) for an estimated number of K populations, in models run with (locprior = 1) and without (locprior = 0) prior sampling location information. (B). Summary barplots depicting prior and non-prior STRUCTURE runs (assumed K = 2 to 8), of sampled populations in central India showing cluster affiliations according to individual Q values. Cluster saturation at K = 4, indicative of four population clusters, is observed in runs carried out both with and without a priori location information. At K>4, increased sub-structuring is detected, but there is no concordance in clustering between the prior and non-prior runs. (TIF) [file pone.0111207.s003.tif]
