# Supplementary material for: Prioritizing Tiger Conservation through Landscape Genetics and Habitat Linkages
Source: PLoS One. 2014 Nov 13;9(11):e111207. doi: 10.1371/journal.pone.0111207 (PMC4230928; doi:10.1371/journal.pone.0111207)

Figure S4. Differences in tiger present (n = 311) and tiger absent (n=1540), 10 x 10 km grids shown as violin plots in the Central Indian Landscape. All variables are normalized by z transformation to make the scales comparable.

a) **Figure S4 (A)**. Difference in wild ungulate encounter rates on transect walks within tiger present and tiger absent grids (t = -6.9, P < 0.001).

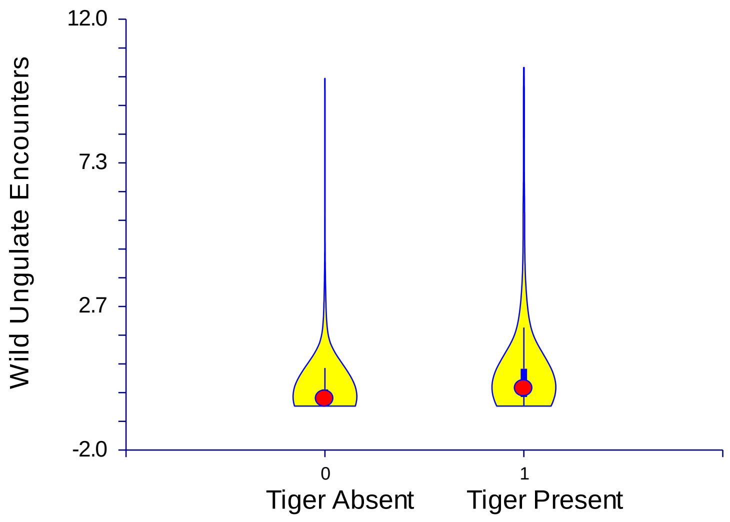

b) **Figure S4 (B)**. Difference in large wild ungulate (Sambar, Gaur, Chital and Wild Pig) encounter rates on transect walks within tiger present and tiger absent grids (t = -11.3, P < 0.001).

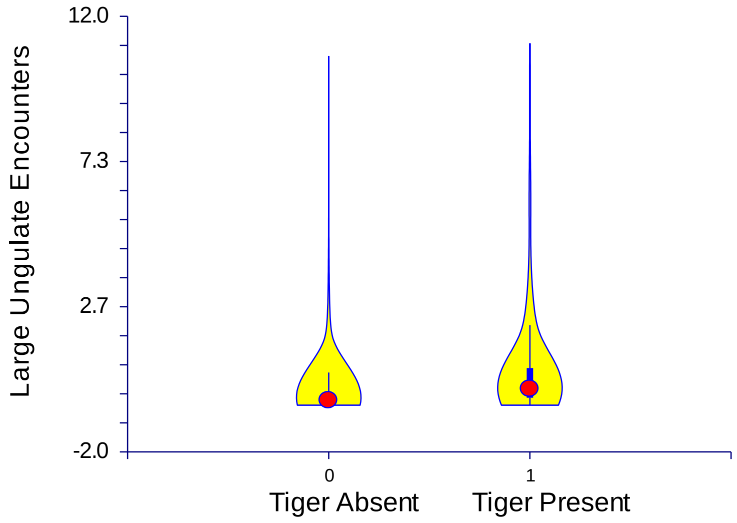

c) **Figure S4 (C)**. Difference in mean elevation of tiger present and tiger absent grids (t = 1.84, P = 0.03).

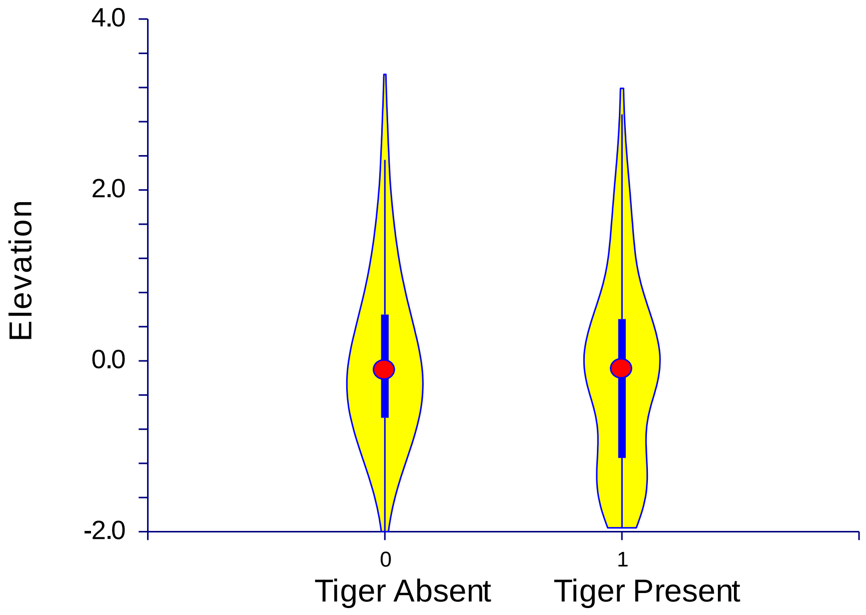

d) **Figure S4 (D)**. Difference in ruggedness (DEM CV) of tiger present and tiger absent grids (t = -5.3, P < 0.001).

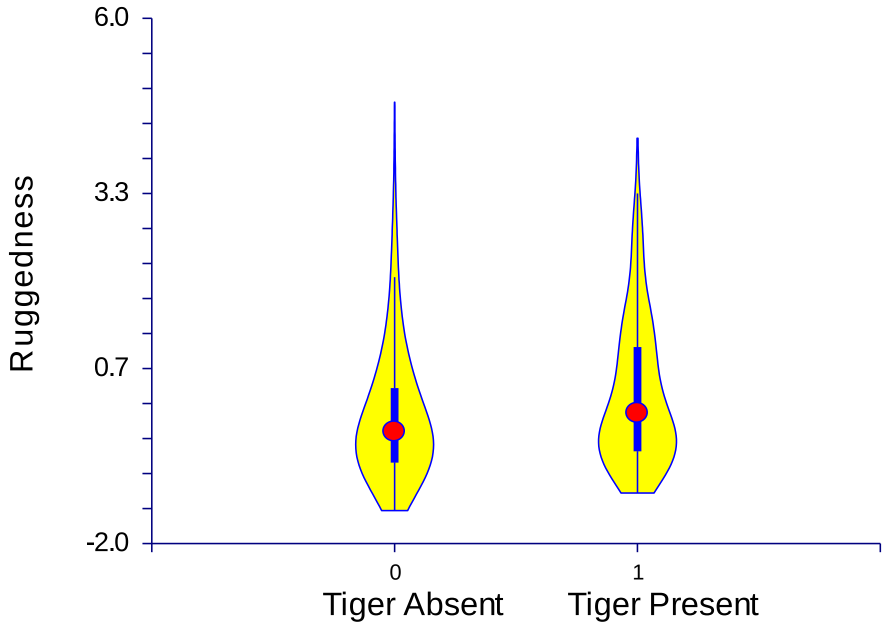

e) **Figure S4 (E)**. Difference in mean precipitation within tiger present and tiger absent grids (t = -6.62, P < 0.001).

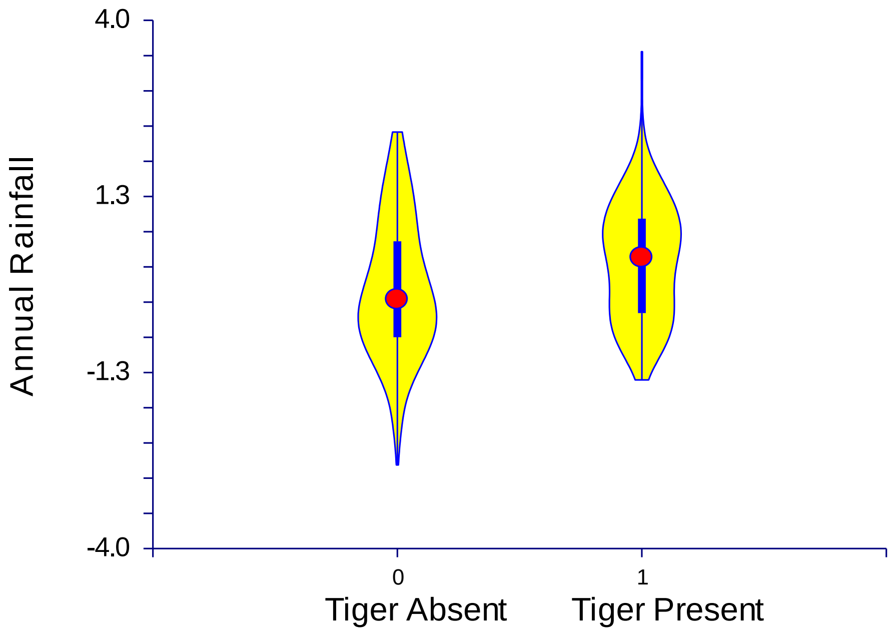

f) **Figure S4 (F)**. Difference in mean Normalized Difference Vegetation Index (NDVI) within tiger present and tiger absent grids (t = -6.647, P < 0.001).

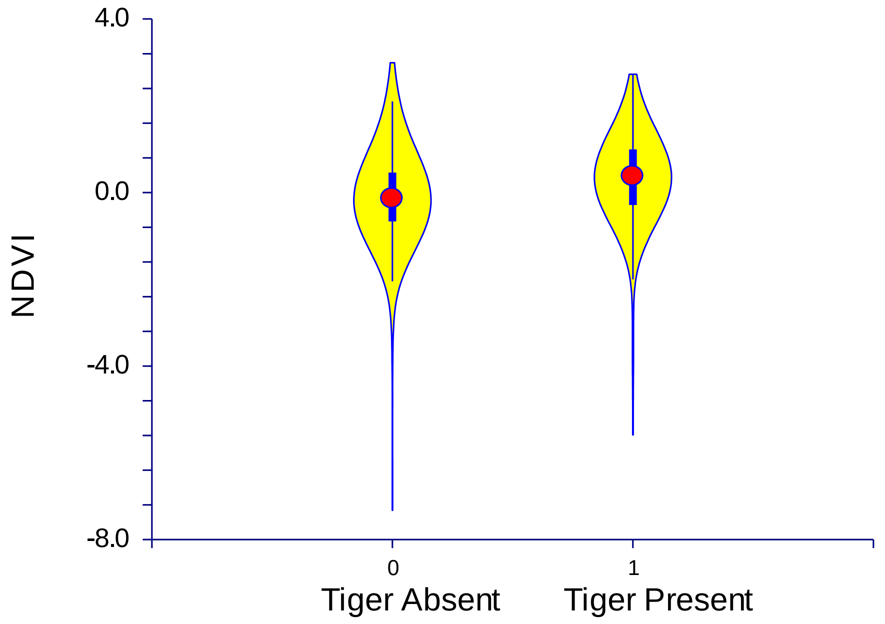

g) **Figure S4 (G)**. Difference in forest area within tiger present and tiger absent grids (t = -14.7, P < 0.001).

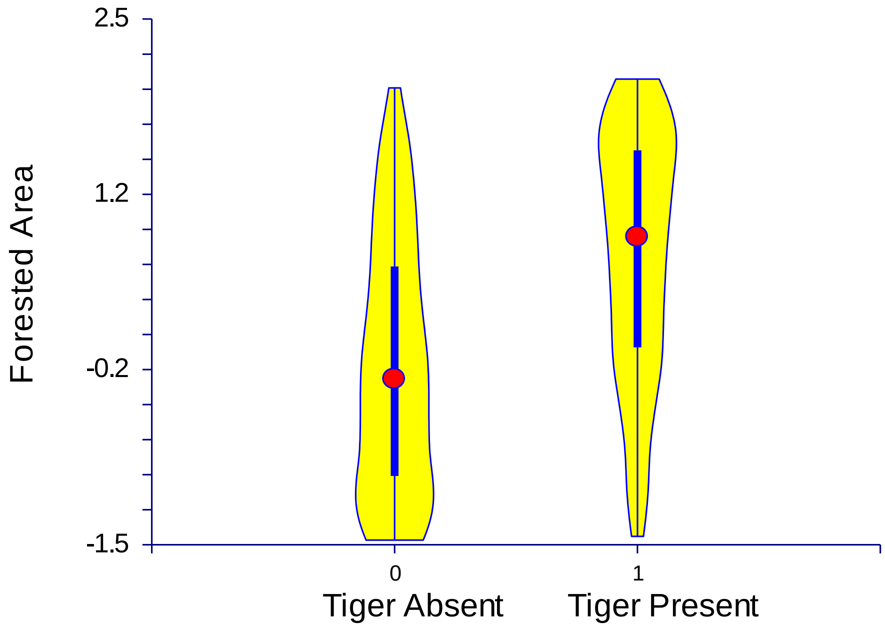

h) **Figure S4 (H).** Difference in core forest area (area of forest after removal of 2 km inward buffer from forest edge) representing relatively undisturbed forest within tiger present and tiger absent grids (t = -15.4, P < 0.001).

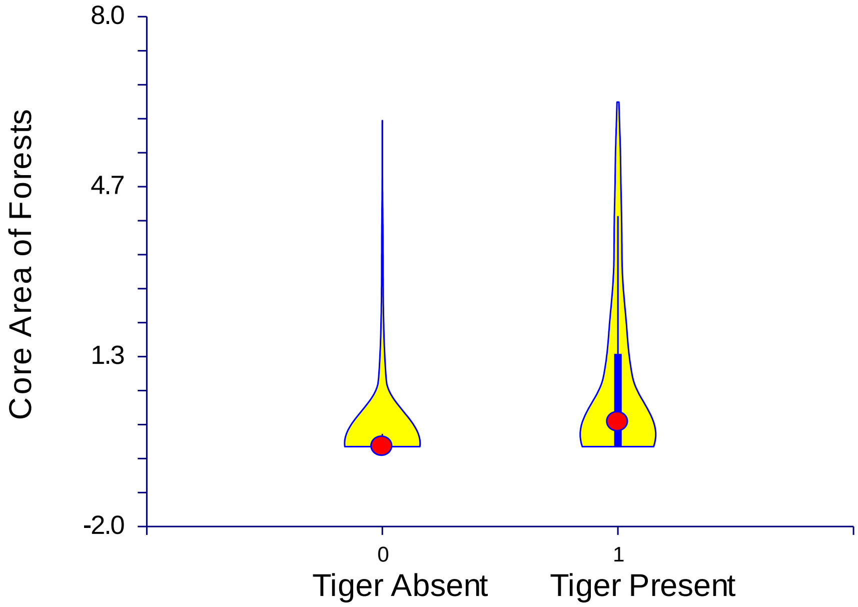

i) **Figure S4 (I).** Distance of tiger present and tiger absent grids from closest legally protected area (t = 17.3, P < 0.001).

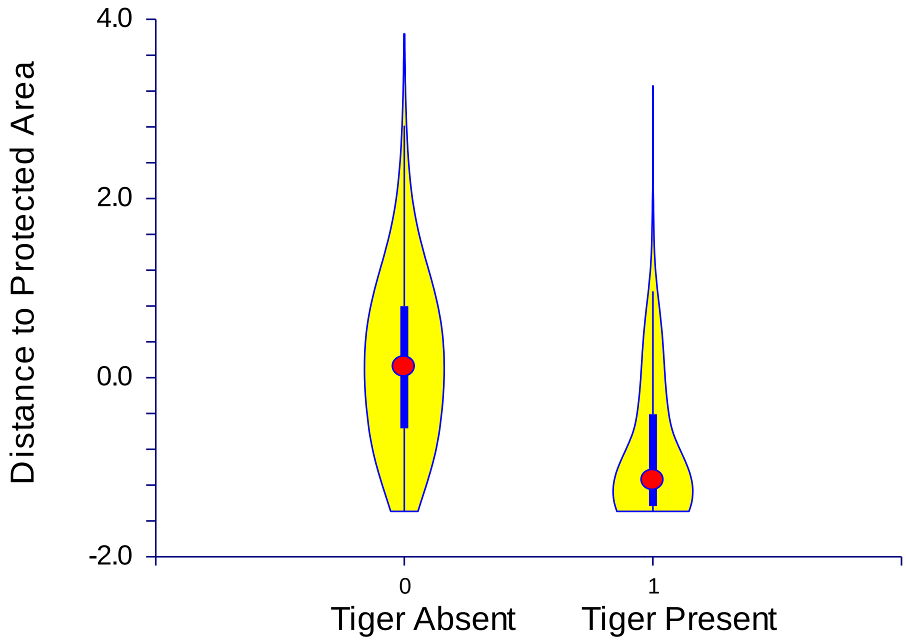

j) **Figure S4 (J).** Encounter rates of livestock on line transects within tiger present and tiger absent grids (t = 5.2, P < 0.001).

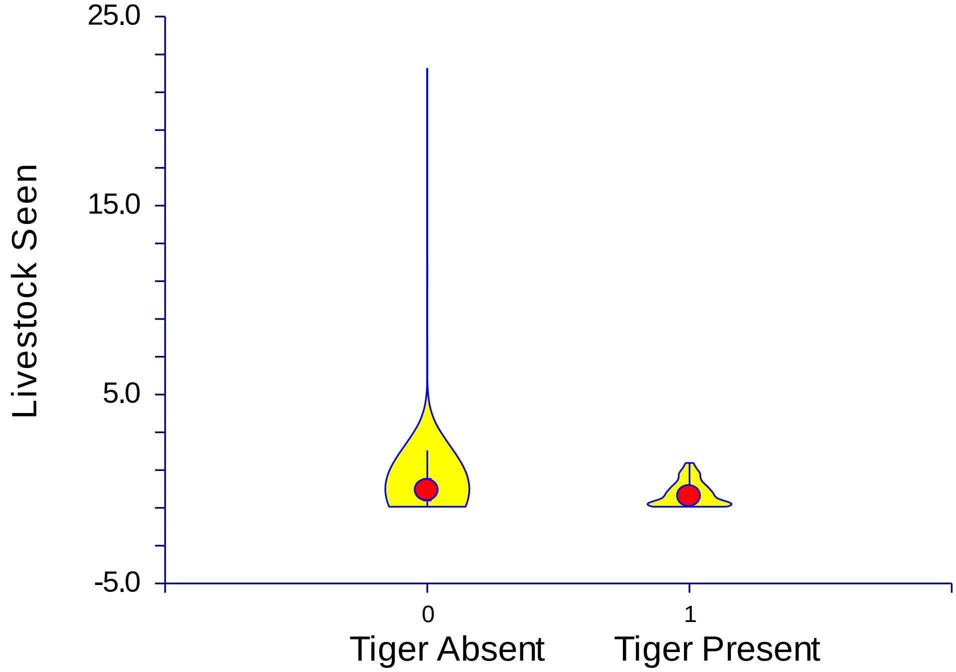

k) **Figure S4 (K).** Difference in presence of livestock and human trails on transect plots within tiger present and tiger absent grids (t = 4.06, P < 0.001).

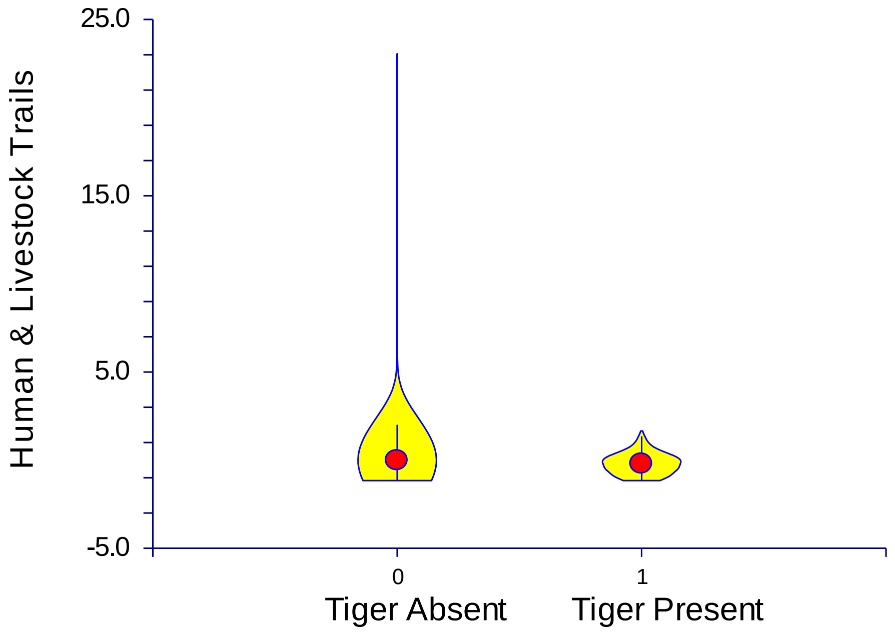

l) **Figure S4 (L).** Humans seen from line transects within tiger present and tiger absent grids (t = 4.23 P < 0.001).

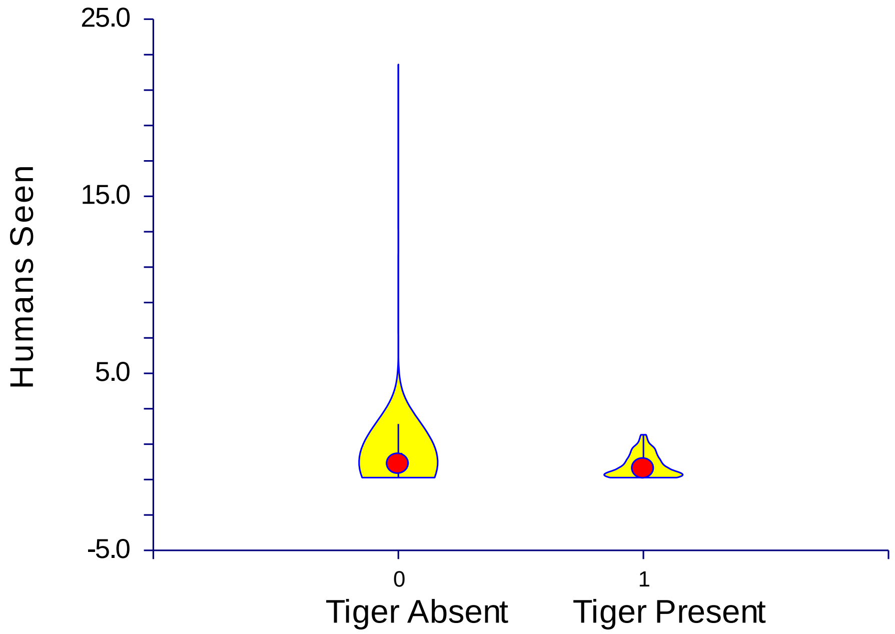

m) **Figure S4 (M).** Density of livestock dung on plots on line transects within tiger present and tiger absent grids (t = 4.02, P < 0.001).

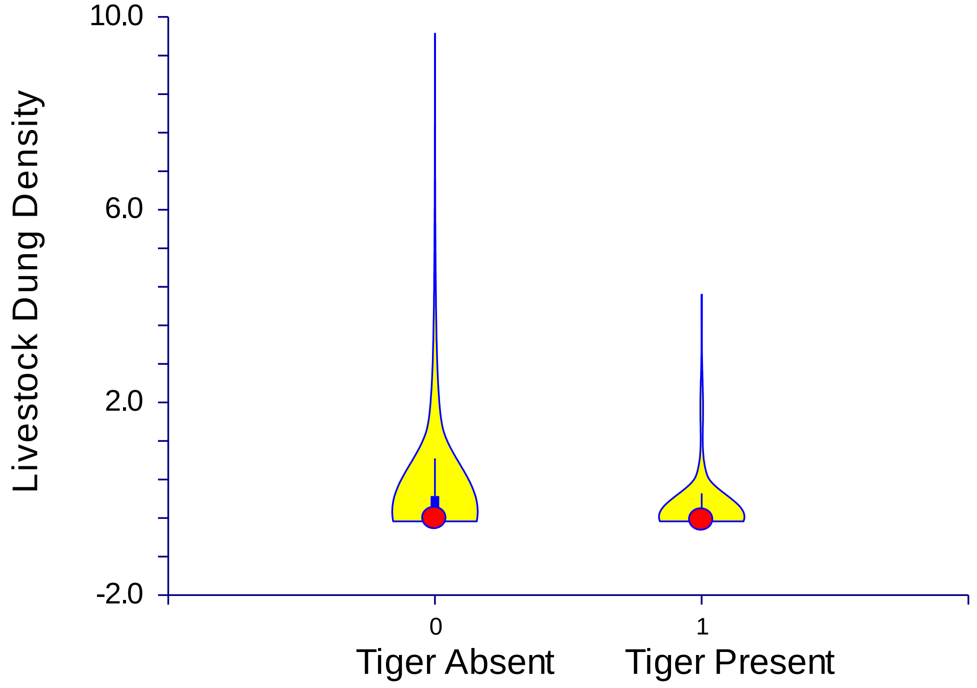

n) **Figure S4 (N).** Forest patch size to which grids with tiger present and tiger absent belonged (t = -1.19, P = 0.11).

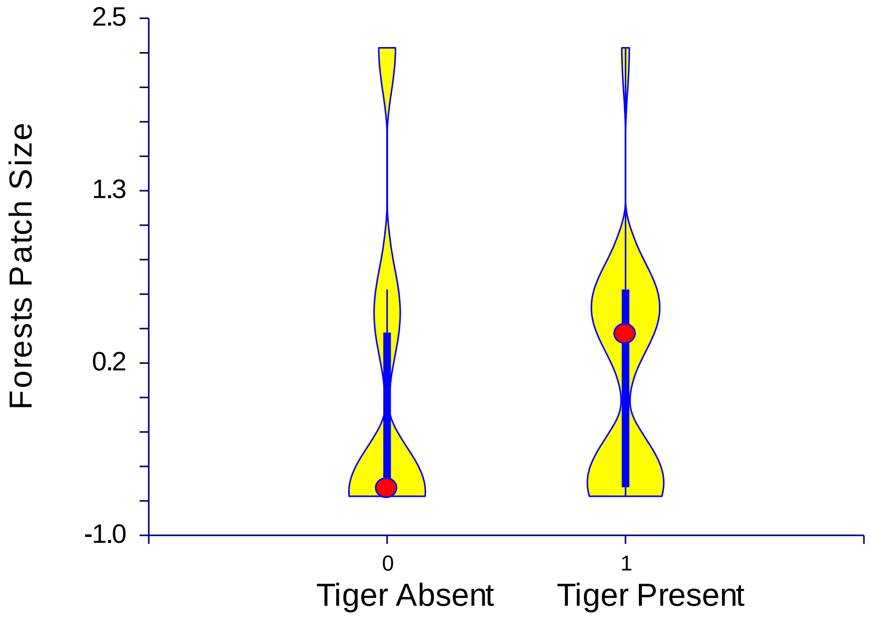

Supplement: Figure S4 — Differences in tiger present (n = 311) and tiger absent (n = 1540), 10×10 km grids shown as violin plots in the Central Indian Landscape. All variables are normalized by z transformation to make the scales comparable. (PDF) [file pone.0111207.s004.pdf]
