# Supplementary material for: Prioritizing Tiger Conservation through Landscape Genetics and Habitat Linkages
Source: PLoS One. 2014 Nov 13;9(11):e111207. doi: 10.1371/journal.pone.0111207 (PMC4230928; doi:10.1371/journal.pone.0111207)
Supplement: Table S1 — Information on the 11 microsatellite loci used in this study. Allele diversity statistics, observed (Ho) and expected (He) heterozygosity, Hardy-Weinberg equilibrium (HWE) tests, null allele frequencies and sibling probability of identity (PI-sib) values obtained across 169 tiger individuals. Null allele frequencies>+0.05 are italicized. (DOCX) [file pone.0111207.s005.docx]

**Table S1.** Information on 11 microsatellite loci used in this study. Allele diversity statistics, observed (*Ho*) and expected (*He*) heterozygosity, Hardy-Weinberg equilibrium (HWE) tests, null allele frequencies and sibling probability of identity (*PI-sib*) values obtained across 169 tiger individuals. Null allele frequencies > +0.05 are italicized.

| **Locus** | **Number of alleles** | **Allele size (basepairs)** | **Number of individuals typed (%)** | **Observed heterozygosity (*Ho*)** | **Expected heterozygosity (*He*)** | **HWE (*p* value)** | **Null allele frequency** | **Individual *PI-sib* value** | **Cumulative *PI sib* product** |
| --- | --- | --- | --- | --- | --- | --- | --- | --- | --- |
| F124 | 9 | 250-282 | 152 (89.9) | 0.711 | 0.742 | NS(0.069) | +0.014 | 4.11x10^-1^ | 3.82x10^-1^ |
| Fca304 | 10 | 115-145 | 169 (100) | 0.686 | 0.717 | NS(0.128) | +0.024 | 4.24x10^-1^ | 1.51x10^-1^ |
| F85 | 10 | 123-176 | 163 (96.4) | 0.712 | 0.782 | NS(0.107) | +0.029 | 3.79x10^-1^ | 6.2 x10^-2^ |
| Fca954 | 13 | 169-196 | 157 (92.9) | 0.701 | 0.793 | NS(0.075) | *+0.060* | 3.71x10^-1^ | 2.7 x10^-2^ |
| Fca441 | 6 | 136-157 | 167 (98.8) | 0.790 | 0.698 | *(<0.001) | -0.079 | 4.32x10^-1^ | 1.2 x10^-2^ |
| Pati15 | 13 | 190-241 | 147 (87.0) | 0.667 | 0.789 | NS(0.024)† | *+0.074* | 3.72x10^-1^ | 5.5 x10^-3^ |
| 6Hdz700 | 10 | 131-153 | 167 (98.8) | 0.725 | 0.797 | NS(0.088) | +0.049 | 3.76x10^-1^ | 2.6 x10^-3^ |
| F53 | 9 | 128-161 | 139 (82.2) | 0.612 | 0.729 | NS(0.938) | *+0.075* | 4.15x10^-1^ | 1.2 x10^-3^ |
| Pati18 | 7 | 205-229 | 136 (80.5) | 0.618 | 0.771 | *(<0.001) | *+0.104* | 3.93x10^-1^ | 5.8 x10^-4^ |
| Pati09 | 6 | 114-129 | 164 (97.0) | 0.799 | 0.710 | *(<0.001) | -0.078 | 4.32x10^-1^ | 2.9 x10^-4^ |
| Pati01 | 8 | 188-209 | 165 (97.6) | 0.685 | 0.796 | NS(0.456) | *+0.073* | 3.66x10^-1^ | 1.5 x10^-4^ |
| Average | 9.1 ± 2.2 |  | 156.9 (92.8) | 0.701 ± 0.059 | 0.754 ± 0.039 |  | +0.032 |  |  |

NS – not significant, * significant (*p*<0.05) deviation from HW expectation, † Bonferroni adjusted values
