# Supplementary material for: Prioritizing Tiger Conservation through Landscape Genetics and Habitat Linkages
Source: PLoS One. 2014 Nov 13;9(11):e111207. doi: 10.1371/journal.pone.0111207 (PMC4230928; doi:10.1371/journal.pone.0111207)
Supplement: Table S2 — Summary of population cluster-wise genetic diversity statistics at each locus. Depicted are number of alleles (k), number of individuals typed (N), observed (Ho) and expected (He) heterozygosity, Hardy-Weinberg equilibrium (HWE) test significance and null allele frequencies (Null). The Achanakmar (n = 4) and Kanha-Pench corridor (n = 5) individuals are included in the Kanha cluster. (DOCX) [file pone.0111207.s006.docx]

**Table S2.** Summary of population cluster-wise genetic diversity statistics at each locus. Depicted are number of alleles (*A*), number of individuals typed (*N*), observed (*Ho*) and expected (*He*) heterozygosity, polymorphism information content (*PIC*), null allele frequencies (*Null*) and significance tests from Hardy-Weinberg Equilibrium (HWE). MICROCHECKER detected *Null* loci are italicized.

|  | **Melghat-Satpura-Tadoba cluster (n=37)** | | | | | | |  | **Bandhavgarh (n=22)** | | | | | | |
| --- | --- | --- | --- | --- | --- | --- | --- | --- | --- | --- | --- | --- | --- | --- | --- |
| **Locus** | ***A*** | ***N*** | ***Ho*** | ***He*** | ***PIC*** | ***Null*** | **HWE** |  | ***A*** | ***N*** | ***Ho*** | ***He*** | ***PIC*** | ***Null*** | **HWE** |
| **Pati01** | 8 | 34 | 0.500 | 0.745 | 0.691 | *0.152* | *** |  | 4 | 22 | 0.773 | 0.675 | 0.592 | -0.071 | ns |
| **Pati09** | 5 | 34 | 0.941 | 0.787 | 0.738 | -0.099 | * |  | 3 | 22 | 0.864 | 0.644 | 0.555 | -0.185 | * |
| **Fca304** | 7 | 37 | 0.649 | 0.699 | 0.647 | -0.069 | ns |  | 4 | 22 | 0.227 | 0.358 | 0.328 | *0.136* | *** |
| **Fca441** | 5 | 35 | 0.771 | 0.657 | 0.603 | -0.118 | * |  | 5 | 22 | 0.864 | 0.685 | 0.607 | -0.143 | * |
| **6Hdz700** | 6 | 37 | 0.892 | 0.810 | 0.768 | -0.058 | ns |  | 5 | 22 | 0.864 | 0.674 | 0.599 | -0.141 | * |
| **F85** | 8 | 32 | 0.625 | 0.777 | 0.735 | -0.003 | ns |  | 6 | 22 | 0.682 | 0.737 | 0.679 | 0.048 | ns |
| **Fca954** | 8 | 32 | 0.688 | 0.769 | 0.724 | 0.059 | ns |  | 5 | 22 | 0.773 | 0.758 | 0.696 | -0.016 | ns |
| **F124** | 7 | 35 | 0.743 | 0.760 | 0.713 | -0.002 | ns |  | 3 | 21 | 0.476 | 0.382 | 0.319 | -0.131 | ns |
| **Pati15** | 8 | 34 | 0.588 | 0.718 | 0.660 | -0.022 | ns |  | 6 | 21 | 0.524 | 0.595 | 0.552 | 0.059 | ns |
| **F53** | 5 | 30 | 0.600 | 0.682 | 0.615 | *0.148* | *** |  | 4 | 8 | 0.625 | 0.742 | 0.636 | 0.045 | ns |
| **Pati18** | 6 | 30 | 0.567 | 0.729 | 0.670 | 0.077 | ns |  | 4 | 21 | 0.381 | 0.426 | 0.373 | 0.052 | ns |
| **Mean** | 6.6 | 33.6 | 0.688 | 0.739 | 0.688 | 0.006 |  |  | 4.5 | 20.5 | 0.641 | 0.607 | 0.540 | -0.032 |  |
| **St.Dev.** | 1.3 | 2.4 | 0.137 | 0.047 | 0.053 | 0.093 |  |  | 1.0 | 4.2 | 0.216 | 0.148 | 0.136 | 0.107 |  |
|  |  |  |  |  |  |  |  |  |  |  |  |  |  |  |  |
|  | **Pench (n=51)** | | | | | | |  | **Kanha cluster (n=59)** | | | | | | |
| **Locus** | ***A*** | ***N*** | ***Ho*** | ***He*** | ***PIC*** | ***Null*** | **HWE** |  | ***A*** | ***N*** | ***Ho*** | ***He*** | ***PIC*** | ***Null*** | **HWE** |
| **Pati01** | 7 | 51 | 0.784 | 0.820 | 0.785 | 0.017 | * |  | 8 | 59 | 0.678 | 0.644 | 0.613 | -0.042 | ns |
| **Pati09** | 6 | 51 | 0.745 | 0.630 | 0.572 | -0.122 | * |  | 6 | 59 | 0.746 | 0.640 | 0.606 | -0.084 | ns |
| **Fca304** | 10 | 51 | 0.765 | 0.735 | 0.680 | -0.025 | ns |  | 7 | 59 | 0.814 | 0.754 | 0.708 | -0.032 | ns |
| **Fca441** | 6 | 51 | 0.784 | 0.724 | 0.675 | -0.063 | ns |  | 6 | 59 | 0.780 | 0.702 | 0.643 | -0.065 | ns |
| **6Hdz700** | 8 | 49 | 0.714 | 0.713 | 0.657 | -0.002 | ns |  | 9 | 59 | 0.576 | 0.675 | 0.630 | 0.056 | ns |
| **F85** | 8 | 51 | 0.706 | 0.703 | 0.663 | -0.011 | ns |  | 8 | 58 | 0.776 | 0.767 | 0.729 | 0.033 | ns |
| **Fca954** | 10 | 48 | 0.688 | 0.767 | 0.726 | 0.043 | ns |  | 11 | 55 | 0.691 | 0.738 | 0.701 | 0.066 | ns |
| **F124** | 7 | 49 | 0.755 | 0.730 | 0.684 | -0.036 | ns |  | 5 | 47 | 0.745 | 0.774 | 0.728 | 0.022 | ns |
| **Pati15** | 9 | 46 | 0.891 | 0.804 | 0.768 | -0.066 | ns |  | 10 | 46 | 0.565 | 0.693 | 0.647 | *0.098* | *ns* |
| **F53** | 7 | 50 | 0.620 | 0.673 | 0.607 | 0.017 | ns |  | 7 | 51 | 0.608 | 0.630 | 0.588 | 0.003 | ns |
| **Pati18** | 6 | 45 | 0.800 | 0.776 | 0.732 | -0.036 | ns |  | 5 | 40 | 0.575 | 0.655 | 0.614 | *0.125* | *ns* |
| **Mean** | 7.6 | 49.3 | 0.750 | 0.734 | 0.686 | -0.026 |  |  | 7.5 | 53.8 | 0.687 | 0.697 | 0.655 | 0.016 |  |
| **St.Dev.** | 1.5 | 2.1 | 0.070 | 0.056 | 0.064 | 0.047 |  |  | 2.0 | 6.8 | 0.093 | 0.053 | 0.052 | 0.067 |  |

ns- not significant, * - *p* <0.05
