# Supplementary material for: Prioritizing Tiger Conservation through Landscape Genetics and Habitat Linkages
Source: PLoS One. 2014 Nov 13;9(11):e111207. doi: 10.1371/journal.pone.0111207 (PMC4230928; doi:10.1371/journal.pone.0111207)
Supplement: Table S3 — List of diagnostic alleles present in the sampled populations. (DOCX) [file pone.0111207.s007.docx]

**Table S3 .** List of diagnostic alleles present in the sampled populations.

| **Sl. No.** | **Population** | **Locus** | **Allele** | **Frequency** |
| --- | --- | --- | --- | --- |
| 1 | Tadoba | Pati18 | 229 | 0.050 |
| 2 | Melghat | Fca954 | 173 | 0.036 |
| 3 | Pench | Fca304 | 115 | 0.010 |
| 4 | Pench | Fca304 | 121 | 0.020 |
| 5 | Pench | Fca304 | 145 | 0.010 |
| 6 | Pench | 6Hdz700 | 133 | 0.010 |
| 7 | Pench | F85 | 123 | 0.020 |
| 8 | Pench | F124 | 282 | 0.011 |
| 9 | Pench | Pati15 | 196 | 0.043 |
| 10 | Pench | Pati15 | 202 | 0.033 |
| 11 | Kanha | 6Hdz700 | 150 | 0.009 |
| 12 | Kanha | 6Hdz700 | 153 | 0.018 |
| 13 | Kanha | Fca954 | 198 | 0.020 |
| 14 | Kanha | F53 | 132 | 0.010 |
| 15 | Achanakmar | F85 | 149 | 0.667 |
| 16 | Achanakmar | Pati15 | 232 | 0.125 |
| 17 | Bandhavgarh | Pati15 | 229 | 0.048 |
| 18 | Bandhavgarh | F53 | 157 | 0.250 |
