# Supplementary material for: Prioritizing Tiger Conservation through Landscape Genetics and Habitat Linkages
Source: PLoS One. 2014 Nov 13;9(11):e111207. doi: 10.1371/journal.pone.0111207 (PMC4230928; doi:10.1371/journal.pone.0111207)
Supplement: Table S4 — AMOVA results. (DOCX) [file pone.0111207.s008.docx]

| **TableS4.** AMOVA results | | | | | | | |
| --- | --- | --- | --- | --- | --- | --- | --- |
| **Estimator** | **Source of variance** | **d.f.** | **SS** | **MS** | **Est. Var.** | **%** |  |
| ***F*_ST_** | Among regions | 3 | 146.4 | 29.3 | 0.3 | 7 | *F*_ST_= 0.115 (*p*=0.001) |
|  | Among populations | 3 | 7.2 | 7.2 | 0.2 | 5 |  |
|  | Within populations | 331 | 1290.6 | 3.9 | 3.9 | 88 |  |
|  |  |  |  |  |  |  |  |
| ***R*_ST_** | Among regions | 3 | 995877.2 | 199175.4 | 0.0 | 0 | *R*_ST_= 0.126 (*p*=0.001) |
|  | Among populations | 3 | 82528.4 | 82528.4 | 3871.4 | 14 |  |
|  | Within populations | 331 | 8203980.2 | 24785.4 | 24785.4 | 86 |  |
|  |  |  |  |  |  |  |  |
| ***Phi*_PT_** | Among regions | 3 | 292.9 | 58.6 | 1.2 | 11 | *Phi*_PT_= 0.191 (*p*=0.001) |
|  | Among populations | 3 | 14.4 | 14.4 | 0.7 | 8 |  |
|  | Within populations | 162 | 1367.1 | 8.4 | 8.4 | 81 |  |
|  |  |  |  |  |  |  |  |
| d.f. - degrees of freedom; SS – sum of squares; MS – mean sum of squares; Est. Var. – estimated genetic variability; % - percentage variability | | | | | | | |
