# Supplementary material for: Prioritizing Tiger Conservation through Landscape Genetics and Habitat Linkages
Source: PLoS One. 2014 Nov 13;9(11):e111207. doi: 10.1371/journal.pone.0111207 (PMC4230928; doi:10.1371/journal.pone.0111207)
Supplement: Table S5 — Population pair-wise F ST (below diagonal) and R ST (above diagonal) estimates. (DOCX) [file pone.0111207.s009.docx]

**Table S5.** Population pair-wise *F*_ST_ (below diagonal) and *R*_ST_ (above diagonal) estimates.

|  | **M** | **S** | **P** | **K** | **A** | **T** | **B** |
| --- | --- | --- | --- | --- | --- | --- | --- |
| **M** | _ | 0.000 | 0.030 | 0.063** | 0.000 | 0.171** | 0.081** |
| **S** | 0.166** | _ | 0.043* | 0.049* | 0.000 | 0.141** | 0.148** |
| **P** | 0.092** | 0.110** | _ | 0.091** | 0.001 | 0.257** | 0.218** |
| **K** | 0.136** | 0.117** | 0.055** | _ | 0.084* | 0.164** | 0.183** |
| **A** | 0.180** | 0.172** | 0.049* | 0.051** | _ | 0.150** | 0.078 |
| **T** | 0.132** | 0.134** | 0.101** | 0.102** | 0.105** | _ | 0.330** |
| **B** | 0.138** | 0.241** | 0.141** | 0.168** | 0.203** | 0.226** | _ |

**p*<0.05, ***p*<0.01
