# Supplementary material for: Prioritizing Tiger Conservation through Landscape Genetics and Habitat Linkages
Source: PLoS One. 2014 Nov 13;9(11):e111207. doi: 10.1371/journal.pone.0111207 (PMC4230928; doi:10.1371/journal.pone.0111207)
Supplement: Table S6 — Bottleneck test results for loci under different mutation models. P values <0.05 are depicted in italics. (DOCX) [file pone.0111207.s010.docx]

| **Table S6**. Bottleneck test results for loci under different mutation models. *P* values < 0.05 are depicted in italics. | | | | | | | | | | | | | |
| --- | --- | --- | --- | --- | --- | --- | --- | --- | --- | --- | --- | --- | --- |
| **Population** | **Mutation model** | **Pati01** | **Pati09** | **Fca304** | **Fca441** | **6Hdz700** | **F85** | **Fca954** | **F124** | **Pati15** | **F53** | **Pati18** | **Wilcoxon's test heterozygosity deficiency/ excess** |
| M | TPM | *0.015* | 0.346 | 0.118 | 0.419 | 0.056 | 0.462 | 0.462 | 0.350 | 0.107 | 0.284 | 0.295 | 0.415/ 0.618 |
|  | SMM | *0.008* | 0.389 | 0.100 | 0.464 | 0.078 | 0.517 | 0.428 | 0.398 | 0.092 | 0.247 | 0.249 | 0.206/ 0.817 |
| S | TPM | *0.032* | 0.413 | 0.445 | 0.346 | *0.035* | 0.158 | 0.259 | 0.342 | 0.083 | 0.276 | 0.475 | 0.517/ 0.517 |
|  | SMM | *0.023* | 0.454 | 0.462 | 0.385 | *0.044* | 0.140 | 0.288 | 0.370 | 0.068 | 0.248 | 0.434 | 0.449/ 0.584 |
| T | TPM | *0.008* | 0.195 | 0.053 | 0.441 | 0.063 | 0.184 | 0.203 | 0.103 | 0.149 | 0.429 | 0.373 | 0.483/ 0.551 |
|  | SMM | 0.051 | 0.164 | 0.059 | 0.398 | *0.049* | 0.209 | 0.242 | 0.109 | 0.125 | 0.395 | 0.392 | 0.319/ 0.711 |
| MST | TPM | 0.168 | 0.274 | 0.164 | 0.263 | 0.265 | 0.289 | 0.103 | 0.475 | 0.249 | 0.197 | 0.408 | 0.139/ 0.879 |
|  | SMM | 0.107 | 0.352 | 0.109 | 0.328 | 0.360 | 0.203 | 0.056 | 0.386 | 0.172 | 0.136 | 0.321 | *0.034/* 0.973 |
| P | TPM | 0.914 | 0.129 | 0.262 | 0.497 | 0.085 | 0.081 | 0.060 | 0.277 | 0.342 | 0.103 | 0.186 | *0.011/* 0.992 |
|  | SMM | 0.136 | 0.091 | *0.010* | 0.398 | *0.044* | 0.041 | *0.028* | 0.196 | 0.234 | 0.062 | 0.249 | *0.008/* 0.994 |
| K | TPM | *0.045* | 0.156 | 0.318 | 0.368 | *0.015* | 0.253 | *0.008* | *0.018* | *0.010* | 0.065 | 0.335 | *0.003/* 0.998 |
|  | SMM | *0.023* | 0.101 | 0.216 | 0.283 | *0.007* | 0.161 | *0.003* | *0.026* | *0.003* | *0.032* | 0.256 | *0.003/* 0.998 |
| KA | TPM | 0.174 | 0.192 | 0.363 | 0.339 | 0.054 | 0.098 | *0.011* | 0.397 | 0.087 | 0.159 | 0.213 | *0.001/* 0.999 |
|  | SMM | 0.110 | 0.115 | 0.249 | 0.247 | *0.024* | *0.044* | *0.004* | 0.496 | *0.043* | 0.108 | 0.157 | *0.001/* 0.999 |
| B | TPM | 0.285 | 0.078 | *0.042* | 0.412 | 0.372 | 0.390 | 0.164 | 0.214 | *0.036* | 0.267 | 0.075 | 0.289/ 0.577 |
|  | SMM | 0.315 | 0.082 | *0.030* | 0.362 | 0.304 | 0.319 | 0.206 | 0.203 | 0.231 | 0.296 | 0.058 | 0.207/ 0.413 |
| All | TPM | 0.275 | 0.509 | *0.049* | 0.313 | 0.243 | 0.204 | *0.037* | *0.033* | *0.034* | 0.104 | 0.377 | *0.008/* 0.994 |
|  | SMM | 0.402 | 0.393 | *0.020* | 0.188 | 0.124 | 0.104 | *0.016* | *0.013* | *0.011* | *0.049* | 0.507 | *0.002/* 0.998 |
