# Supplementary material for: Prioritizing Tiger Conservation through Landscape Genetics and Habitat Linkages
Source: PLoS One. 2014 Nov 13;9(11):e111207. doi: 10.1371/journal.pone.0111207 (PMC4230928; doi:10.1371/journal.pone.0111207)
Supplement: Table S7 — Variable attributes used as site covariates in modeling tiger occupancy. (DOCX) [file pone.0111207.s011.docx]

**Table S7.** Variable attributes used as site covariates in modeling tiger occupancy

|  | **Variable** | **Coefficient** | **Standard Error** | ***A Priori* Effect on Tiger Occupancy** | **Model Coefficient Statistical Significance at**  ***P* < 0.05** | **Inference** |
| --- | --- | --- | --- | --- | --- | --- |
| 1 | Elevation | -0.155954 | 0.072098 | Tigers prefer lower elevations | Significant negative effect | As Expected *a priori* |
| 2 | Ruggedness of Terrain | 0.254187 | 0.07148 | Tigers prefer areas with varying topography | Significant positive effect | As Expected *a priori* |
| 3 | Rainfall | 0.489701 | 0.078954 | Tigers prefer areas with higher precipitation in Central India | Significant positive effect | As Expected *a priori* |
| 4 | NDVI | 0.495762 | 0.093011 | Tigers prefer higher forest cover areas in Central India | Significant positive effect | As Expected *a priori* |
| 5 | Wild Ungulate Encounters | 1.073336 | 0.162443 | Tigers prefer areas with higher wild ungulate density | Significant positive effect | As Expected *a priori* |
| 6 | Encounters of Chital, Sambar, Gaur & Wild Pig | 1.279904 | 0.192511 | Tigers prefer areas with higher large wild ungulates | Significant positive effect | As Expected *a priori* |
| 7 | Forest Area in Grid | 0.811786 | 0.079945 | Grids with core tiger habitat are preferred | Significant positive effect | As Expected *a priori* |
| 8 | Human-Livestock Trails on Transect Plots | -0.57678 | 0.11427 | Grids with less human-livestock usage are preferred | Significant negative effect | As Expected *a priori* |
| 9 | Signs of Wood Cutting | 0.035698 | 0.051649 | Grids with less human extraction is preferred | No effect | Wood cutting signs persist for long, as well as the staff are reluctant to report illegal logging in their beat (not reliable data) |
| 10 | Signs of Lopping | -6.281753 | 1.272362 | Grids with less human extraction is preferred | Significant negative effect | As Expected *a priori* |
| 11 | People seen from Transect plots | -0.549956 | 0.111098 | Grids with less human usage are preferred | Significant negative effect | As Expected *a priori* |
| 12 | Livestock seen from transect plots | -0.653749 | 0.110678 | Grids with less livestock are preferred | Significant negative effect | As Expected *a priori* |
| 13 | Wild Ungulate dung density | 0.770176 | 0.102966 | Grids with high wild ungulates are preferred | Significant positive effect | As Expected *a priori* |
| 14 | Cattle Dung Density | -0.473553 | 0.118857 | Tigers prefer grids that have less livestock | Significant negative effect | As Expected *a priori* |
| 15 | Distance of Grid to Protected Area | -1.285609 | 0.106254 | Tigers are more likely to occur within and close to legally protected areas | Significant negative effect | As Expected *a priori* |
| 16 | Distance to Night Lights | 0.027377 | 0.072404 | Tigers avoid areas near human habitation | No effect | Coefficient not significant  (tourism resorts and infrastructural development near tiger reserves confounds effect) |
| 17 | Distance to Roads | -0.126593 | 0.075538 | Tigers prefer areas away from roads | No effect | Roads criss-cross almost all remaining tiger habitats |
| 18 | Core area of Forest in a Grid | 0.769403 | 0.085948 | Tigers prefer undisturbed large core areas compared to edge forests | Significant positive effect | As Expected *a priori* |
| 19 | Size of the contiguous forest patch | -0.03217 | 0.07576 | Tigers are more likely to occur within grids that are part of large forest patches | No effect | There were only 5 forest patches to which all grids belonged and these were not too variable in size for testing their effect in the model. |
