# Supplementary material for: Prioritizing Tiger Conservation through Landscape Genetics and Habitat Linkages
Source: PLoS One. 2014 Nov 13;9(11):e111207. doi: 10.1371/journal.pone.0111207 (PMC4230928; doi:10.1371/journal.pone.0111207)
Supplement: Table S8 — Habitat corridors, major roads, corridor cost between tiger reserves. (DOCX) [file pone.0111207.s012.docx]

| **Table S8.** Habitat corridors, major roads, corridor cost between tiger reserves. | | | | | | |
| --- | --- | --- | --- | --- | --- | --- |
| **Code** | **Corridor** | **National Highways** | **Least-cost Corridor distance (km)** | **Least-cost pathway distance (km)** | **Least-cost pathway cost (cost units)** | **Resistance distance** |
| MB | Melghat-Bandhavgarh | 5 | 533.7 | 465.2 | 282,737.4 | 43.3 |
| TB | Tadoba-Bandhavgarh | 3 | 531.7 | 428.6 | 274,221.7 | 50.1 |
| MA | Melghat-Achanakmar | 3 | 450.7 | 488.8 | 305,166.7 | 29.1 |
| SB | Satpura-Bandhavgarh | 3 | 408.8 | 307.2 | 201,087.7 | 54.1 |
| ST | Satpura-Tadoba | 2 | 407.7 | 288.3 | 196,605.0 | 43.9 |
| AT | Achanakmar-Tadoba | 2 | 396.6 | 395.6 | 228,089.5 | 26.3 |
| MK | Melghat-Kanha | 3 | 375.1 | 349.4 | 237,607.2 | 48.7 |
| SA | Satpura-Achanakmar | 1 | 325.8 | 409.2 | 237,172.8 | 38.2 |
| KT | Kanha-Tadoba | 1 | 321.0 | 254.2 | 159,535.7 | 13.8 |
| PB | Pench-Bandhavgarh | 3 | 282.4 | 281.6 | 188,230.8 | 32.6 |
| MP | MelghatPench | 2 | 251.3 | 149.2 | 147,208.3 | 10.7 |
| SK | Satpura-Kanha | 1 | 250.1 | 269.9 | 169,613.2 | 53.4 |
| MT | Melghat-Tadoba | 3 | 242.0 | 241.8 | 179,165.7 | 39.6 |
| KB | Kanha-Bandhavgarh | 2 | 210.7 | 131.6 | 105,018.1 | 56.6 |
| PA | Pench-Achanakmar | 2 | 199.4 | 257.4 | 142,122.9 | 18.1 |
| MS | Melghat-Satpura | 2 | 165.7 | 89 | 78,899.3 | 26.3 |
| AB | Achanakmar-Bandhavgarh | 1 | 135.1 | 122.1 | 95,849.9 | 38.0 |
| PT | Pench-Tadoba | 2 | 133.0 | 132.3 | 100,973.9 | 30.0 |
| SP | Satpura-Pench | 0 | 126.3 | 86.4 | 82,396.3 | 23.7 |
| PK | Pench-Kanha | 1 | 123.8 | 118 | 74,563.5 | 39.0 |
| KA | Kanha-Achanakmar | 1 | 75.7 | 71.2 | 51,024.9 | 34.9 |
