# Supplementary material for: Prioritizing Tiger Conservation through Landscape Genetics and Habitat Linkages
Source: PLoS One. 2014 Nov 13;9(11):e111207. doi: 10.1371/journal.pone.0111207 (PMC4230928; doi:10.1371/journal.pone.0111207)
Supplement: Table S9 — Results of standard and partial Mantel tests for correlation between pairwise genetic and spatial distance metrics. The correlation coefficient (r) and probability (p) are shown using three different genetic distance estimators. Significant values (p<0.05) are indicated by an asterisk (*). (DOCX) [file pone.0111207.s013.docx]

| **Table S9.** Results of standard and partial Mantel tests for correlation between pairwise genetic and spatial distance metrics. The correlation coefficient (*r*) and probability (*p*) are shown using three different genetic distance estimators. Significant values (*p*<0.05) are indicated by an asterisk (*). | | | | | | | | | |
| --- | --- | --- | --- | --- | --- | --- | --- | --- | --- |
| **Correlation** | **Covariate** | ***F*_ST_/(1-*F*_ST_)** | |  | ***Phi*_PT_/(1-*Phi*_PT_)** | |  | ***R*_ST_/(1-*R*_ST_)** | |
|  |  | ***r*** | ***p*** |  | ***r*** | ***p*** |  | ***r*** | ***p*** |
| **Standard Mantel test** |  |  |  |  |  |  |  |  |  |
| Genetic x GGD |  | 0.433 | 0.039* |  | 0.335 | 0.098 |  | 0.125 | 0.314 |
| Genetic x Log_10_ GGD |  | 0.424 | 0.036* |  | 0.316 | 0.121 |  | 0.164 | 0.244 |
| Genetic x LCPD |  | 0.427 | 0.022* |  | 0.335 | 0.076 |  | 0.103 | 0.367 |
| Genetic x LCCD |  | 0.533 | 0.009* |  | 0.416 | 0.035* |  | 0.248 | 0.125 |
| Genetic x RD |  | 0.549 | 0.012* |  | 0.462 | 0.023* |  | 0.293 | 0.171 |
| **Partial Mantel test** |  |  |  |  |  |  |  |  |  |
| Genetic x GGD | cluster | 0.374 | 0.051 |  | 0.272 | 0.128 |  | 0.078 | 0.369 |
| Genetic x Log10 GGD | cluster | 0.361 | 0.053 |  | 0.252 | 0.145 |  | 0.117 | 0.331 |
| Genetic x LCPD | cluster | 0.366 | 0.059 |  | 0.273 | 0.130 |  | 0.054 | 0.403 |
| Genetic x LCCD | cluster | 0.446 | 0.022* |  | 0.337 | 0.081 |  | 0.143 | 0.254 |
| Genetic x RD | cluster | 0.540 | 0.012* |  | 0.445 | 0.036* |  | 0.275 | 0.070 |
| Genetic x GGD | Log_10_ GGD | 0.101 | 0.323 |  | 0.116 | 0.308 |  | -0.135 | 0.308 |
| Genetic x GGD | LCPD | 0.083 | 0.377 |  | 0.020 | 0.473 |  | 0.167 | 0.288 |
| Genetic x GGD | LCCD | -0.075 | 0.388 |  | -0.078 | 0.385 |  | -0.124 | 0.261 |
| Genetic x GGD | RD | 0.405 | 0.042* |  | 0.286 | 0.117 |  | 0.076 | 0.368 |
| Genetic x Log_10_ GGD | GGD | 0.019 | 0.487 |  | -0.029 | 0.446 |  | 0.170 | 0.257 |
| Genetic x Log_10_ GGD | LCPD | 0.045 | 0.432 |  | -0.033 | 0.444 |  | 0.258 | 0.118 |
| Genetic x Log_10_ GGD | LCCD | -0.158 | 0.259 |  | -0.153 | 0.264 |  | -0.148 | 0.283 |
| Genetic x Log_10_ GGD | RD | 0.395 | 0.043* |  | 0.267 | 0.133 |  | 0.117 | 0.325 |
| Genetic x LCPD | GGD | -0.017 | 0.487 |  | 0.029 | 0.438 |  | -0.151 | 0.310 |
| Genetic x LCPD | Log_10_ GGD | 0.073 | 0.377 |  | 0.119 | 0.304 |  | -0.227 | 0.147 |
| Genetic x LCPD | LCCD | -0.168 | 0.256 |  | -0.119 | 0.334 |  | -0.310 | 0.063 |
| Genetic x LCPD | RD | 0.373 | 0.059 |  | 0.267 | 0.135 |  | 0.039 | 0.430 |
| Genetic x LCCD | GGD | 0.379 | 0.059 |  | 0.294 | 0.117 |  | 0.344 | 0.021* |
| Genetic x LCCD | Log_10_ GGD | 0.384 | 0.048 |  | 0.319 | 0.083 |  | 0.238 | 0.148 |
| Genetic x LCCD | LCPD | 0.384 | 0.054 |  | 0.286 | 0.119 |  | 0.377 | 0.017* |
| Genetic x LCCD | RD | 0.469 | 0.025* |  | 0.334 | 0.083 |  | 0.179 | 0.202 |
| Genetic x RD | GGD | 0.529 | 0.009* |  | 0.432 | 0.029* |  | 0.277 | 0.073 |
| Genetic x RD | Log_10_ GGD | 0.529 | 0.009* |  | 0.433 | 0.029* |  | 0.272 | 0.076 |
| Genetic x RD | LCPD | 0.514 | 0.013* |  | 0.421 | 0.037* |  | 0.279 | 0.071 |
| Genetic x RD | LCCD | 0.489 | 0.016* |  | 0.394 | 0.046* |  | 0.239 | 0.111 |
