# Supplementary material for: Prioritizing Tiger Conservation through Landscape Genetics and Habitat Linkages
Source: PLoS One. 2014 Nov 13;9(11):e111207. doi: 10.1371/journal.pone.0111207 (PMC4230928; doi:10.1371/journal.pone.0111207)
Supplement: Table S10 — Details of cyt b PCR amplification in reference samples using felid-specific (187 bp) and universal primers (309 bp). + indicates all samples which amplified. NA – not amplified. (DOCX) [file pone.0111207.s014.docx]

**Table S10** – Details of cyt *b* PCR amplification in reference samples using felid-specific (187 bp) and universal primers (309 bp). + indicates all samples which amplified. NA – not amplified

| **Sl No** | **Species** | **Sample ID** | **Description, Locality** | **Sample type** | **cyt *b* 187** | **cyt *b* 309** |
| --- | --- | --- | --- | --- | --- | --- |
| 1 | *Panthera tigris* | D254 | Kanha, Madhya Pradesh | Blood | + | + |
| 2 | *P. tigris* | D528 | Kanha, Madhya Pradesh | Blood | + | + |
| 3 | *P. tigris* | D398 | Kanha, Madhya Pradesh | Blood | + | + |
| 4 | *P. tigris* | D404 | Kanha, Madhya Pradesh | Blood | + | + |
| 5 | *P. tigris* | D426 | Kanha, Madhya Pradesh | Blood | + | + |
| 6 | *P. tigris* | D442 | Kanha, Madhya Pradesh | Blood | + | + |
| 7 | *P. tigris* | D430 | Kanha, Madhya Pradesh | Blood | + | + |
| 8 | *P. tigris* | D401 | Kanha, Madhya Pradesh | Blood | + | + |
| 9 | *P. tigris* | D407 | Kanha, Madhya Pradesh | Blood | + | + |
| 10 | *P. tigris* | D399 | Kanha, Madhya Pradesh | Blood | + | + |
| 11 | *P. tigris* | D402 | Kanha, Madhya Pradesh | Blood | + | + |
| 12 | *P. tigris* | D253 | Kanha, Madhya Pradesh | Blood | + | + |
| 13 | *P. tigris* | D532 | Pench., Madhya Pradesh | Blood | + | + |
| 14 | *P. tigris* | D431 | Pench., Madhya Pradesh | Blood | + | + |
| 15 | *P. tigris* | D1122 | Pench., Madhya Pradesh | Blood | + | + |
| 16 | *P. tigris* | D530 | Ranthambhore, Rajasthan | Blood | + | + |
| 17 | *P. tigris* | D531 | Ranthambhore, Rajasthan | Blood | + | + |
| 18 | *P. tigris* | D432 | Ranthambhore, Rajasthan | Blood | + | + |
| 19 | *P. pardus* | D680 | Ranthambhore, Rajasthan | Tissue | + | + |
| 20 | *P. pardus* | D801 | Kanha, Madhya Pradesh | Scat | + | + |
| 21 | *P. pardus* | D223 | Gir National Park, Gujarat | Tissue | + | + |
| 22 | *P. pardus* | Ppa1 | Uttarakhand | Tissue | + | + |
| 23 | *P. pardus* | Ppa2 | Uttarakhand | Tissue | + | + |
| 24 | *P. pardus* | Ppa3 | Uttarakhand | Tissue | + | + |
| 25 | *P. pardus* | Ppa4 | Uttarakhand | Tissue | + | + |
| 26 | *P. pardus* | Ppa5 | Uttarakhand | Tissue | + | + |
| 27 | *P. pardus* | Ppa6 | Uttarakhand | Tissue | + | + |
| 28 | *Hyaena hyaena* | D537 | Kutch, Gujarat | Blood | - | + |
| 29 | *H. hyaena* | D557 | Kutch, Gujarat | Blood | - | + |
| 30 | *H. hyaena* | D926 | Rajaji N. P., Uttarakhand | Tissue | - | + |
| 31 | *H. hyaena* | D943 | Ranthambhore, Rajasthan | Tissue | - | + |
| 32 | *H. hyaena* | D944 | Ranthambhore, Rajasthan | Tissue | - | + |
| 33 | *H. hyaena* | D945 | Sawai Mansingh WLS, Rajasthan | Tissue | - | + |
| 34 | *Canis lupus pallipes* | D1 | Velavadar N.P., Gujarat | Blood | - | + |
| 35 | *C. l. pallipes* | D50 | Velavadar N.P., Gujarat | Blood | - | + |
| 36 | *C. l. pallipes* | D163 | Velavadar N.P, Gujarat | Blood | - | + |
| 37 | *C. l. familiaris* | D177 | Local breed, Assam | Hair | - | + |
| 38 | *C. l. familiaris* | D190 | Ghaddi Dog, Palampur, Himachal | Hair | - | + |
| 39 | *C. l. familiaris* | D231 | Gir N.P., Gujarat | Blood | - | + |
| 40 | *C. aureus* | D156 | Velavadar N.P., Gujarat | Tissue | - | + |
| 41 | *C. aureus* | D172 | Velavadar N.P., Gujarat | Blood | - | + |
| 42 | *C. aureus* | D246 | Haryana | Tissue | - | + |
| 43 | *Melursus ursinus* | D1351 | Kanha-Pench Corridor, M.P. | Scat | - | + |
| 44 | *Capra hircus* |  | Dehradun, Uttarakhand | Tissue | - | + |
| 45 | *Sus scrofa* | D174 | Velavadar N.P., Gujarat | Tissue | - | + |
| 46 | *Homo sapiens* |  | B Yumnam (experimenter) | Blood | - | + |
