# Supplementary material for: Prioritizing Tiger Conservation through Landscape Genetics and Habitat Linkages
Source: PLoS One. 2014 Nov 13;9(11):e111207. doi: 10.1371/journal.pone.0111207 (PMC4230928; doi:10.1371/journal.pone.0111207)
Supplement: Table S11 — Information on the pilot test carried out on scats (n = 65) for species identification of tiger samples by PCR and BamHI restriction enzyme digestion. (DOCX) [file pone.0111207.s015.docx]

**Table S11**. Information on the pilot test carried out on scats (*n*=65) for species identification of tiger samples by PCR and *Bam*HI restriction enzyme digestion.

| **Sl No** | **Sample ID** | **Locality** | **Band 1 (bp)** | **Band 2 (bp)** | **Species ID, Remarks** |
| --- | --- | --- | --- | --- | --- |
| 1 | D495 | Kanha Tiger Reserve | 67 | 120 | Tiger |
| 2 | D496 | Kanha Tiger Reserve | 67 | 120 | Tiger |
| 3 | D499 | Kanha Tiger Reserve | 67 | 120 | Tiger |
| 4 | D500 | Kanha Tiger Reserve | 67 | 120 | Tiger |
| 5 | D501 | Kanha Tiger Reserve | 67 | 120 | Tiger |
| 6 | D502 | Kanha Tiger Reserve | 67 | 120 | Tiger |
| 7 | D503 | Kanha Tiger Reserve | 67 | 120 | Tiger |
| 8 | D504 | Kanha Tiger Reserve | 67 | 120 | Tiger |
| 9 | D505 | Kanha Tiger Reserve | 67 | 120 | Tiger |
| 10 | D506 | Kanha Tiger Reserve | 67 | 120 | Tiger |
| 11 | D507 | Kanha Tiger Reserve | -- | -- | not established, PCR failed |
| 12 | D510 | Kanha Tiger Reserve | 67 | 120 | Tiger |
| 13 | D512 | Kanha Tiger Reserve | 67 | 120 | Tiger |
| 14 | D516 | Kanha Tiger Reserve | 67 | 120 | Tiger |
| 15 | D517 | Kanha Tiger Reserve | 67 | 120 | Tiger |
| 16 | D518 | Kanha Tiger Reserve | 67 | 120 | Tiger |
| 17 | D569 | Pench Tiger Reserve | 67 | 120 | Tiger |
| 18 | D570 | Pench Tiger Reserve | 67 | 120 | Tiger |
| 19 | D571 | Pench Tiger Reserve | 67 | 120 | Tiger |
| 20 | D572 | Pench Tiger Reserve | 67 | 120 | Tiger |
| 21 | D574 | Pench Tiger Reserve | 67 | 120 | Tiger |
| 22 | D576 | Pench Tiger Reserve | 67 | 120 | Tiger |
| 23 | D577 | Pench Tiger Reserve | 67 | 120 | Tiger |
| 24 | D578 | Pench Tiger Reserve | 67 | 120 | Tiger |
| 25 | D579 | Pench Tiger Reserve | 67 | 120 | Tiger |
| 26 | D580 | Pench Tiger Reserve | 67 | 120 | Tiger |
| 27 | D581 | Pench Tiger Reserve | 67 | 120 | Tiger |
| 28 | D582 | Pench Tiger Reserve | 67 | 120 | Tiger |
| 29 | D792 | KanhaTiger Reserve | 67 | 120 | Tiger |
| 30 | D793 | KanhaTiger Reserve | 67 | 120 | Tiger |
| 31 | D794 | KanhaTiger Reserve | 67 | 120 | Tiger |
| 32 | D795 | KanhaTiger Reserve | 67 | 120 | Tiger |
| 33 | D796 | KanhaTiger Reserve | 67 | 120 | Tiger |
| 34 | D797 | KanhaTiger Reserve | 67 | 120 | Tiger |
| 35 | D798 | KanhaTiger Reserve | 67 | 120 | Tiger |
| 36 | D800 | KanhaTiger Reserve | 67 | 120 | Tiger |
| 37 | D1371 | Pench Tiger Reserve | 67 | 120 | Tiger |
| 38 | D1373 | Pench Tiger Reserve | 67 | 120 | Tiger |
| 39 | D1374 | Pench Tiger Reserve | 67 | 120 | Tiger |
| 40 | D1376 | Pench Tiger Reserve | -- | -- | not established, PCR failed |
| 41 | D1377 | Pench Tiger Reserve | 67 | 120 | Tiger |
| 42 | D1378 | Pench Tiger Reserve | 67 | 120 | Tiger |
| 43 | D1379 | Pench Tiger Reserve | 67 | 120 | Tiger |
| 44 | D1380 | Pench Tiger Reserve | 67 | 120 | Tiger |
| 45 | D1381 | Pench Tiger Reserve | 67 | 120 | Tiger |
| 46 | D1382 | Pench Tiger Reserve | 67 | 120 | Tiger |
| 47 | D1383 | Pench Tiger Reserve | 67 | 120 | Tiger |
| 48 | D1384 | Pench Tiger Reserve | 67 | 120 | Tiger |
| 49 | D1385 | Pench Tiger Reserve | 67 | 120 | Tiger |
| 50 | D1390 | Pench Tiger Reserve | 67 | 120 | Tiger |
| 51 | D1391 | Pench Tiger Reserve | 187 | -- | leopard |
| 52 | D1392 | Pench Tiger Reserve | 187 | -- | leopard |
| 53 | D1393 | Pench Tiger Reserve | 67 | 120 | Tiger |
| 54 | D1394 | Pench Tiger Reserve | -- | -- | not established, PCR failed |
| 55 | D1395 | Pench Tiger Reserve | -- | -- | not established, PCR failed |
| 56 | D1396 | Pench Tiger Reserve | 187 | -- | leopard |
| 57 | D1397 | Pench Tiger Reserve | 187 | -- | leopard |
| 58 | D1398 | Pench Tiger Reserve | 187 | -- | leopard |
| 59 | D1404 | Pench Tiger Reserve | 187 | -- | leopard |
| 60 | D1184 | KanhaTiger Reserve | 67 | 120 | Tiger |
| 61 | D1185 | KanhaTiger Reserve | 67 | 120 | Tiger |
| 62 | D1288 | Kanha- Pench corridor | 187 | -- | leopard |
| 63 | D1291 | Kanha- Pench corridor | 187 | -- | leopard |
| 64 | D1292 | Kanha- Pench corridor | 187 | -- | leopard |
| 65 | D1296 | Kanha- Pench corridor | 187 | -- | leopard |
