# Supplementary material for: Prioritizing Tiger Conservation through Landscape Genetics and Habitat Linkages
Source: PLoS One. 2014 Nov 13;9(11):e111207. doi: 10.1371/journal.pone.0111207 (PMC4230928; doi:10.1371/journal.pone.0111207)
Supplement: Method S1 — Species identification from scat samples. (DOCX) [file pone.0111207.s016.docx]

**Supplementary Method S1. Species identification from scat samples**

Due to similarity in scat morphology and the sympatric distribution of tigers and leopards, field identification can at times be erroneous (Sugimoto *et al.* 2006). Furthermore, successful genetic identification can be compounded by experimental failures in amplifying molecular markers from degraded scat DNA extracts (Mukherjee *et al.* 2007). In lieu of these difficulties and the usefulness of inter-species sequence polymorphism at restriction enzyme sites in assigning species identity (Nagata *et al.* 2005, Mukherjee *et al.* 2010), we used Primer3 (Rozen and Skaletsky, 2000) to design two novel primers, PtiL-14,716 ([6-FAM]5'-ACTTCATCCTTCCGTTTATCGTCT-3') and PtiH-14,901 ([6-FAM]5'-GGACGAGTAGTATGAGGGTTAGGAT-3') at positions 14,716-14,901 bp of the mitochondrial DNA cytochrome *b* (mtDNA cyt*b*) gene in tiger and related felid species. This region flanks a particular diagnostic *Bam*HI restriction enzyme site, GGATCC, at 14,782 bp position of the tiger cyt *b* gene, but not in leopard (Figure S1A). Cleavage of the 187 bp PCR product into two bands of 120 and 67 bp was diagnostic in tiger (which possesses the restriction site) but not leopard, which lack the cleavage site (Figure S1B). The 5’end of both primers were tagged with the fluorescent dye 6-Fam^TM^, to enable band resolution of the *Ba*mHI digested PCR products by capillary electrophoresis. PCR and subsequent sizing of fluorescent bands was done using Genemapper v3.7 (Applied Biosystems, USA

The PtiL-14,716 and PtiH-14,901 primers were initially tested on DNA extracts from reference blood, tissue, hair and scat samples (Table S10). A conserved universal primer pair targeting a 309 bp band of the cyt *b* gene across diverse taxa (Kocher *et al.* 1989) was also used as a control with all samples. Once the conditions were standardized, a pilot amplification and restriction enzyme digestion was carried out using 65 carnivore scats (Table S11), in order to check performance of the new protocol on degraded DNA extracts.

PCR was carried out using 2 µl of tissue DNA or 5 µl of scat DNA extract in a total volume of 15 µl, using 7.5 µl of 2X HotStarTaq Polymerase enzyme mix (QIAGEN Ag, Germany). The thermocycling parameters were - initial denaturation at 95°C for 15 minutes, followed by 30 cycles at 94°C for 20 seconds, 55°C for 20 seconds and 72°C for 40 seconds. The final soak time was kept for 10 minutes at 72°C. Restriction enzyme digestion was carried out by incubating 5 µl of the PCR product with 10 units of Fast Digest *Bam*HI enzyme (MBI Fermentas, USA) in a total reaction volume of 10 µl, for 15 minutes at 37°C. After digestion was completed, capillary electrophoresis on the ABI 3130 DNA Sequencer was performed by loading a mixture of 0.75µl of the enzyme digested PCR product with 0.25 µl of ABI Genescan 500 LIZ size standard (Applied Biosystems, USA) and 9 µl of deionized formamide. Genemapper v3.7 (Applied Biosystems, USA) was used to size the fluorescent dye labeled enzyme digested bands. Cleavage of the 187 bp PCR product into two bands of 120 and 67 bp was diagnostic in tiger (which possesses the restriction site) but not leopard, which lack the cleavage site (Figure S1A).

**Results**

Amplification of a 187 bp cytochrome *b* gene fragment using the PtiL-15,648 and PtiH-15,833 primers was successful in both tigers and leopards, but amplification failed in other non-felid carnivores and all potential prey species (Table S10). The control 309 bp universal cyt *b* primers showed amplification with all samples proving that non-amplification of the 187 bp band in non-felid species is because of sequence differences at the priming site and not a result of artifacts or stochastic error in amplification. *Bam*HI restriction enzyme digestion of the 187 bp long PCR product yielded two diagnostic bands of 120 and 67 bp sizes in tiger, but not in leopard (Figure S1B). Since leopards lack the restriction site at the particular position of the cyt *b* gene compared to tigers, the band profile of the species on enzyme digestion of the PCR products was identical to the undigested PCR product. The tests were conducted using blood samples and demonstrate the reliability of the PCR-restriction digestion protocol to distinguish between the two sympatric species (Table S10) Performance of the primers was high in scats, with 94% (61 out of 65 scats) amplification success. Of the 61 successfully amplified scat DNA extracts, 51 were assigned to tiger based on *Bam*HI digestion profiles, while the remaining 10 were assigned to leopard (Table S11).

**Supplementary Method S1 References**

Mukherjee N, Mondol S, Andheria A and Ramakrishnan U (2007) Rapid multiplex PCR based species identification of wild tigers using non-invasive samples. Conserv Genet 8: 1572- 9737. doi: 10.1007/s10592-007-9289-z.

Mukherjee S, Ashalakshmi CN, Home C and Ramakrishnan U (2010) An evaluation of the PCR-RFLP technique to aid molecular-based monitoring of felids and canids in India. BMC Res Notes 3: 159. doi: 10.1186/1756-0500-3-159.

Nagata J, Aramilev VV, Belozor A, Sugimoto T and McCullough DR (2005) Fecal genetic analysis using PCR-RFLP of cytochrome b to identify sympatric carnivores, the tiger *Panthera tigris* and the leopard *Panthera pardus*, in far eastern Russia. Conserv Genet 6: 863– 865. doi 10.1007/s10592-005-9038-0.

Rozen S and Skaletsky HJ (2000) PRIMER 3 on the WWW for general users and for biologist programmers. In Bioinformatics Methods and Protocols: Methods in Molecular Biology (edsKrawetz, S. and Misener, S.), Humana Press, Totowa, New Jersey. pp. 365– 386. Available: http://bioinfo.ut.ee/primer3/. Accessed 10 Jun 2010.

Sugimoto T, Nagata J, Aramilev VV, Belozor A, Higashi S and McCullogh D (2006) Species and sex identification from faecal samples of sympatric carnivores, Amur leopard and Siberian tiger, in the Russian Far East. Conserv Genet 7: 799- 802. doi: 10.1007/s10592-005-9071-z.
