# Supplementary material for: Prioritizing Tiger Conservation through Landscape Genetics and Habitat Linkages
Source: PLoS One. 2014 Nov 13;9(11):e111207. doi: 10.1371/journal.pone.0111207 (PMC4230928; doi:10.1371/journal.pone.0111207)
Supplement: Method S2 — Generating the cost surface for PATHMATRIX and CIRCUITSCAPE. (DOCX) [file pone.0111207.s017.docx]

**Supplementary Method S2. Generating the cost surface for PATHMATRIX and CIRCUITSCAPE**

The model inferred grid (10x10 km^2^) occupancy probability was transferred to the forested habitat within that grid. This occupancy probability incorporates effects of habitat, prey availability, and human disturbance as well as corrects for imperfect detection of tiger sign. Since forests were not contiguous, a gap in forest patches would result in a barrier to tiger movement across it. Field data suggests that tigers can occasionally traverse such gaps in forest contiguity. Often tigers were observed to use drainage topography under the cover of darkness to travel from one forest patch to another patch. To mimic this ecological reality we added the following values to the habitat categories to allow some possibility of tiger movement across them:

1. All background pixels were given a value of 0.001 at a 1 km^2^ resolution.
2. 1 km^2^ pixels that contained drainage were further given a value of 0.005.
3. All 1 km^2^ pixels that contained forest habitat were given a value of 0.01
4. Pixels that contained nightlights were forced to have a value of zero i.e. human habitation were considered barriers to movement of tigers.

The conductance of a 1 km^2^ pixel was computed as C = a + b + c + Occupancy probability of forested habitat. The resistance value of a 1 km^2^ pixel was computed as R = 1-C.

The resultant layer of conductance C or Resistance R was used as input layer to compute least cost pathways and current flow (tiger movement) between tiger reserves in PATHMATRIX (Ray 2005) and CIRCUITSCAPE (McRae and Shah 2009).

**Supplementary Method S2 References**

Ray N (2005) PATHMATRIX: a geographical information system tool to compute effective distances among samples. Mol Ecol Notes 5: 177- 180. doi:10.1111/j.1471-8286.2004.00843.x.

McRae BH and Shah VB (2009) Circuitscape users’ guide online. The University of California, Santa Barbara. Available: <http://www.circuitscape.org>. Accessed 10 Oct 2013.
